# A review of the land snail genus *Alycaeus* (Gastropoda, Alycaeidae) in Peninsular Malaysia

**DOI:** 10.3897/zookeys.692.14706

**Published:** 2017-08-21

**Authors:** Junn Kitt Foon, Thor-Seng Liew

**Affiliations:** 1 Institute for Tropical Biology and Conservation, Universiti Malaysia Sabah, Jalan UMS, 88400 Kota Kinabalu, Sabah, Malaysia; 2 Rimba, 22-3A, Casa Kiara 2, Jalan Kiara 5, 50480 Kuala Lumpur, Malaysia

**Keywords:** land snail, shell morphology, operculum, Alycaeinae, limestone karsts, Southeast Asia

## Abstract

A total of 11 species and 1 subspecies of *Alycaeus* were recognised in Peninsular Malaysia prior to this study. However, these taxonomic descriptions of *Alycaeus* taxa were based on limited numbers of examined materials, where a whole spectrum of morphological variations were not accounted for and diagnoses were often provided without sufficient comparison between congeners from across the peninsula. We reviewed Peninsular Malaysian *Alycaeus* through the examination of 5137 specimens in 522 collection lots from all major museum collections and literature sources. Based on these examined materials, we utilised a more comprehensive revised set of 39 shell and operculum characters, as well as living animal colour to describe all *Alycaeus* species in this paper. We also noted their habitat and ecology, as well as updated the distribution of each species. Of the 12 previously described taxa, 10 are reconfirmed as present on Peninsular Malaysia (*Alycaeus balingensis*, *Alycaeus carinata*, *Alycaeus conformis*, *Alycaeus gibbosulus*, *Alycaeus kapayanensis*, *Alycaeus kelantanensis*, *Alycaeus liratulus*, *Alycaeus perakensis perakensis*, *Alycaeus perakensis altispirus* and *Alycaeus thieroti*) and 2 are confirmed as absent from the peninsula (*Alycaeus jagori* and *Alycaeus pyramidalis*). A new record of *Alycaeus robeleni* is reported for Peninsular Malaysia. One species, *Chamalycaeus jousseaumei* is confirmed as present on the peninsula and is reassigned to *Alycaeus*. The subspecies *Alycaeus perakensis altispirus* Möllendorff, 1902, is elevated to species. Examined Peninsular Malaysian materials that do not fit previously recognised species are described as new species. A total of 11 new species are proposed (*Alycaeus selangoriensis* **sp. n.**, *Alycaeus costacrassa* **sp. n.**, *Alycaeus ikanensis* **sp. n.**, *Alycaeus alticola* **sp. n.**, *Alycaeus charasensis* **sp. n.**, *Alycaeus kurauensis* **sp. n.**, *Alycaeus regalis* **sp. n.**, *Alycaeus virgogravida* **sp. n.**, *Alycaeus senyumensis* **sp. n.**, *Alycaeus expansus* **sp. n.**, *Alycaeus clementsi* **sp. n.**). Overall, 23 species of *Alycaeus* are now recognised in Peninsular Malaysia.

## Introduction

The family Alycaeidae Blanford, 1864, is a distinct group of operculated land snails defined by their globular, conical or discoid shell, whorl constriction prior to the aperture and a conspicuous tube at the suture (‘breathing tube’) which runs from the constriction to whorls prior to the constriction (Godwin-Austen 1889, Kobelt 1902, Egorov 2013). Within Alycaeidae, the genus *Alycaeus* Baird, 1850, is separated into three subgenera – *Alycaeus* Baird, 1850, *Pincerna* Preston, 1907, and *Stomacosmethis* Bollinger, 1918 (Egorov 2013). However, all of these subgenera [including *Pincerna* (see Páll-Gergely 2017)] remain inadequately diagnosed and will require extensive revision, a task that is beyond the scope of this study. As such, this review will solely focus on species in Peninsular Malaysia that are placed under the genus *Alycaeus* including two species recently reassigned under *Pincerna* (Páll-Gergely 2017) – *Alycaeus liratulus* Preston, 1907, and *Alycaeus thieroti* Morgan, 1885b, and one species formerly assigned under *Chamalycaeus* (Kobelt & Möllendorff, 1897) – *Alycaeus jousseaumei* Morgan, 1885a.

In Peninsular Malaysia, 11 species and 1 subspecies of *Alycaeus* were recognised prior to this study (Maassen 2001, Maassen 2006), namely *Alycaeus balingensis* Tomlin, 1948, *Alycaeus carinata* Maassen, 2006, *Alycaeus conformis* Fulton, 1902, *Alycaeus gibbosulus* Stoliczka, 1872, *Alycaeus jagori* Martens, 1859, *Alycaeus kapayanensis* Morgan, 1885b, *Alycaeus kelantanensis* Sykes, 1902, *Alycaeus liratulus* Preston, 1907, *Alycaeus perakensis perakensis* Crosse, 1879a, *Alycaeus perakensis altispirus* Möllendorff, 1902, *Alycaeus pyramidalis* Benson, 1856 (as *Dioryx pyramidalis*, see Habe 1965) and *Alycaeus thieroti* Morgan, 1885b.

Most past studies of *Alycaeus* in Peninsular Malaysia are either single species descriptions or checklists. There are only two studies that focused on the anatomy and radula of *Alycaeus* (Tielecke 1940, Venmans 1956) and three that focused on conservation biogeography of karst land snails (Davison and Kiew 1990, Clements et al. 2008, Foon et al. 2017). Besides these five non-conchological studies, all other *Alycaeus* taxonomic treatments to date were based on inconsistent and subjective diagnoses of few shell and operculum characters which have never been properly reviewed since their description or in the revisions by Kobelt (1902) and Egorov (2013). Even when detailed decriptions were given for a species (e.g. *Alycaeus perakensis* Crosse, 1879a, *Alycaeus carinata* Maassen, 2006), no comprehensive comparisons with other Peninsular Malaysian *Alycaeus* species were effected until now.

In view of this, we reviewed all *Alycaeus* species in Peninsular Malaysia using a revised set of 39 shell and operculum characters, as well as living animal colour. We consulted major museum collections in Europe, the United States of America, Singapore and Malaysia that house *Alycaeus* materials from Peninsular Malaysia, including types. A large collection of recently sampled *Alycaeus* from across Peninsular Malaysia housed in the *BORNEENSIS* collection, Universiti Malaysia Sabah, were also studied.

Based on this review, we reconfirmed the presence of 10 previously recognised species on the peninsula (*A. balingensis*, *A. carinata*, *A. conformis*, *A. gibbosulus*, *A. kapayanensis*, *A. kelantanensis*, *A. liratulus*, *A. perakensis perakensis*, *A. perakensis altispirus* and *A. thieroti*) while 2 species are confirmed as absent on the peninsula (*A. jagori* and *A. pyramidalis*). For *Alycaeus jagori*, records of the species in Perak, Peninsular Malaysia were reported without further elaboration by some workers (Kobelt 1902, Laidlaw 1928, Schilthuizen et al. 1999, Maassen 2001, parts of BOR/MOL collection lots). Benthem Jutting (1948) expressed reservations about the validity of these Peninsular Malaysian records. We examined the syntype of *Alycaeus jagori* (SMF 109304/1) and concluded that it is indeed distinguished from any Peninsular Malaysian congeners in spire shape, whorl convexity, whorl sculpture and shell size. As such, *Alycaeus jagori* is herein confirmed as non-existent on Peninsular Malaysia. The source of this mislocation most likely originated from Kobelt and Möllendorff (1897) whereby past workers may have confused the locality of *A. jagori* (Java) with *A. kapayanensis* (Perak), both of which were listed next to each other in the text. For *Alycaeus pyramidalis*, the mislocation is traced back to a mistake by Habe (1965) in which specimens of *A. gibbosulus* from northern Malay Peninsula were misidentified as *A. pyramidalis*. Also, a new record of *Alycaeus robeleni* is reported for Peninsular Malaysia. One species, *Chamalycaeus jousseaumei* is confirmed as present on the peninsula and is reassigned to *Alycaeus*. The subspecies *Alycaeus perakensis altispirus* Möllendorff, 1902, is elevated to species. Overall, we redescribed all 12 previously known species of *Alycaeus* on Peninsular Malaysia.

Finally, we proposed and described 11 new species based on examined *Alycaeus* materials from Peninsular Malaysia that do not fit into previously recognised species: *Alycaeus selangoriensis* sp. n., *Alycaeus costacrassa* sp. n., *Alycaeus ikanensis* sp. n., *Alycaeus alticola* sp. n., *Alycaeus charasensis* sp. n., *Alycaeus kurauensis* sp. n., *Alycaeus regalis* sp. n., *Alycaeus virgogravida* sp. n., *Alycaeus senyumensis* sp. n., *Alycaeus expansus* sp. n., *Alycaeus clementsi* sp. n. Overall, 23 species of *Alycaeus* are now recognised in Peninsular Malaysia.

## Materials and methods

### Study site

This study focuses solely on Peninsular Malaysia, with emphasis on limestone karsts where the majority of study materials were obtained. Peninsular Malaysia is situated at the lower end of the Malay Peninsula, which is also politically divided into Thailand and Myanmar in the north. Peninsular Malaysia is at the centre of the Tropical East Asian region and hence shares similar fauna with South and East Asia (Corlett 2014), including the genus *Alycaeus* (Kobelt 1902, Egorov 2013). Peninsular Malaysia is characterised by parallel, old (circa 200 million years old) and tall (circa 1000–2000 m high) non-limestone mountain ranges aligned at a north-south axis, separating flat alluvial and knolled valleys (Paton 1961, Metcalfe 2013). Within these valleys, limestone bedrocks of various ages are occasionally exposed as disjunct or continous limestone tower karsts (Paton 1961). These tower karsts have been exposed for millions of years and have gradually become isolated from each other through weathering and erosion (Benthem Jutting 1960b, Paton 1961, Twidale 2006). As such, ecosystems on limestone karsts are well known to be analogous to island ecosystems and have high species endemism including land snails (Clements et al. 2006, Liew et al. 2014).

### Literature, fieldwork and collection data management

For literature review, the term “*Alycaeus*” was used to obtain publications from the Zoological Record, Google Scholar and Biodiversity Heritage Library (on 15 March 2016 and 30 May 2017). All publications mentioning *Alycaeus* species from Peninsular Malaysia were studied and reviewed.

The management of data for examined materials follows Liew et al. (2014). Materials from BOR/MOL and ZRC were personally examined by the first author. Materials elsewhere and their associated measurements and photographs were obtained either through existing digitised museum collections or with help from curators (MNHN – Manuel Caballer and Virginie Héros, NHM – Jonathan Ablett) and visiting scientists (SMF – Páll-Gergely Barna, MNHN – Gopalasamy Reuben Clements, ANSP – Siong Kiat Tan, RMNH/ZMA – Thor-Seng Liew). The number of shells for each collection lot examined were indicated after the virgule that follows after the accession number.

The locality for each collection lot was identified and linked to its current name whenever possible. For limestone karst localities, we followed the standard format of code and name (e.g. PRK 18 Gunung Lanno) used in the limestone hill register by Liew et al. (2016). Place names in this paper contain Malay words, namely: Batu = rock; Bukit/Buket = hill; Gua = cave; Gunung/Gunong = mountain; Kota = large tower karsts or fortress; Kuala/Kwala = estuary or river confluence; Pulau = island; Sungai/Sungei = river; Tasik = lake; Wang = large doline or valley surrounded on all sides by tower karsts. Geographical coordinates for the type locality of each species was provided except when the stated type locality was not exact i.e. only state-level or island names were given as the type locality. The locality records for each species based on materials examined are shown in Figures 2, 3, 4, 5 and 6.

In addition to the previously collected museum materials, a systematic sampling of *Alycaeus* at 80 localities across Peninsular Malaysia was conducted in 2016 (for full list of localities, see Suppl. material 1). During the sampling, about 45 to 60 minutes were spent at each locality to search for living and dead *Alycaeus* individuals on all habitats (in the leaf litter, under rotten logs, on vegetation and on rocky substrates). Up to 20 individuals per species were observed and collected whenever possible. Animal colouration, habitat and other ecological observations were noted.

### Collection repositories

**ANSP** Academy of Natural Sciences of Drexel University, Philadelphia, United States of America.

**BOR/MOL** BORNEENSIS Malacology Collection, Institute for Tropical Biology and Conservation, Universiti Malaysia Sabah, Kota Kinabalu, Malaysia.

**ANSP** Chulalongkorn University Zoological Museum, Bangkok, Thailand.

**HUJ** National Mollusc Collection of the Hebrew University of Jerusalem, Jerusalem, Israel.

**HW** Private collection of Jens and Christa Hemmen, Wiesbaden, Germany.

**MNHN** Museum National d’Histoire Naturelle, Paris, France.

**NHM** The Natural History Museum, London, United Kingdom.

**NHMUK** When citing NHM registered specimens.

**RMNH** Naturalis Biodiversity Center (formerly Rijksmuseum van Natuurlijke Historie), Leiden, the Netherlands.

**SMF** Forschungsinstitut und Naturmuseum Senckenberg, Frankfurt am Main, Germany.

**ZMA** Naturalis Biodiversity Center (formerly Zoological Museum of Amsterdam), Leiden, the Netherlands.

**ZRC** Zoological Reference Collection, Lee Kong Chian Natural History Museum, National University of Singapore, Singapore.

### Species description and delimitation

**Figure 1.**
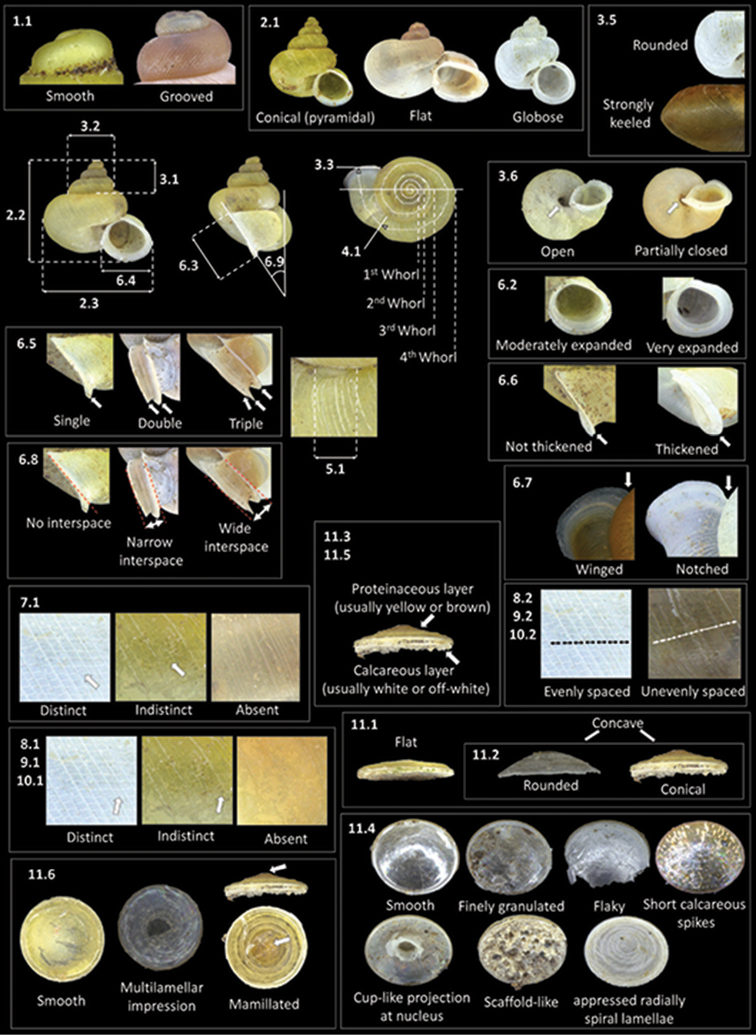
An illustrated synopsis of the framework for species description based on 39 shell and operculum features (excluding shell colour and living animal colour). Number codes ascribed to each character are linked to descriptions and measurement methods outlined in Table 1.

To date, most of the *Alycaeus* species in Peninsular Malaysia have been described and diagnosed using inconsistent, subjective, and descriptive conchological and opercular characters with little or no comparison with other congeners. No more than 7 characters were used in previous studies including (1) breathing tube shape and size, (2) aperture and peristome shape, (3) constriction shape, (4) whorl shape, (5) radial and spiral sculpture, (6) operculum characters and (7) shell shape (Godwin-Austen 1889, Kobelt 1902). These characters have not been reviewed since Kobelt (1902) and thus, their usefulness as diagnostic characters for species is unverified. Furthermore, phenotypic plasticity in *Alycaeus* shell features has not been studied although it is known in other terrestrial Caenogastropoda (Berry 1962, Liew et al. 2014). Thus, we employed a revised comprehensive assessment of 39 shell and operculum characters with empirical data, including living animal colour (Table 1, Figure 1). Living animal colour has been shown to be useful for distinguishing species in some caenogastropod genera (Reid 2007, 2014). However, in studies of the terrestrial caenogastropod genus *Plectostoma* Adam, 1865, intraspecific shell colour differences have been linked to sexual dimorphism (Schilthuizen et al. 2003). Given that no sexual dimorphism study has been done on *Alycaeus* to date and sex was not determined for the materials examined in this study, shell colour differences in *Alycaeus* will be described but not used for species diagnosis in this study.

**Table 1.** Framework for species description based on 39 shell and operculum features, as well as living animal colour. Number codes are ascribed to each character and linked to illustrations in Figure 1 for visualisation of character descriptions and measurements, except shell colour and living animal colour.

Morphological section	Character	Descriptions and measurements
1. Protoconch	1.1 Sculpture seen at 5.0 × magnification	Smooth or grooved.
2. Shell shape and size	2.1 Shell shape	Conical (pyramidal), flat or globose.
	2.2 Shell height	Measured from apex to base of ultimate whorl parallel to coiling axis (in millimetres to the nearest 0.01 mm).
	2.3 Shell width	Measured at outermost edges of shell including aperture, perpendicular to coiling axis (in millimetres to the nearest 0.01 mm).
3. Spire	3.1 Spire height	Measured from apex to base of penultimate whorl at suture (in millimetres to the nearest 0.01 mm) (after Liew et al. 2014).
	3.2 Spire width	Measured at outermost edges of penultimate whorl perpendicular to coiling axis (in millimetres to the nearest 0.01 mm) (after Liew et al. 2014).
	3.3 Number of whorls	Maximum number of whorls posterior of protoconch (after Vermeulen and Whitten 1998). Non-integers are represented as fractions of 8 (⅛ to ⅞) for easier understanding (modified from Vermeulen and Whitten 1998).
	3.4 Spire shape	Oblong conical (width > height) (after Liew et al. 2014).
	3.5 Whorl periphery	Strongly keeled or rounded.
	3.6 Umbilicus	Open or partially closed (i.e. partially blocked by penultimate whorl and/or peristome extension at columella).
4. Whorl constriction	4.1 Position in whorl	Approximate position based on number of whorls posterior of protoconch. Non-integers are represented as fractions of 8 (⅛ to ⅞) for easier understanding (modified from Vermeulen and Whitten 1998).
5. Breathing tube	5.1 Length	Length from posterior end to anterior end (in millimetres to the nearest 0.01 mm).
6. Aperture and peristome	6.1 Aperture shape	Circular.
	6.2 Aperture margin	Margins moderately expanded or very expanded.
	6.3 Aperture height	Measured at outermost edges of aperture from the palatal to the basal side (in millimetres to the nearest 0.01 mm). Measurement made with aperture tilted parallel to the observer (after Liew et al. 2014)
	6.4 Aperture width	Measured at outermost edges of aperture from columella to palatal side (in millimetres to the nearest 0.01 mm). Measurement made perpendicular to coiling axis.
	6.5 Peristome types	Single, double or triple.
	6.6 Peristome thickness	Thickened or not thickened.
	6.7 Peristome shape	Notched or winged at suture. Upper palatal section sometimes folded posteriorly.
	6.8 Peristome spacing	No interspace (inner peristome impressed flatly on outer peristome), narrow interspace (inner peristome slightly protruded posteriorly beyond outer peristome), wide interspace (inner peristome protruded posteriorly far from outer peristome, creating a new section of whorl) (after Yamazaki et al. 2013).
6. Aperture and peristome	6.9 Peristome orientation	Degrees of obliquity with respect to the coiling axis of the spire.
7. Spiral lines	7.1 Spiral line seen at 10.0 × magnification	Distinct (protruded from shell surface), indistinct (less protruded from shell surface) or absent.
	7.2 When present, spiral lines’ spacing	Spacing between lines regular or irregular. Number of spiral lines per 1 millimetre.
8. Radial ribs running anterior of breathing tube	8.1 Radial ribs seen at 4.0 × magnification	Pronounced (i.e. protruded from shell surface), indistinct (i.e. less protruded from shell surface), or absent (i.e. replaced with radial growth lines).
	8.2 When present, radial ribs’ spacing	Evenly or unevenly spaced. Number of radial ribs per 1 milimetre.
9. Radial ribs running perpendicular to breathing tube	9.1 Radial ribs seen at 4.0 × magnification	Pronounced (i.e. protruded from shell surface), indistinct (i.e. less protruded from shell surface), or absent (i.e. replaced with radial growth lines). Thick or not thick.
	9.2 When present, radial ribs’ spacing	Evenly or unevenly spaced. Number of radial ribs per 1 milimetre.
10. Radial ribs running posterior of breathing tube	10.1 Radial ribs seen at 4.0 × magnification	Pronounced (i.e. protruded from shell surface), indistinct (i.e. less protruded from shell surface), or absent (i.e. replaced with radial growth lines). Thick or not thick.
	10.2 When present, radial ribs’ spacing	Evenly or unevenly spaced. Number of radial ribs per 1 milimetre.
11. Operculum	11.1 Concavity	Concave (exterior cave-in) or flat.
	11.2 Shape	Rounded or conical.
	11.3 Exterior composition	Proteinaceous or calcareous layered.
	11.4 Exterior sculpture	Smooth, finely granulated, flaky, short calcareous spikes, cup-like projection at nucleus, scaffold-like calcareous deposits or appressed radially spiral lamellae.
	11.5 Interior composition	Proteinaceous or calcareous layered.
	11.6 Interior sculpture	Smooth, multilamellar impression, mamillate.
12. Shell colour	12.1 Shell colour	White, pinkish-white, pink, yellow, orange, red, brownish-red. brown, purple.
13. Living animal	13.1 Body colour	Cream-white, yellow, cream-yellow, light brown, brown, maroon, greenish-black, light grey, grey.
	13.2 Head colour	Orange, pink, pinkish-brown, brown, maroon, greenish-black, grey, dark grey.
	13.3 Tentacles colour	Uni-coloured (yellow, red, brown, reddish-brown, light grey, dark grey, black), or bi-coloured (green with brown tips, cream-white with pink tips, yellow with red tips).

To assist with visualising the descriptions, we provided photographs of five standard shell views (apertural, lateral, apical, umbilical and dorsal/oblique dorsal), three close-up shell views (breathing tube, apical whorls, radial rib and spiral line sculpture) and four standard operculum views (exterior, interior, side and oblique exterior) for each species. More than one specimen is illustrated for each species to represent the intraspecific variability if required. A synoptic plate of shells at apertural view representing the 23 *Alycaeus* taxa in Peninsular Malaysia (Figure 31) is provided to compare shell size and shape.

We measured up to 28 shells representing the whole spectrum of variation in shell height, shell width, spire height, spire width, aperture height and aperture width for each species to gauge intraspecific variation of shell dimensions against interspecific variation (Table 1, Figure 1). In addition to this, up to 9 shells representing the whole spectrum of shell shape and sizes for each species were measured for the number of whorls, position of whorl constriction, length of breathing tube, spiral line spacing, radial ribs spacing and peristome orientation (Table 1, Figure 1). The variations are summarised in the species description (for raw data of individual shell measurements, see Suppl. material 2). Type materials available to us were also measured. They were then assigned to respective species based on the assessment framework in Table 1. Overall, materials examined totalled 5137 specimens from 522 collection lots which is comprised of: ANSP – 9 collection lots (including 1 type lot), 22 specimens in total; BOR/MOL – 411 collection lots (including 48 type lots), 3031 specimens in total; MNHN – 6 collection lots (including 6 type lots), 10 specimens in total; NHM – 3 collection lots (including 3 type lots), 5 specimens in total; RMNH – 13 collection lots (including 2 type lots), 137 specimens in total; SMF – 2 collection lots (including 2 type lots), 2 specimens in total; ZMA – 5 collection lots, 7 specimens in total; ZRC – 73 collection lots, 1923 specimens in total.

Overall, we employed the morphological species concept in this study as our comprehensive 39 characters assessment on a large sample size enables us to understand intraspecific variation of shell dimensions against interspecific variation. In addition to this, we also follow previous studies in recognising the major role of limestone hill isolation and biogeography in vicariance and speciation of karst restricted taxa especially land snails in Peninsular Malaysia (Clements et al. 2008, Hoekstra and Schilthuizen 2011, Liew et al. 2014). Notably, a study of another limestone restricted genus *Plectostoma* inhabiting limestone hills that are near each other but separated by biogeographic barriers may only differ a little in morphology but is sufficiently genetically distinct to be considered as different species (Liew et al. 2014). Given that many of the *Plectostoma* species in Liew et al. (2014) were sampled from the same localities as *Alycaeus* in this study and that most *Alycaeus* species are also restricted to limestone areas, it is very likely that *Alycaeus* in this study will show the same trend of morphological variation and speciation as *Plectostoma*. To conclude, this study represents the first attempt at a systematic taxonomic review of *Alycaeus* species in Peninsular Malaysia and has used a large quantity of materials and characters to arrive at an empirically supported species hypothesis. The emergence of additional evidences such as genetics and anatomy in the future will be useful to further assess these species hypotheses.

### Shell and operculum characters

The description of shell and operculum characters partially follows the terminology established by Godwin-Austen (1889) and Kobelt (1902). However, a revised set of 39 shell and opercular characters are introduced in this study to provide a comparative morphological framework with objective empirical data for all *Alycaeus* specimens that were examined in this study (Table 1, Figure 1). All measurements are in millimetres and are rounded up to the nearest 0.01 mm. Most of the characters can be observed by stereomicroscope at 2.0 × magnification except protoconch sculpture, spiral lines and radial ribs (some species) which can only be seen with at least 4.0 × magnification. Two archaic terms have also been replaced based on latest findings: (1) “horny operculum” (Godwin-Austen 1889) to proteinaceous operculum (see Checa and Jiménez-Jiménez 1998), (2) “sutural tube” (Godwin-Austen 1889) to breathing tube (see Páll-Gergely et al. 2016).

**Figure 2.**
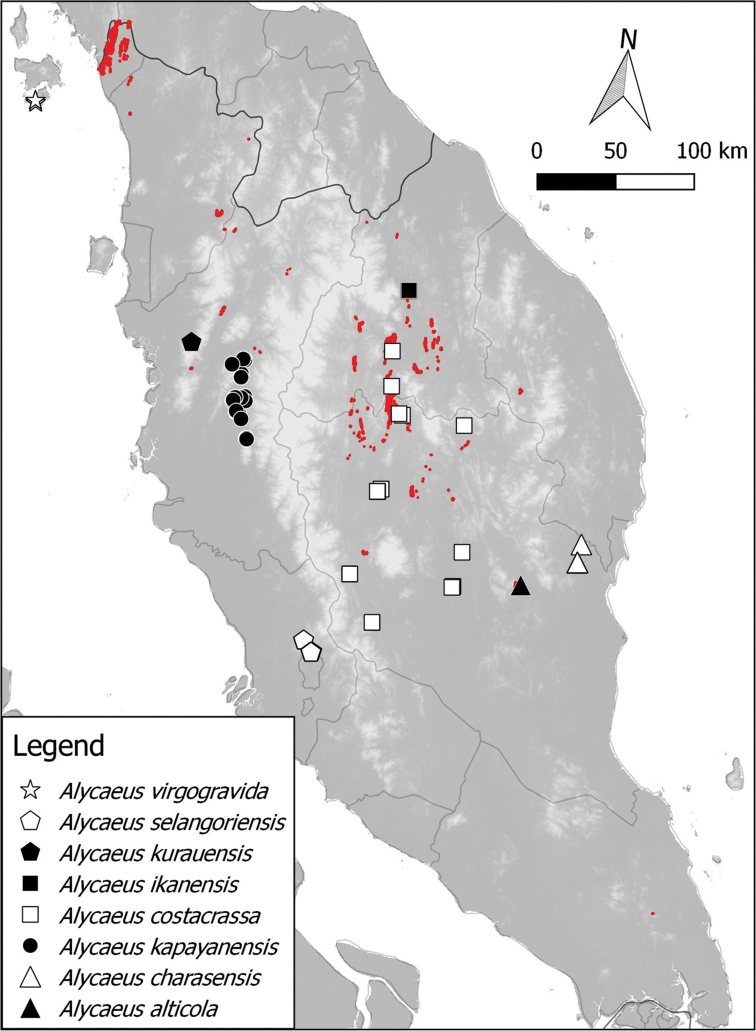
Distribution of 8 *Alycaeus* species in Peninsular Malaysia based on materials examined. *Alycaeus altispirus* is not included in the maps because no specific locality was given for the materials examined (see Discussion section under the species). Note the red areas are limestone karsts (derived from Liew et al. 2016).

**Figure 3.**
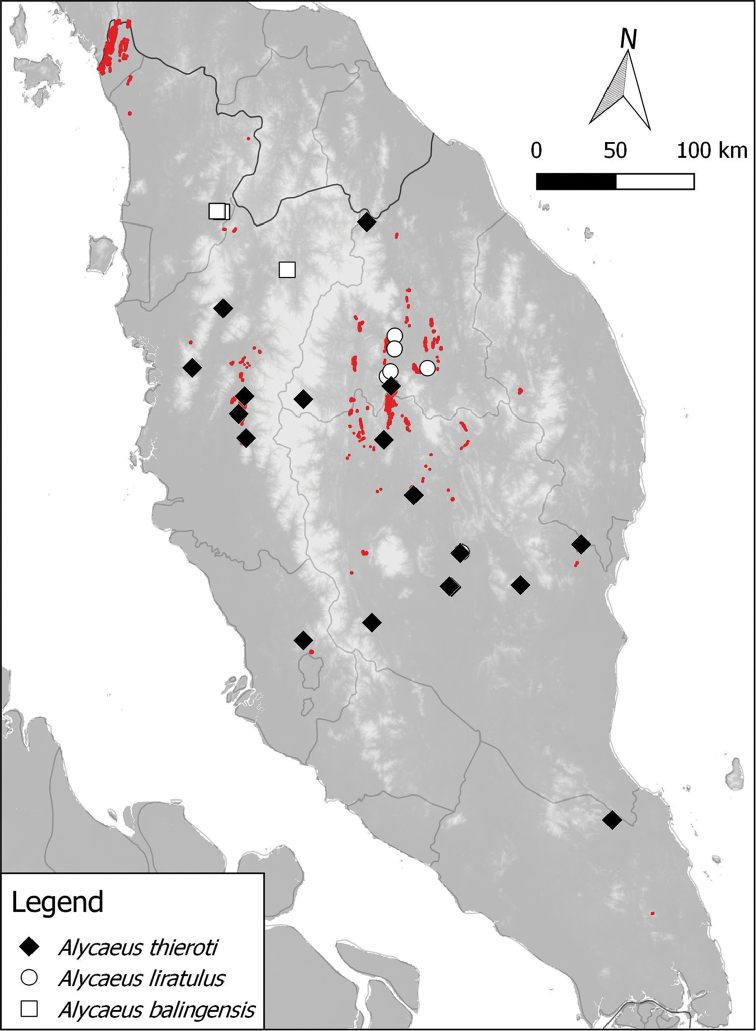
Distribution of 3 *Alycaeus* species in Peninsular Malaysia based on materials examined. Note the red areas are limestone karsts (derived from Liew et al. 2016).

**Figure 4.**
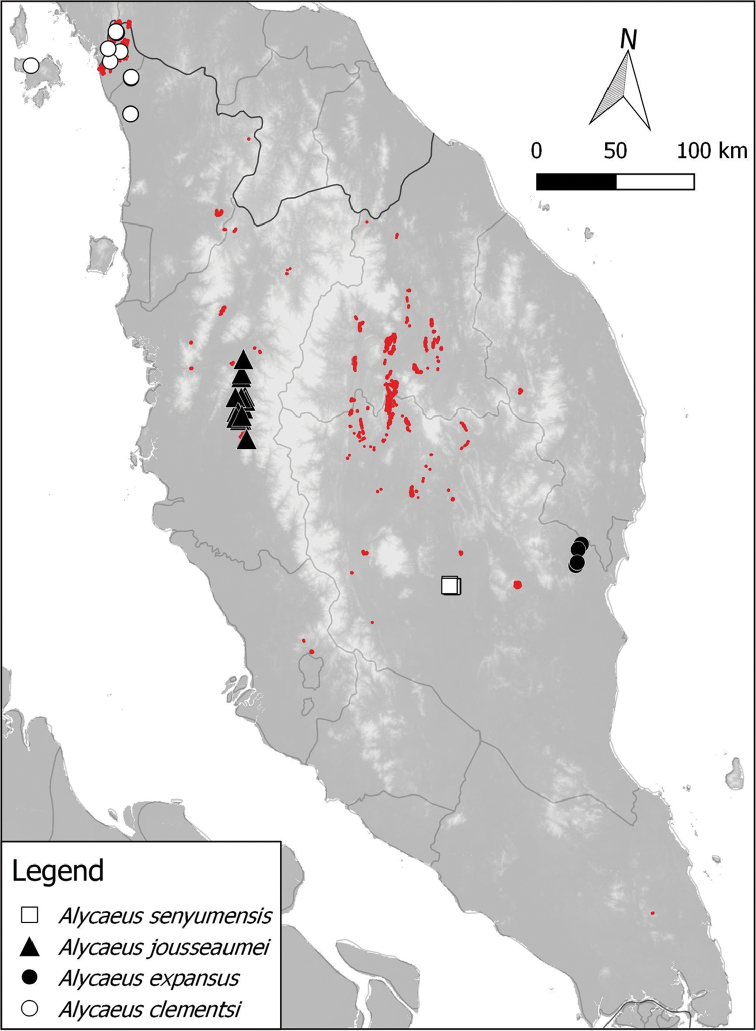
Distribution of 4 *Alycaeus* species in Peninsular Malaysia based on materials examined. Note the red areas are limestone karsts (derived from Liew et al. 2016).

**Figure 5.**
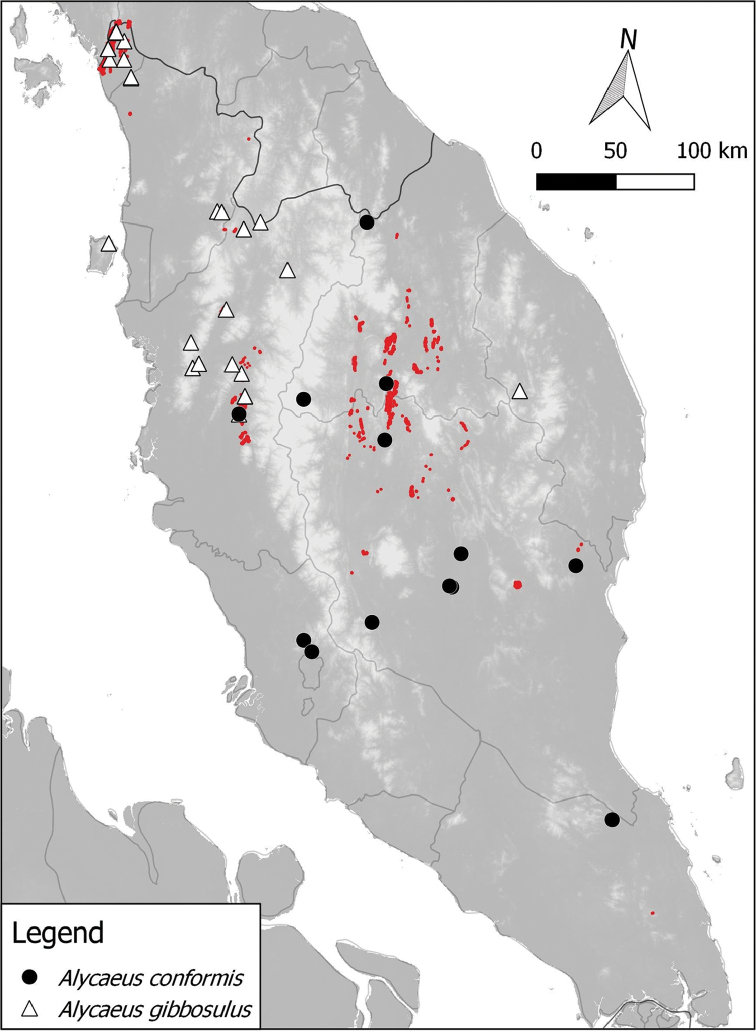
Distribution of 2 *Alycaeus* species in Peninsular Malaysia based on materials examined. Note the red areas are limestone karsts (derived from Liew et al. 2016).

**Figure 6.**
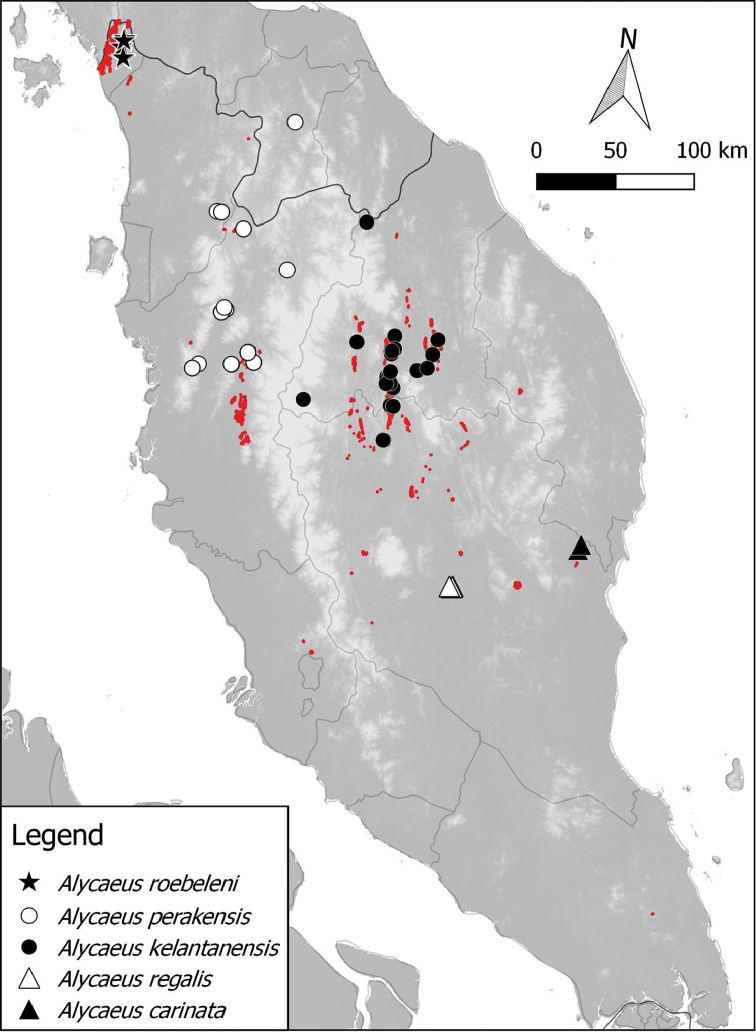
Distribution of 5 *Alycaeus* species in Peninsular Malaysia based on materials examined. Note that two records of *Alycaeus roebeleni* in Thailand, north of Peninsular Malaysia are not shown in the map. The red areas are limestone karsts (derived from Liew et al. 2016).

## Systematic part

### Class Gastropoda Cuvier, 1797

#### Family Alycaeidae Blanford, 1864

##### Genus Alycaeus

Taxon classificationAnimaliaGastropodaAlycaeidae

Baird, 1850

Alycaeus Baird, 1850: 27–28.

###### Description for Peninsular Malaysian species.

**Protoconch.** Smooth. In some species, second whorl grooved.

**Shell shape and size.** Conical (pyramidal), flat or globose. Shell height: 3.03–10.91 mm. Shell width: 3.36–15.04 mm.

**Spire.** Spire height: 0.93–3.21 mm. Spire width: 1.40–4.00 mm. Number of whorls: up to 3–5 ½. Spire shape always oblong conical. Whorl periphery strongly keeled or rounded. Always umbilicated. Umbilicus open or partially closed.

**Whorl constriction.** Position of constriction about 2 ¼–5 ¼ whorls posterior of protoconch.

**Breathing tube.** Always present, varies in length: 0.29–6.52 mm.

**Aperture and peristome.** Aperture always circular. Apertural margin moderately expanded or very expanded. Aperture height: 1.59–7.30 mm. Aperture width: 1.60–6.59 mm. Peristome single, double or triple. Peristome thickened or not thickened. Peristome notched or winged at suture. Interspace between peristome none, narrow or wide. Peristome orientation varies 4°–55° oblique to the coiling axis.

**Spiral lines.** Absent or present. When present, distinction varies (distinct or indistinct), spacing always regular to irregular. Number of spiral lines: 11–60 lines per 1 mm.

**Radial ribs running anterior of breathing tube.** Pronounced, indistinct or absent. Even or unevenly spaced. 6–21 radial ribs per 1 mm.

**Radial ribs running perpendicular to breathing tube.** Pronounced, indistinct or absent. Radial ribs sometimes are thicker and whiter compared to radial ribs anterior of breathing tube. Even or unevenly spaced. 6–29 ribs per 1 mm.

**Radial ribs running posterior of breathing tube.** Pronounced, indistinct or absent. Usually unevenly spaced. 2–25 ribs per 1 mm.

**Operculum.** Concave or flat. Rounded or conical. Exterior usually calcareous layered of variable thickness. Exterior texture varies (smooth, finely granulated, flaky, short calcareous spikes, cup-like projection at nucleus, scaffold-like calcareous deposits or appressed radially spiral lamellae). Interior usually proteinaceous layered. Interior sculpture smooth, with multilamellar impression or mamillated.

**Shell colour.** Varies between and within species (white, pinkish-white, pink, yellow, orange, red, brownish-red. brown, purple). Apical whorls either in white or non-white colours, always fading to white in subsequent whorls.

**Living animal.** Body colour variable but lighter than head and tentacle (cream-white, yellow, cream-yellow, light brown, brown, maroon, greenish-black, light grey, grey). Head colour variable (orange, pink, pinkish-brown, brown, maroon, greenish-black, grey, dark grey). Tentacle either uni-coloured (yellow, red, brown, reddish-brown, light grey, dark grey, black), or bi-coloured (green with brown tips, cream-white with pink tips, yellow with red tips). Head and tentacle often darker coloured relative to the body.

###### Habitat and ecology.

Three distinct habitats: rock (crevices, rock walls, solution holes, moss and lichen covered rocks), vegetation (low shrubs, tree trunks) or forest floor (rotten logs, leaf litter). Usually in moist areas but ocassionally found in drier areas. Always in forested habitats.

###### Remarks.

The genus *Alycaeus* Baird, 1850, is separated into three subgenera – *Alycaeus* Baird, 1850, *Pincerna* Preston, 1907, and *Stomacosmethis* Bollinger, 1918 (Egorov 2013). However, all of these subgenera (including *Pincerna* (see Páll-Gergely 2017)) remain inadequately defined as no clear diagnostic characters have ever been identified (Godwin-Austen 1889) and hence will require extensive revision, a task that is beyond the scope of this study and will instead be treated in later studies (B. Páll-Gergely, pers. comm.). Thus, we do not provide a diagnosis for the genus *Alycaeus* here pending a revision of the Alycaeidae and resolution of diagnostic characters useful for supraspecific groups. Instead, consistent with our study scope, we will only provide a description of characters relevant to Peninsular Malaysian *Alycaeus* species.

##### Alycaeus alticola sp. n.

Taxon classificationAnimaliaGastropodaAlycaeidae

http://zoobank.org/E88B3EDB-0DEE-417F-BDEA-613A67ADA99B

**Figure 7.**
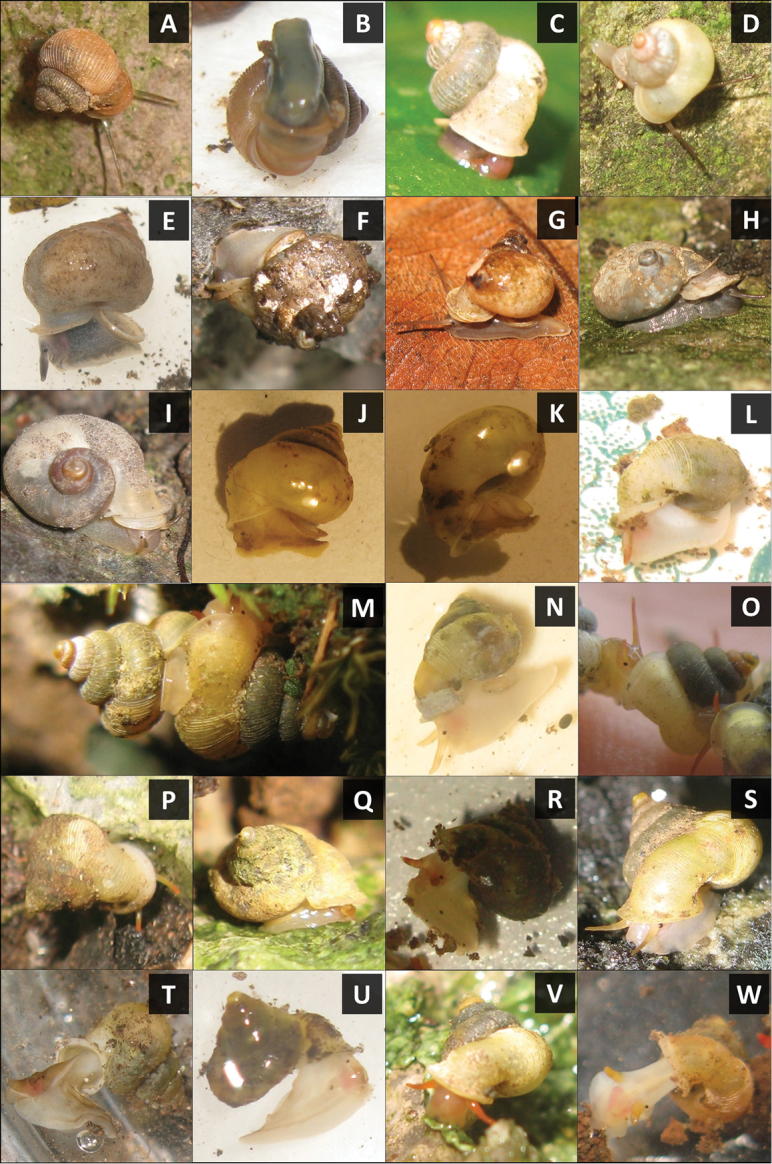
Photographs of 18 living *Alycaeus* species. **A–B** *Alycaeus balingensis* Tomlin, 1948, BOR/MOL 8356 **C** *Alycaeus liratulus* (Preston, 1907), BOR/MOL 8334 **D** *Alycaeus thieroti* Morgan, 1885b, BOR/MOL 6835 **E** *Alycaeus conformis* Fulton, 1902, BOR/MOL 6809 **F** *Alycaeus gibbosulus* Stoliczka, 1872, BOR/MOL 6850 **G** *Alycaeus gibbosulus* Stoliczka, 1872, BOR/MOL 8526 **H** *Alycaeus jousseaumei* Morgan, 1885a, BOR/MOL 8341 **I** *Alycaeus jousseaumei* Morgan, 1885a, BOR/MOL 8336 **J** *Alycaeus alticola* sp. n., BOR/MOL 8398 **K** *Alycaeus charasensis* sp. n., BOR/MOL 8399 **L** *Alycaeus clementsi* sp. n., BOR/MOL 8364 **M** *Alycaeus costacrassa* sp. n., BOR/MOL 6811 **N** *Alycaeus expansus* sp. n., BOR/MOL 6367 **O** *Alycaeus kapayanensis* Morgan, 1885b, BOR/MOL 13005 **P** *Alycaeus kelantanensis* Sykes, 1902, BOR/MOL 8325 **Q** *Alycaeus kelantanensis* Sykes, 1902, BOR/MOL 6200 **R** *Alycaeus kurauensis* sp. n., BOR/MOL 6851 **S** *Alycaeus perakensis* Crosse, 1879a, BOR/MOL 6852 **T** *Alycaeus regalis* sp. n., BOR/MOL 6881 **U** *Alycaeus selangoriensis* sp. n., BOR/MOL 6371 **V–W** *Alycaeus senyumensis* sp. n., BOR/MOL 6249. All photographs by Junn Kitt Foon.

**Figure 8.**
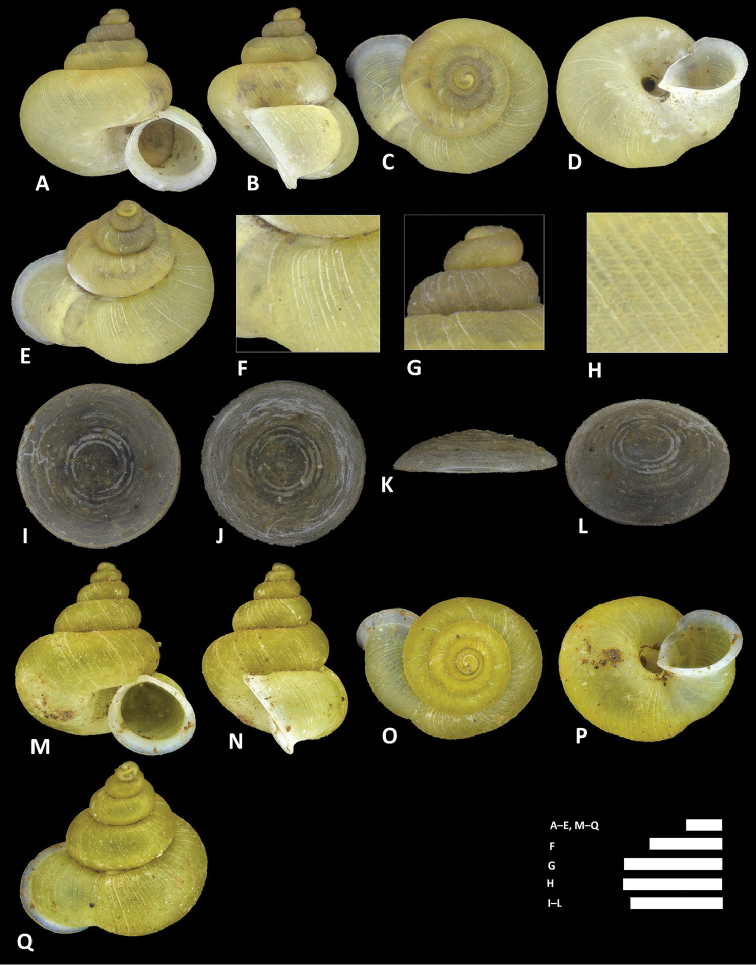
*Alycaeus alticola* sp. n. **A–E** Shell of holotype, BOR/MOL 8398 **F–H** Close up of shell of holotype, BOR/MOL 8398 **I–L** Operculum of paratype, BOR/MOL 8398 **M–Q** Shell of paratype, BOR/MOL 8398. Scale bars: **A–G, I–Q** 1 mm; H 0.5 mm. All photographs by Junn Kitt Foon.

Figures 7J , 8 , 31G

###### Type locality.

PHG 77 Bukit Mengapur, Pahang (3°44'42"N, 102°50'16"E).

###### Type material.

*Holotype.* PHG 77 Bukit Mengapur, Pahang: BOR/MOL 12977. *Paratypes.* PHG 77 Bukit Mengapur, Pahang: BOR/MOL 6888/1, BOR/MOL 8398/26.

###### Other examined materials.

PHG 77 Bukit Mengapur, Pahang: BOR/MOL 8397(11).

###### Etymology.

Latin for “dweller at heights”. Named after the species’ apparent ecological preference for habitats at higher and drier parts of limestone hills.

###### Description.

**Protoconch.** Smooth.

**Shell shape.** Conical. Shell height: 5.08–6.04 mm. Shell width: 5.04–5.87 mm.

**Spire.** Spire height: 1.64–2.23 mm. Spire width: 2.32–2.71 mm. Number of whorls: up to 5. Spire shape: oblong conical. Whorl periphery rounded. Umbilicus open.

**Whorl constriction.** At about 4 ¾ whorls posterior of protoconch.

**Breathing tube.** Length: 0.42–0.58 mm.

**Aperture and peristome.** Aperture circular, moderately expanded. Aperture height: 2.40–2.70 mm. Aperture width: 2.32–2.72 mm. Peristome double, not thickened, slightly notched at suture. Peristome orientation 35–38° oblique with respect to the coiling axis.

**Spiral lines.** Indistinct, regularly spaced. Approximately 22–44 lines per 1 mm.

**Radial ribs running anterior of breathing tube.** Radial ribs pronounced, evenly spaced. Approximately 10–14 ribs per 1 mm.

**Radial ribs running perpendicular to breathing tube.** Radial ribs pronounced, white and thicker than those anterior of breathing tube, evenly spaced. Approximately 9–15 ribs per 1 mm.

**Radial ribs running posterior of breathing tube.** Radial ribs absent immediately posterior of constriction, becoming pronounced and regularly spaced at the middle until prior to aperture. Approximately 7–15 ribs per 1 mm.

**Operculum.** Concave, rounded. Exterior covered with very thin calcareous layer, with indistinct appressed multilamellae. Interior covered with proteinaceous coating, smooth, with indistinct appressed multilamellae and indistinct mamilla.

**Shell colour.** Whorls yellow, fading to white posterior of constriction. Peristome white.

**Living animal.** Body cream-white with fine brown blotches. Head brown. Tentacles brown.

###### Habitat and ecology.

Lives on rock surfaces covered with powdery lichen at higher and drier areas of the limestone hills, often at open places exposed to ambient sunlight. They never appear on wet and mossy sections of limestone rocks and are rarely seen in lower parts of limestone hills that are shaded by forest cover.

###### Distribution range.

Restricted to Bukit Mengapur, Pahang.

###### Differential diagnosis.

*Alycaeus alticola* sp. n. varies in the degree of ultimate whorl expansion within population but has a consistently more expanded and globose whorl compared to *A. costacrassa* sp. n., *A. selangoriensis* sp. n. and *A. kapayanensis*. *Alycaeus alticola* sp. n. shares similar shell shape with *Alycaeus charasensis* sp. n. but differs in the presence of pronounced radial ribs and spiral lines, as well as less expanded ultimate whorl.

###### Discussion.

*A. alticola* sp. n. is among the larger yellow, conical shelled *Alycaeus* in Peninsular Malaysia.

##### Alycaeus altis pirus

Taxon classificationAnimaliaGastropodaAlycaeidae

Möllendorff, 1902

Figures 9 , 31U

Alycaeus perakensis altispirus Möllendorff, 1902: 144–145; Maassen 2001: 23; Tarruella and Domènech 2011: 72.
Alycaeus (Alycaeus) perakensis altispirus : Zilch 1957: 147, plate 6, figure 32.

###### Type locality.

Kelantan.

###### Type material.

*Lectotype*. Kelantan: SMF 109738 (Seen). *Paralectotypes*. Kelantan: SMF 109739/3(Not seen).

###### Description.

**Protoconch.** Smooth.

**Shell shape.** Conical. Shell height: 6.40 mm. Shell width: 6.30 mm.

**Spire.** Spire height: 2.37 mm. Spire width: 2.68 mm. Number of whorls: up to 5 ½. Spire shape: oblong conical. Whorl periphery rounded. Umbilicus open.

**Whorl constriction.** At about 5 ¼ whorls posterior of protoconch.

**Breathing tube.** Length: 0.77 mm.

**Aperture and peristome.** Aperture circular, moderately expanded. Aperture height: 2.87 mm. Aperture width: 3.36 mm. Peristome double, not thickened, notched at suture, upper palatal section folded posteriorly into a wing-like structure. Peristome orientation 36° oblique with respect to the coiling axis.

**Spiral lines.** Absent.

**Radial ribs running anterior of breathing tube.** Radial ribs pronounced, evenly spaced. Approximately 8 ribs per 1 mm.

**Radial ribs running perpendicular to breathing tube.** Radial ribs pronounced, white and thicker than those anterior of breathing tube, evenly spaced. Approximately 16 ribs per 1 mm.

**Radial ribs running posterior of breathing tube.** Radial ribs absent immediately posterior of constriction, becoming pronounced and evenly spaced from the middle section until prior to aperture. Approximately 10 ribs per 1 mm.

**Operculum.** Unknown.

**Shell colour.** Whorls yellow. Peristome white.

**Living animal.** Unknown.

###### Habitat and ecology.

Unknown.

###### Distribution range.

Unknown. Appears to be restricted to Kelantan.

###### Differential diagnosis.

*Alycaeus altispirus* is most similar to *Alycaeus regalis* sp. n. in having a large shell (shell height: 6.40–7.18 mm, shell width: 5.86–6.87 mm) and tall spire (spire height: 2.32–2.90 mm) but differs in the ultimate whorl being more keeled-like, upper palatal section folded posteriorly into a wing-like structure and peristome orientated oblique to the coiling axis. Unlike *Alycaeus perakensis*, *Alycaeus altispirus* is smaller (smaller by about 0.23 mm in shell height, 0.25 mm in shell width) and has a much less expanded ultimate whorl.

###### Discussion.

To date, this species is only known from the four type specimens collected by John Waterstradt (Möllendorff 1902, Zilch 1957) presumably from the same locality as *A. kelantanensis*, on Pulai Princess Cave hill (4°47'38"N, 101°56'31"E), Kelantan (see Discussion under *A. kelantanensis*; Waterstradt 1902, Liew et al. 2014). Yet no recent shells have been found on surveyed limestone hills near the locality (about 5 km from Pulai Princess Cave hill) or elsewhere in Kelantan. At the time of its description, Möllendorff (1902) considered *A. altispirus* a subspecies of *A. perakensis* but did not elaborate why. We disagree with this decision because the two taxa differ markedly from each other in shell shape especially the degree of expansion and shape of the ultimate whorl as well as their very disjunct distribution range. As such, we consider *A. altispirus* a species on its own.

**Figure 9.**
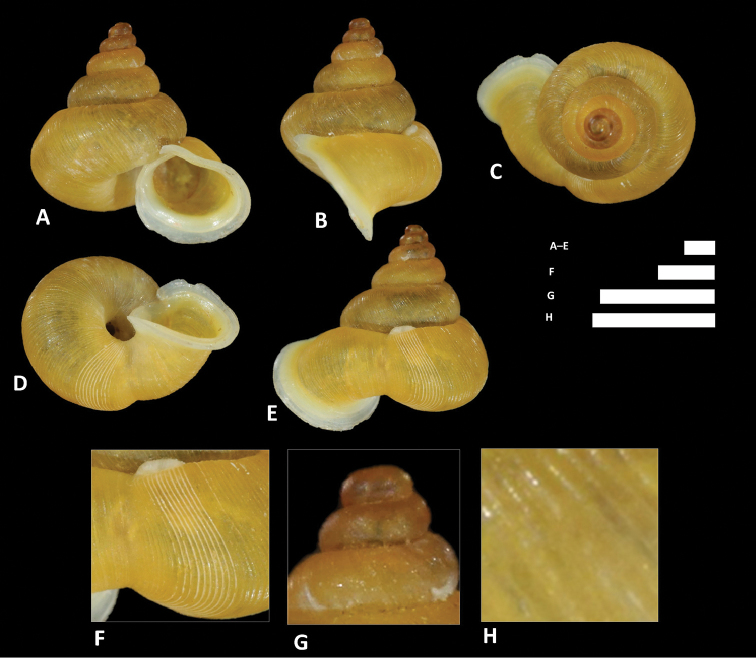
*Alycaeus altispirus* (Möllendorff, 1902) **A–E** Shell of lectotype, SMF 109738 **F–H** Close up of shell of lectotype, SMF 109738. Scale bars: **A–G** 1 mm; **H** 0.5 mm. All photographs by Páll-Gergely Barna, courtesy of R. Janssen, SMF.

##### Alycaeus balingensis

Taxon classificationAnimaliaGastropodaAlycaeidae

Tomlin, 1948

**Figure 10.**
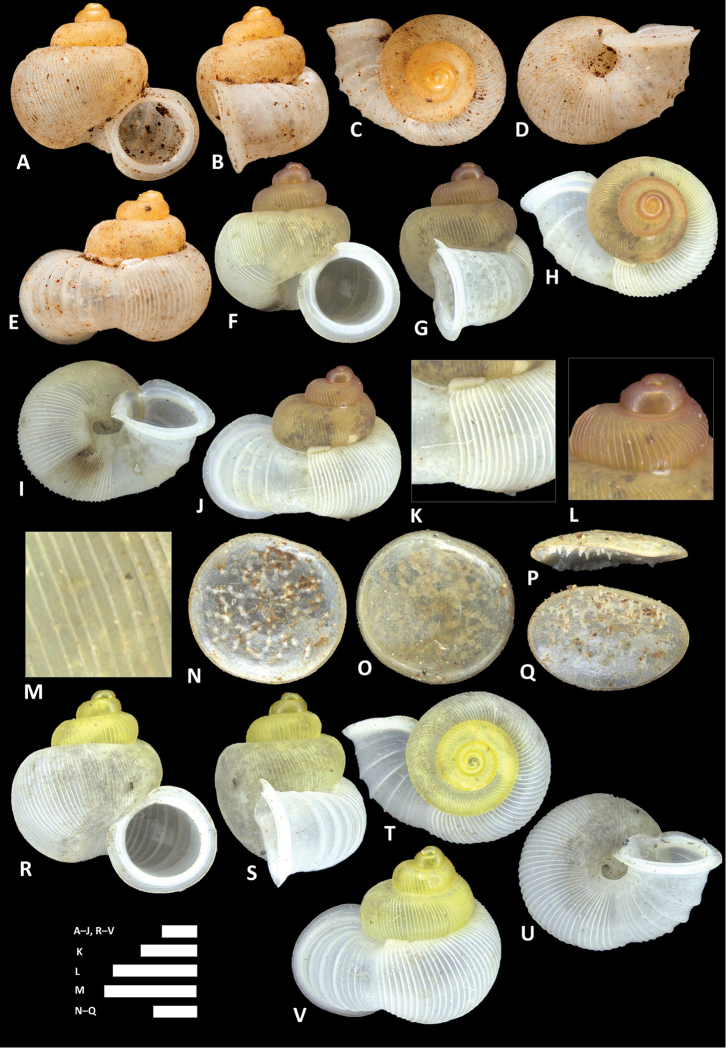
*Alycaeus balingensis* Tomlin, 1948. **A–E** Shell of syntype, NHMUK 1948.10.2.4 **F–J** Shell of BOR/MOL 8357 **K–M** Close up of shell of BOR/MOL 8357 **N–Q** Operculum of BOR/MOL 8357 **R–V** Shell of BOR/MOL 8361. Scale bars: **A–J, R–V, K, L** 1 mm; **M, N–Q** 0.5 mm. Photographs **A–E** by NHM staff (copyright of NHM, reproduced with permission). Photographs **F–V** by Junn Kitt Foon.

Figures 7A–B , 10 , 31A

Alycaeus balingensis Tomlin, 1948: 224–226, plate 11, figure, 3; Maassen 2001: 21; Clements et al. 2008: 2760; Tarruella and Domènech 2011: 72.

###### Type locality.

KDH 01 Bukit Baling, Kedah (5°40'50"N, 100°54'25"E).

###### Type material.

*Syntype.* Bukit Baling, Kedah: NHMUK 1948.10.2.4/1 (1 specimen seen).

###### Other examined materials.

KDH 01 Bukit Baling, Kedah: BOR/MOL 6524/1, BOR/MOL 6853/1, BOR/MOL 8356/13, BOR/MOL 8357/27, ZRC 1997/4, ZRC 1975.2.21.1-85/85. KDH 02 Gunung Pulai, Kedah: BOR/MOL 6856/1, BOR/MOL 8360/18, BOR/MOL 8361/26. Mykarst-171 ‘Tasik Temenggor Hill S2’, Perak: BOR/MOL 6245/1.

###### Description.

**Protoconch.** Smooth.

###### Shell shape and size.

Globose. Shell height: 4.75–5.87 mm. Shell width: 5.11–6.00 mm.

**Spire.** Spire height: 1.23–2.16 mm. Spire width: 2.09–2.54 mm. Number of whorls: up to 4 ⅛. Spire shape: oblong conical. Whorl periphery rounded. Umbilicus open.

**Whorl constriction.** At about 3 ¾ whorls posterior of protoconch.

**Breathing tube.** Length: 0.70–0.82 mm.

**Aperture and peristome.** Aperture circular, moderately expanded. Aperture height: 2.60–3.13 mm. Aperture width: 2.69–3.22 mm. Peristome double, not thickened, slightly notched at suture. No interspace. Peristome orientation 4–8° oblique with respect to the coiling axis.

**Spiral lines.** Absent.

**Radial ribs running anterior of breathing tube.** Radial ribs pronounced, evenly spaced. Approximately 9–16 ribs per 1 mm.

**Radial ribs running perpendicular to breathing tube.** Radial ribs pronounced, evenly spaced. Approximately 7–10 ribs per 1 mm.

**Radial ribs running posterior of breathing tube.** Radial ribs initially indistinct and dense, but increasingly becomes pronounced and evenly-spaced towards the aperture. Approximately 2–4 ribs per 1 mm.

**Operculum.** Concave, rounded. Exterior covered by calcareous layer, with short calcareous spikes. Interior covered by proteinaceous layer, smooth.

**Shell colour.** Red, yellow or white at apical whorls. All colours fade to white towards ultimate whorl.

**Living animal.** Body greenish-black. Head greenish-black. Tentacle green with brown tips.

###### Habitat and ecology.

Lives in crevices on wet limestone boulders and shrubs close to the ground. In forested areas of limestone hills.

**Distribution.** Restricted to northern Perak (mykarst-171) and northeastern Kedah (Bukit Baling and Gunung Pulai).

###### Differential diagnosis.

*Alycaeus balingensis* shares similar shell shape (globose whorls) with *Alycaeus liratulus* and *Alycaeus thieroti* but differs in having whorls with denser and more pronounced radial ribs, absence of spiral lines, operculum exterior with short spikes and greenish-black body.

###### Discussion.

*Alycaeus balingensis* is listed as Critically Endangered in the IUCN Red List as it was known only from the actively quarried Bukit Baling at the time of assessment (Clements 2009). Given that new populations have now been discovered in two other localities in Kedah and Perak, the conservation status of *A. balingensis* should be revised.

##### Alycaeus carinata

Taxon classificationAnimaliaGastropodaAlycaeidae

Maassen, 2006

Figures 11 , 31Q

Alycaeus carinata Maassen, 2006: 137–138, figures 10–13; Tarruella and Domènech 2011: 72.

###### Type locality.

PHG 74 Bukit Sagu, Pahang (3°58'54"N, 103°08'39"E).

###### Type material.

*Holotype.* PHG 74 Bukit Sagu, Pahang: RMNH 104428 (Not seen). *Paratypes.* PHG 74 Bukit Sagu, Pahang: RMNH 104427/2 (1 specimen seen), HW /58 (Not seen), ANSP /2 (Not seen), HW /200 (Not seen). PHG 75 Bukit Tenggek, Pahang: RMNH 104426/2 (Not seen), HW /37 (Not seen).

###### Description.

**Protoconch.** Smooth.

**Shell shape.** Almost flat, conical. Shell height: 2.2–3.0 mm. Shell width: 4.0–4.6 mm. Shell measurements derived from Maassen (2006) and re-examination of one paratype (RMNH 104427).

**Spire.** Spire height: 3.0 mm. Spire width: 4.5 mm. Number of whorls: up to 4. Spire shape: oblong conical. Whorl periphery strongly keeled except posterior of the constriction, where it becomes rounded. Umbilicus open.

**Whorl constriction.** At about 3 ¾ whorls posterior of protoconch.

**Breathing tube.** Length: 0.3 mm.

**Aperture and peristome.** Aperture circular, moderately expanded. Aperture height: 2.2 mm. Aperture width: 2.0 mm. Peristome double, not thickened, winged at suture. Interspace narrow. Peristome orientation is 30–55° oblique with respect to the coiling axis.

**Spiral lines.** Indistinct. Regularly spaced. Approximately 25 lines per 1 mm.

**Radial ribs running anterior of breathing tube.** Radial ribs indistinct to absent, only unevenly spaced radial growth lines present.

**Radial ribs running perpendicular to breathing tube.** Radial ribs pronounced, evenly spaced. Approximately 13 ribs per 1 mm.

**Radial ribs running posterior of breathing tube.** Radial ribs indistinct to absent, only unevenly spaced radial growth lines present.

**Operculum.** Concave, rounded. Exterior and interior composition and sculpture not examined. Maassen (2006) considered the operculum entirely proteinaceous.

**Shell colour.** Whorls yellow to brown. Peristome white.

**Living animal.** Unknown.

###### Habitat and ecology.

Lives on limestone walls in a large, collapsed cave (Maassen 2006). Probably prefers very moist environments only (Maassen 2006).

###### Distribution range.

Restricted to Bukit Sagu and Bukit Tenggek, Pahang.

###### Differential diagnosis.

*Alycaeus carinata* is distinguished from all other Peninsular Malaysian *Alycaeus* species by its strongly keeled periphery, relatively low spire, very expanded ultimate whorls and very oblique peristome orientation with respect to the coiling axis (Maassen 2006).

###### Discussion.

Interestingly, no *A. carinata* was found during the 1950s University of Malaya, Singapore expedition to the type locality although congeners *Alycaeus charasensis* sp. n. and *Alycaeus expansus* sp. n. were obtained (ZRC collection lots). This suggests *A. carinata* may be restricted to upper sections of limestone hills that became accessible only during quarrying in the 1990s. However, viable habitats at Bukit Sagu and Bukit Tenggek are now very likely lost to quarrying (Schilthuizen and Clements 2008, Liew et al. 2014).

**Figure 11.**
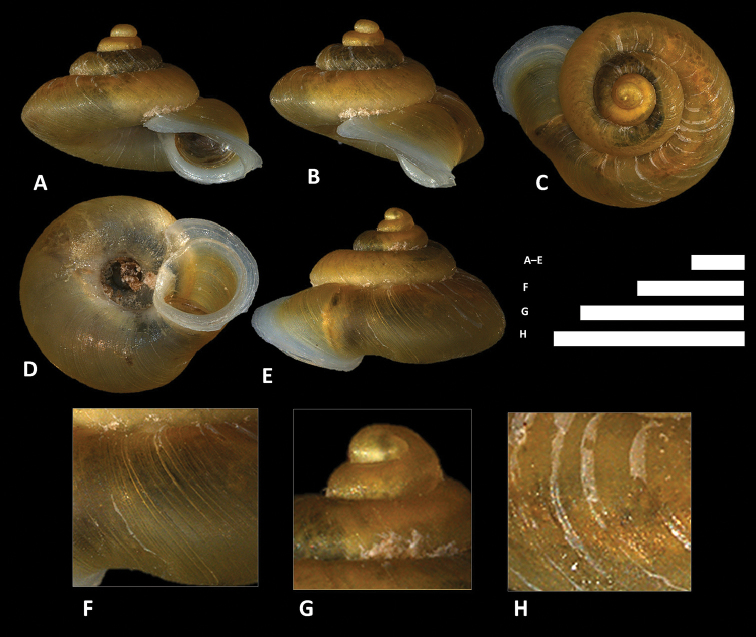
*Alycaeus carinata* Maassen, 2006 **A–E** Shell of paratype, RMNH 104427 **F–H** Close up of shell of paratype, RMNH 104427. Scale bars: **A–G** 1 mm; **H** 0.5 mm. All photographs by Thor Seng Liew.

##### Alycaeus charasensis sp. n.

Taxon classificationAnimaliaGastropodaAlycaeidae

http://zoobank.org/05839D39-E09B-4635-9254-BBF285A60A90

**Figure 12.**
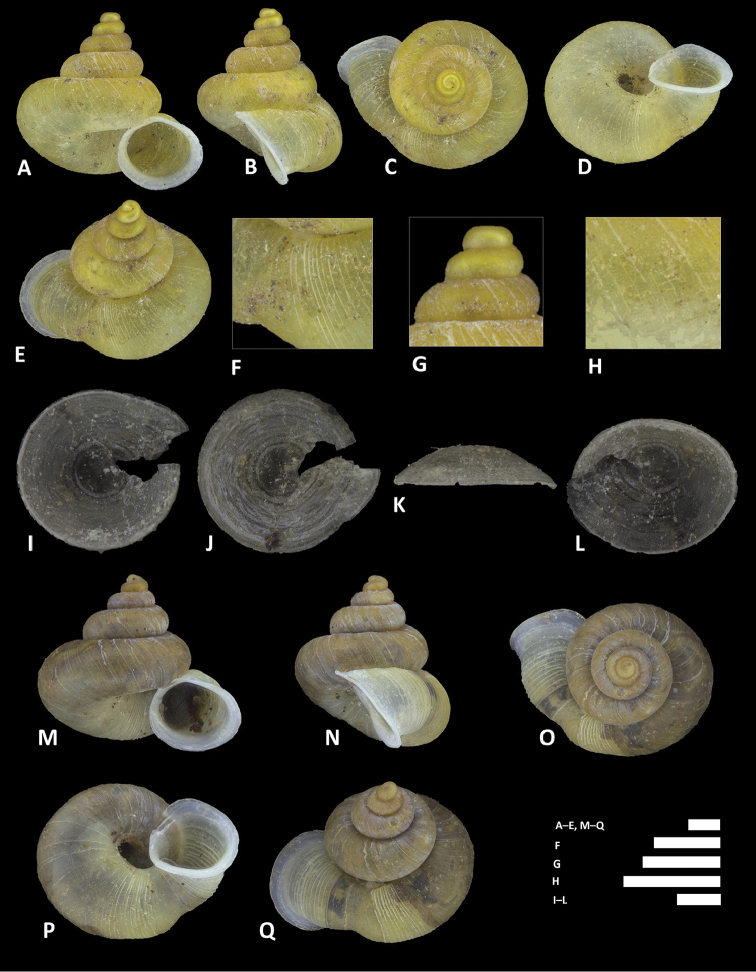
*Alycaeus charasensis* sp. n. **A–E** Shell of holotype, BOR/MOL 8400 **F–H** Close up of shell of holotype, BOR/MOL 8400 **I–L** Operculum of paratype, BOR/MOL 6891 **M–Q** Shell of paratype, BOR/MOL 267. Scale bars: **A–G, M–Q, H** 1 mm; **I–L** 0.5 mm. All photographs by Junn Kitt Foon.

Figures 7K , 12 , 31H

###### Type locality.

PHG 73 Bukit Charas, Pahang (3°54'35"N, 103°08'48"E).

###### Type material.

*Holotype.* PHG 73 Bukit Charas, Pahang: BOR/MOL 12981. *Paratypes.* PHG 75 Bukit Tenggek, Pahang: BOR/MOL 264/2. PHG 73 Bukit Charas, Pahang: BOR/MOL 267/3, BOR/MOL 6891/1.

###### Other examined materials.

PHG 73 Bukit Charas, Pahang: BOR/MOL 8399/14, BOR/MOL 8401/26, BOR/MOL 8402/166, BOR/MOL 12982/120, ZRC 1975.2.21.431-434/4, ZRC 1975.2.22.444-677/234, ZRC 1975.2.24.132-153/22, ZRC 1975.2.24.1-43/43, ZRC 1975.2.24.93-131/38, ZRC 1975.2.24.83-89/6. PHG 75 Bukit Tenggek, Pahang: ZRC 1975.2.24.44-/>1.

###### Etymology.

Subspecific epithet named after the type locality, Bukit Charas.

###### Description.

**Protoconch.** Smooth.

**Shell shape.** Conical. Shell height: 5.09–5.82 mm. Shell width: 5.23–6.62 mm.

**Spire.** Spire height: 1.57–2.11 mm. Spire width: 2.38–2.77 mm. Number of whorls: up to 5. Spire shape: oblong conical. Whorl periphery rounded. Umbilicus open.

**Whorl constriction.** At about 4 ⅞ whorls posterior of protoconch.

**Breathing tube.** Length: 0.46–0.57 mm.

**Aperture and peristome.** Aperture circular, moderately expanded. Aperture height: 2.58–2.90 mm. Aperture width: 2.47–2.84 mm. Peristome double, not thickened, slightly notched at suture. Peristome orientation 34–43° oblique with respect to the coiling axis.

**Spiral lines.** Indistinct, regularly spaced. Approximately 16–60 lines per 1 mm.

**Radial ribs running anterior of breathing tube.** Radial ribs indistinct, unevenly spaced. They are sometimes absent and are replaced by evenly spaced radial growth lines.

**Radial ribs running perpendicular to breathing tube.** Radial ribs pronounced, white and thick, evenly spaced. Approximately 8–15 ribs per 1 mm.

**Radial ribs running posterior of breathing tube.** Radial ribs absent immediately posterior of constriction, becoming pronounced and evenly spaced at the middle until prior to aperture. Approximately 7–19 ribs per 1 mm.

**Operculum.** Concave, rounded. Exterior covered with thin calcareous layer, with indistinct appressed multilamellae. Interior covered with proteinaceous coating, smooth, with indistinct appressed multilamellae.

**Shell colour.** Whorls yellow, fading to white posterior of constriction. Peristome white.

**Living animal.** Body cream-white with fine brown blotches. Head brown. Tentacles brown.

###### Habitat and ecology.

Lives on rock surfaces covered with powdery lichen at higher and drier areas of the limestone hills, often at open places exposed to ambient sunlight. They never appear on wet and mossy sections of limestone rocks and are not found in lower parts of limestone hills that are shaded by forest cover.

###### Distribution range.

Restricted to Bukit Charas and Bukit Tenggek, eastern Pahang.

###### Differential diagnosis.

*Alycaeus charasensis* sp. n. shares similar variability in shell shape with *Alycaeus alticola* sp. n. but consistently differs in having a larger shell, wider ultimate whorl, widely spaced spiral lines and uneven, widely spaced radial growth lines in place of radial ribs. *Alycaeus charasensis* sp. n. is distinguished from other tall spired, conical yellow shelled *Alycaeus* in Peninsular Malaysia by its lack of distinct radial ribs.

###### Discussion.

*A. charasensis* sp. n. is among the larger yellow, conical shelled *Alycaeus* in Peninsular Malaysia.

##### Alycaeus clementsi sp. n.

Taxon classificationAnimaliaGastropodaAlycaeidae

http://zoobank.org/713345B0-4FB9-4AFF-9DD3-9A3AF78B19B1

**Figure 13.**
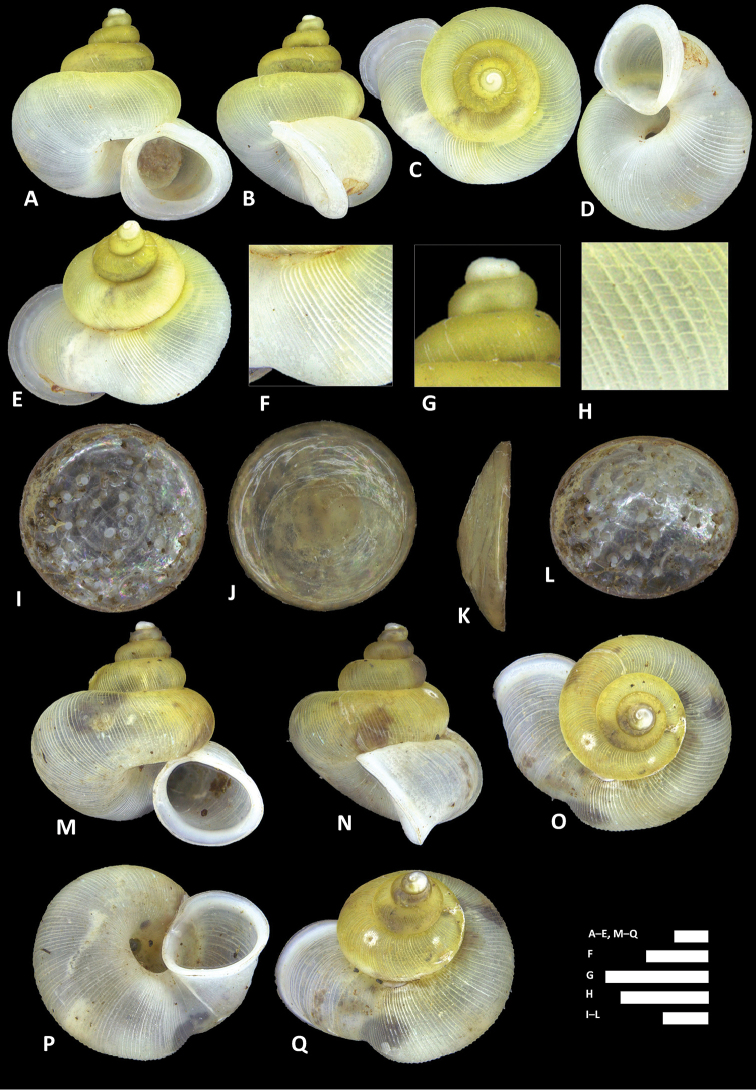
*Alycaeus clementsi* sp. n. **A–E** Shell of holotype, BOR/MOL 12970 **F–H** Close up of shell of holotype, BOR/MOL 12970 **I–L** Operculum of paratype, BOR/MOL 6865 **M–Q** Shell of paratype, BOR/MOL 8377. Scale bars: **A–G, M–Q** 1 mm; **H, I–L** 0.5 mm. All photographs by Junn Kitt Foon.

Figures 7L , 13 , 31J

Alycaeus perakensis : Benthem Jutting 1960a: 13. [not Alycaeus perakensis Crosse, 1879]

###### Type locality.

Gua Kelam, PRS 64 Wang Ulu, Perlis (6°38'41"N, 100°12'09"E).

###### Type material.

*Holotype.* Gua Kelam, PRS 64 Wang Ulu, Perlis: BOR/MOL 12970. *Paratypes.* Gua Kelam, PRS 64 Wang Ulu, Perlis: BOR/MOL 276/3, BOR/MOL 6865/1, BOR/MOL 8371/2. Bukit Ayer, PRS 64 Wang Ulu, Perlis: BOR/MOL 8365/2. Hill 9 km along the road from Kangar to Kaki Bukit, Perlis: BOR/MOL 270/1. KDH 10 Bukit Kodiang, Kedah: BOR/MOL 12969/1, BOR/MOL 8377/62. KDH 04 Gunung Keriang, Kedah: BOR/MOL 8407/7, BOR/MOL 12968/1. Limestone hill north of Sungai Ewa, Pulau Langkawi, Kedah: BOR/MOL 265/3.

###### Other examined materials.

Gua Kelam, PRS 64 Wang Ulu, Perlis: BOR/MOL 6194/4, BOR/MOL 6867/1, BOR/MOL 8370/11, BOR/MOL 8373/3, ZRC 1997.21/2. Bukit Ayer, PRS 64 Wang Ulu, Perlis: BOR/MOL 6251/6, BOR/MOL 6861/1, BOR/MOL 8364/10. PRS 40 Bukit Merbok, Perlis: BOR/MOL 7071/1. KDH 04 Gunung Keriang, Kedah: BOR/MOL 6246/8, BOR/MOL 6252/20, BOR/MOL 6864/1, BOR/MOL 8369/40, BOR/MOL 8378/1, BOR/MOL 12971/1, BOR/MOL 6191/10, ZRC 1997.19/2. ZRC 1997.20/2, ZRC 1975.2.22.67-98/32. KDH 10 Bukit Kodiang, Kedah: BOR/MOL 6872/1.

###### Etymology.

Named after Gopalasamy Reuben Clements, in recognition of his contribution to the study of land snail biogeography on Peninsular Malaysian karsts.

###### Description.

**Protoconch.** Smooth.

**Shell shape.** Conical. Shell height: 5.65–6.98 mm. Shell width: 5.71–7.32 mm.

**Spire.** Spire height: 1.87–2.62 mm. Spire width: 2.39–3.32 mm. Number of whorls: up to 5 ⅛. Spire shape: oblong conical. Whorl periphery rounded. Umbilicus open.

**Whorl constriction.** At about 4 ¾whorls posterior of protoconch.

**Breathing tube.** Length: 0.59–0.90 mm.

**Aperture and peristome.** Aperture circular, moderately expanded. Aperture height: 2.95–3.88 mm. Aperture width: 2.83–3.49 mm. Peristome double, thickened, notched at suture. Peristome orientation 25–45° oblique with respect to the coiling axis.

**Spiral lines.** Distinct. Regularly spaced. Approximately 11–34 lines per 1 mm.

**Radial ribs running anterior of breathing tube.** Radial ribs pronounced, evenly spaced. Approximately 9–16 lines per 1 mm.

**Radial ribs running perpendicular to breathing tube.** Radial ribs pronounced, white and thicker than those anterior of breathing tube, evenly spaced. Approximately 11–16 radial ribs per 1 mm

**Radial ribs running posterior of breathing tube.** Radial ribs pronounced, unevenly spaced immediately posterior of constriction, becoming pronounced and evenly spaced from the middle section until prior to aperture. Approximately 11–16 ribs per 1 mm.

**Operculum.** Concave, rounded. Exterior covered with thin calcareous layer, surface finely granulated with a scattered arrangement of calcareous blunt spikes. Interior covered with proteinaceous coating, smooth, mamillated.

**Shell colour.** Yellow at the first 4 whorls. Fades to white towards aperture.

**Living animal.** Body cream-white. Head pink. Tentacles cream-white, tips pink.

###### Habitat and ecology.

Lives on wet limestone rock walls and crevices. In shady forests on limestone hills.

###### Distribution range.

In Perlis, retricted to limestone hills of the Nakawan Range. In Kedah, restricted to limestone hills of the Langkawi archipelago, Kodiang and Gunung Keriang.

###### Differential diagnosis.

*Alycaeus clementsi* sp. n. is similar to *A. kelantanensis* in its shell colouration. Despite shell size of *A. clementsi* sp. n. being variable within populations across its range, *A. clementsi* sp. n. is distinguished from *A. kelantanensis* by its consistently large shell (larger by about 2.13 mm in shell height, 2.32 mm in shell width), thick expanded peristome, spiky operculum exterior as well as the animal colouration.

###### Discussion.

Historically, *Alycaeus clementsi* sp. n. shells from Kaki Bukit, Perlis were misidentified as *A. perakensis* in Benthem Jutting (1960a). Shells of *A. clementsi* sp. n. in Perlis usually have thicker peristome compared to shells from Kedah albeit all other diagnostic characters remain identical. Shell size of *A. clementsi* sp. n. is variable within populations across its range and does not appear to have any geographical pattern. This species may occur in Thailand at areas adjacent to Perlis and northern Kedah.

##### Alycaeus conformis

Taxon classificationAnimaliaGastropodaAlycaeidae

Fulton, 1902

**Figure 14.**
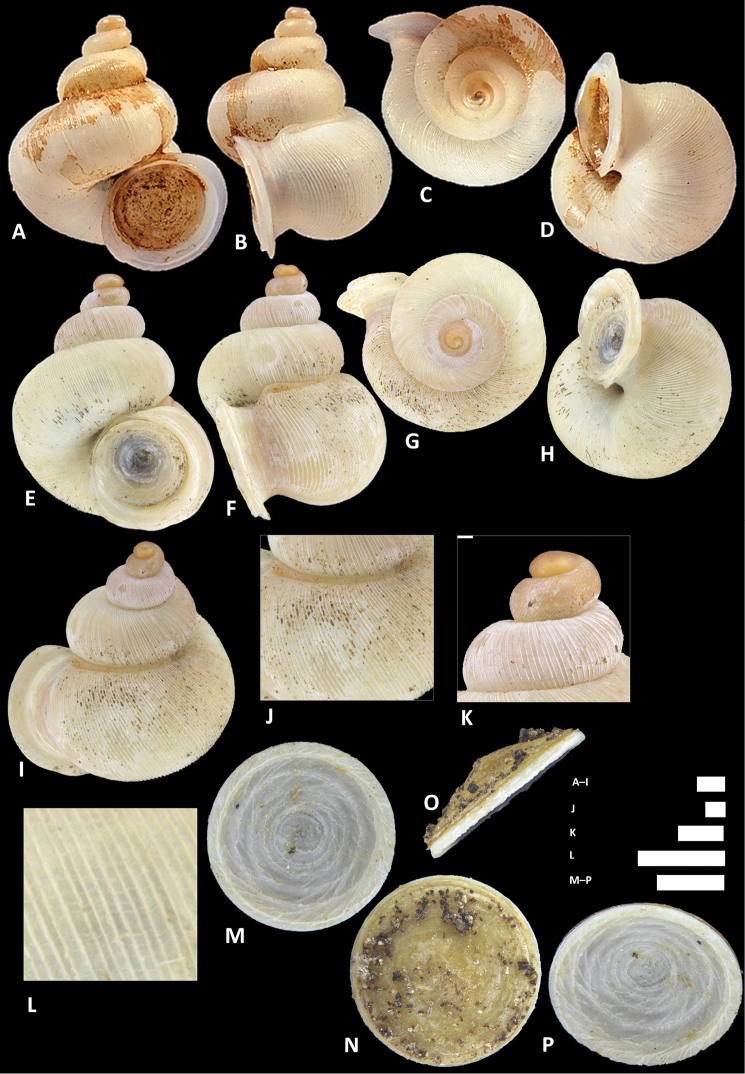
*Alycaeus conformis* Fulton, 1902. **A–D** Shell of syntype, NHMUK 1902.5.28.22-23 **E–I** Shell of BOR/MOL 6526 **J–L** Close up of shell of BOR/MOL 6526 **M–P** Operculum of BOR/MOL 12986. Scale bars: **A–P** 1 mm. Photographs **A–D** by Harold Taylor (copyright of NHM, reproduced with permission). Photographs **E–P** by Junn Kitt Foon.

Figures 7E , 14 , 31D

Alycaeus conformis Fulton, 1902: 68–69; Sykes 1903: 198; Laidlaw 1928: 34; Laidlaw 1932: 36; Venmans 1956: 81, figure 1; Robertson et al. 1987: 2; Chan 1997: 37; Tarruella and Domènech 2011: 72; Páll-Gergely et al. 2016: 20160151; Foon et al. 2017: 10, figure 4B.
Alycaeus confirmis [sic]: Maassen 2001: 21.
Alycaeus coniformis [sic]: Tarruella and Domènech 2011: 72, figure. 1B.

###### Type locality.

Perak.

###### Type material.

*Syntypes.* Perak: ANSP 84690/2 (Seen), NHMUK 1902.5.28.22-23/2 (Seen).

###### Other examined materials.

KTN 01 Gunung Reng, Kelantan: BOR/MOL 6836/2. KTN 66 Batu Tongkat, Kelantan: ZRC 1975.2.21.88/1. Lojing, Kelantan: BOR/MOL 6526/1. PHG 03 Gunung Jebak Puyuh, Pahang: BOR/MOL 6884/1. PHG 01 Kota Gelanggi, Pahang: BOR/MOL 6887/1, BOR/MOL 8396/2. Mykarst-122 Bukit Merah, Pahang: BOR/MOL 12986/1. PHG 02 Gunung Senyum, Pahang: BOR/MOL 12987/1. PHG 05 Bukit Cintamanis, Pahang: BOR/MOL 6893/1, BOR/MOL 8403/2, BOR/MOL 8404/2. PHG 72 Bukit Panching, Pahang: ANSP 439103/1. Perak: ZRC 1975.2.21.86/1, RMNH 153478/2. PRK 23 Gunung Rapat, Perak: BOR/MOL 10057/1. SGR 02 Bukit Takun, Selangor: BOR/MOL 6809/1, BOR/MOL 8316/2. SGR 01 Batu Caves, Selangor: ZRC 1975.2.21.87/1, ZRC 1990.10274-10288/14. Endau-Kluang, Johor: BOR/MOL 6458/1, BOR/MOL 6459/1.

###### Description.

**Protoconch.** Smooth at first whorl, becomes grooved in the second whorl. Grooves arranged parallel to each other and tilted anteriorly at 45° to the coiling axis.

**Shell shape.** Conical. Shell height: 8.77–10.19 mm. Shell width: 7.16–8.19 mm.

**Spire.** Spire height: 2.37–3.21 mm. Spire width: 3.24–3.66 mm. Number of whorls: up to 4 ⅜. Spire shape: oblong conical. Whorl periphery rounded. Umbilicus open.

**Whorl constriction.** At about 4 ¼ whorls posterior of protoconch.

**Breathing tube.** Length: 4.70–5.76 mm.

**Aperture and peristome.** Aperture circular, moderately expanded. Aperture height: 4.11–5.14 mm. Aperture width: 4.27–4.93 mm. Peristome double, thickened, slightly notched at suture. Interspace narrow. Peristome orientation 10–16° oblique with respect to the coiling axis.

**Spiral lines.** Indistinct, regularly spaced. Approximately 11–59 lines per 1 mm.

**Radial ribs running anterior of breathing tube.** Radial ribs pronounced, evenly spaced. Approximately 10–17 ribs per 1 mm.

**Radial ribs running perpendicular to breathing tube.** Radial ribs more pronounced and thicker than those anterior of breathing tube, evenly spaced. Approximately 9–13 ribs per 1 mm.

**Radial ribs running posterior of breathing tube.** Ribs absent. Only radial growth lines.

**Operculum.** Concave, conical. Exterior covered by thick calcareous layer. Exterior has radially spiral lamellae of 6 revolutions. Interior covered by proteinaceous layer, mamillated.

**Shell colour.** First 3 whorls usually red or pink. All colours fade to white towards ultimate whorl.

**Living animal.** Body grey. Head grey. Tentacles dark grey to black.

###### Habitat and ecology.

Lives under large rotten logs and on leaf litter. In wet, shady forests in both limestone and non-limestone areas.

**Distribution.** Localised in Perak but widespread in Kelantan, Pahang, Selangor and Johor. Elsewhere, in Phuket, southern Thailand (Páll-Gergely et al. 2016).

**Differential diagnosis** *Alycaeus conformis* is most similar to *Alycaeus gibbosulus* in shell shape but differs in having a less expanded penultimate whorl, finer radial ribs anterior of breathing tube, shorter constriction whorl, shorter breathing tube, less oblique peristome and multilamellated operculum exterior. The animal body is dark grey in *A. conformis* compared to brown in *A. gibbosulus*.

###### Discussion.

Fulton (1902) stated the type locality of *A. conformis* as Perak without further specifying the location. Perak is a large state in western Peninsular Malaysia. During recent intensive sampling across limestone hills in Perak, *Alycaeus conformis* was only discovered at one locality, Gunung Rapat. *Alycaeus conformis* has also been reported from Kelantan (Sykes 1903, Laidlaw 1928), Selangor (Laidlaw 1932, Venmans 1956), Pahang and Johor. Venmans (1956) described the radular morphology of *A. conformis* from Batu Caves, Selangor. Páll-Gergely et al. (2016) described the microstructures associated with the breathing tube of *A. conformis* from Phuket Island, Thailand. The shells of *A. conformis* are often coated in a layer of organic matter when alive (Figure 5E).

##### Alycaeus costacrassa sp. n.

Taxon classificationAnimaliaGastropodaAlycaeidae

http://zoobank.org/A08F755C-2292-4CA7-92AD-75D78ED1B4B1

**Figure 15.**
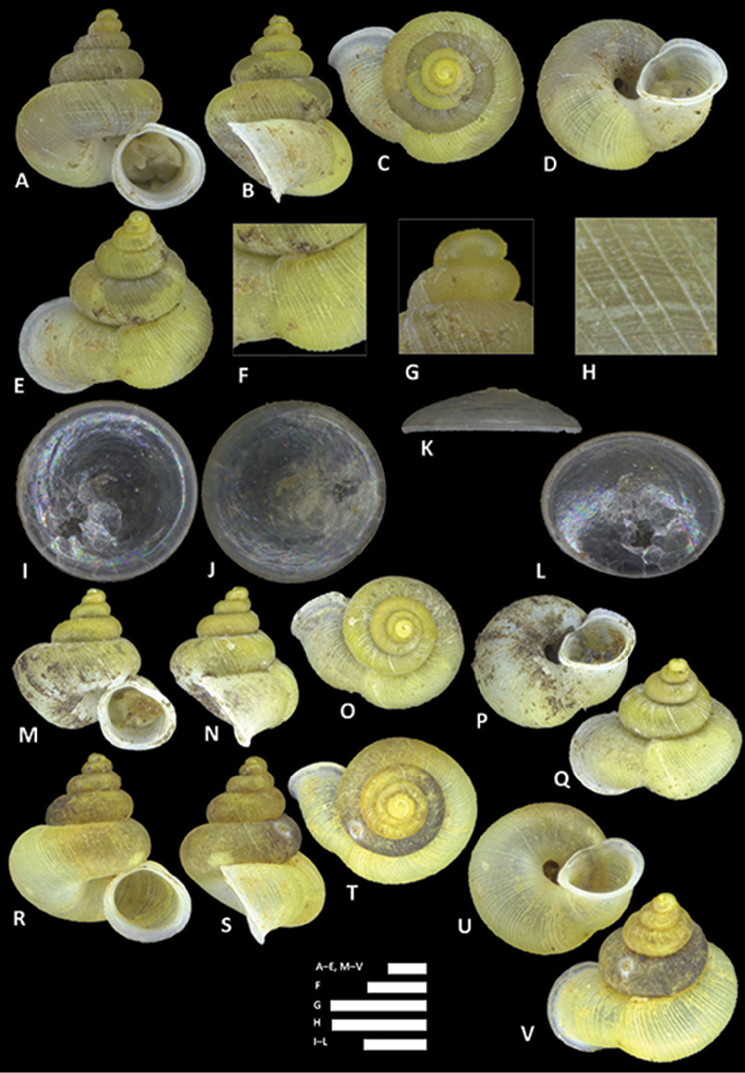
*Alycaeus costacrassa* sp. n. **A–E** Shell of holotype, BOR/MOL 6811 **F–H** Close up of shell of holotype, BOR/MOL 6811 **I–L** Operculum of holotype, BOR/MOL 6811 **M–Q** Shell of BOR/MOL 6818 **R–V** Shell of BOR/MOL 12992. Scale bars: **A–G** 1 mm; **H, I–L** 0.5 mm. All photographs by Junn Kitt Foon.

Figures 7M , 15 , 31N

Alycaeus perakensis var. minor nomen nudum, Clements et al. 2008: 2760.

###### Type locality.

Mykarst-065 Batu Balong, Pahang (3°42'41"N, 101°51'25"E).

###### Type material.

*Holotype.* Mykarst-065 Batu Balong, Pahang: BOR/MOL 12995. *Paratypes.* Mykarst-065 Batu Balong, Pahang: BOR/MOL 8320/2, BOR/MOL 6811/4, BOR/MOL 8319/6.

###### Other examined materials.

KTN 06 Gua Ikan, Kelantan: BOR/MOL 6631/1. KTN 148 unnamed hill, Kelantan: BOR/MOL 12993/1. KTN 95 Gua Panjang, Kelantan: BOR/MOL 12994/12. PHG 15 Gua Bama, Pahang: BOR/MOL 243/1, BOR/MOL 6235/4, BOR/MOL 6818/2, BOR/MOL 8323/2, BOR/MOL 8324/6. Mykarst-169 Gua Gajah, Pahang: BOR/MOL 6250/5, BOR/MOL 12992/1. PHG 03 Gunung Jebak Puyuh, Pahang: BOR/MOL 6628/3, BOR/MOL 6882/1. PHG 05 Bukit Cintamanis, Pahang: BOR/MOL 6634/2, BOR/MOL 6892/2. PHG 01 Kota Gelanggi, Pahang: BOR/MOL 6635/1, ZRC 1975.2.24.356-372/7, ZRC 1975.2.24.373-402/30. PHG 16 Gua Sai, Pahang: BOR/MOL 6637/80, BOR/MOL 6638/30, ZRC 1975.2.21.557-564/10. Ulu Keniyam Kechil, Pahang: ZRC 1975.2.22.660-692/32.

###### Etymology.

In Latin – costa crassa, meaning thick ribbed. Named after the species’ diagnostic widely spaced, pronounced radial ribbing of the whorls.

###### Description.

**Protoconch.** Smooth.

**Shell shape.** Conical. Shell height: 3.47–6.37 mm. Shell width: 3.42–6.12 mm.

**Spire.** Spire height: 1.06–2.43 mm. Spire width: 1.53–2.79 mm. Number of whorls: up to 5 ¼. Spire shape: oblong conical. Whorl periphery rounded. Umbilicus open.

**Whorl constriction.** At about 5 whorls posterior of protoconch.

**Breathing tube.** Length: 0.30–0.67 mm.

**Aperture and peristome.** Aperture circular, moderately expanded. Aperture height: 1.79–2.89 mm. Aperture width: 1.62–2.84 mm. Peristome double, not thickened, winged at suture. Peristome orientation 26–39° oblique with respect to the coiling axis.

**Spiral lines.** Distinct. Regularly spaced. Approximately 19–52 lines per 1 mm.

**Radial ribs running anterior of breathing tube.** Radial ribs pronounced, evenly spaced. Approximately 9–18 ribs per 1 mm.

**Radial ribs running perpendicular to breathing tube.** Radial ribs pronounced, evenly spaced. Approximately 11–17 ribs per 1 mm.

**Radial ribs running posterior of breathing tube.** Radial ribs pronounced and ueneven immediately posterior of constriction, becoming pronounced and evenly spaced towards the aperture. Approximately 10–21 ribs per 1 mm.

**Operculum.** Concave, rounded. Exterior covered with thick but flaky calcareous layer, surface finely granulated. Interior proteinaceous layered, smooth, with indistinct mamilla.

**Shell colour.** Whorls yellow, fading to white posterior of constriction. Peristome white.

**Living animal.** Body cream-white. Head pinkish brown. Tentacles reddish-brown.

###### Habitat and ecology.

Lives on wet limestone walls covered with mosses. Occassionally found in crevices, on lower parts of tree trunks. In shady forests on limestone hills.

###### Distribution range.

Widespread from southern Kelantan to central Pahang.

###### Differential diagnosis.

*Alycaeus costacrassa* sp. n. most resembles *A. selangoriensis* sp. n. in its thick operculum, pronounced radial ribbing and spiral lines but has a narrower ultimate whorl and a more circular peristome without posterior folding at the upper palatal section. *Alycaeus costacrassa* sp. n. also resembles *A. kapayanensis* in shell shape but differs in its consistently more pronounced radial ribs as well as operculum with thicker calcareous exterior.

###### Discussion.

Shells of *A. costacrassa* sp. n. are consistent in their diagnostic characters but vary considerably in shell size and whorl convexity across its range. Populations in Gua Sai and Gua Bama have the smallest shells (Shell height: 3.47–4.46 mm, Shell width: 3.42–4.27 mm, Figure 15M–Q) with less rounded whorls. Gua Gajah shells are intermediate in size (Shell height: 4.85 mm, Shell width: 4.67 mm, Figure 15R–V) with rounded whorls. Batu Balong and Gua Panjang have larger shells (Shell height: 4.99–5.94 mm, Shell width: 4.66–5.58 mm, Figure 15A–E) with rounded whorls. It is possible that *A. costacrassa* sp. n. may contain several cryptic species pending availability of molecular data for investigation.

##### Alycaeus expansus sp. n.

Taxon classificationAnimaliaGastropodaAlycaeidae

http://zoobank.org/E2003BE4-BAD9-42B9-A89D-40ABB6EACED1

**Figure 16.**
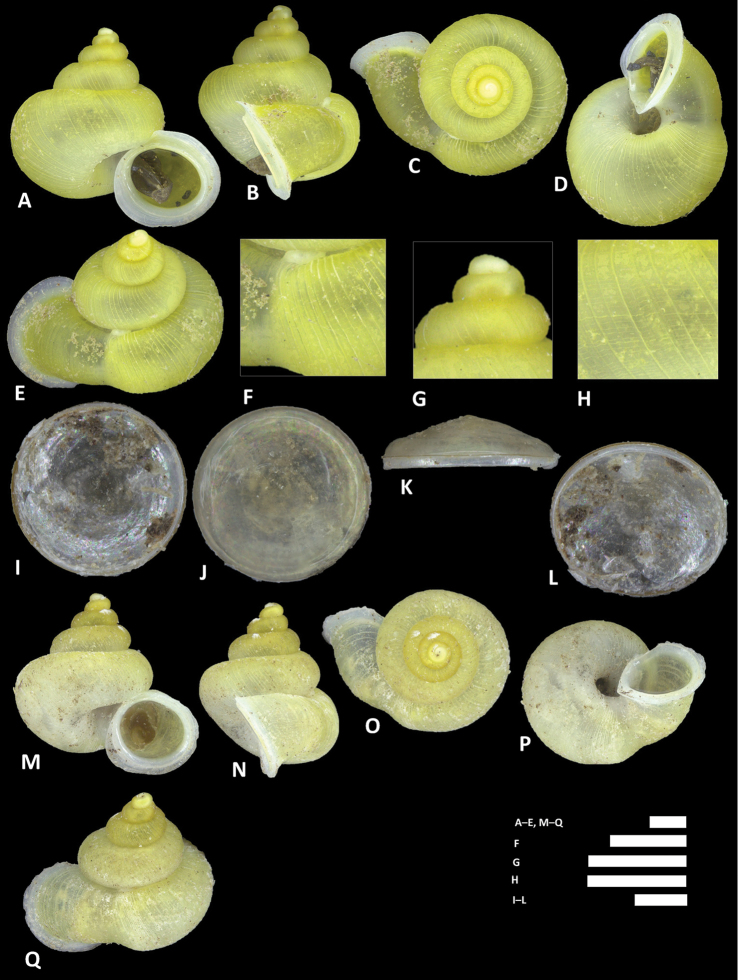
*Alycaeus expansus* sp. n. **A–E** Shell of holotype, BOR/MOL 12983 **F–H** Close up of shell of holotype, BOR/MOL 12983 **I–L** Operculum of holotype, BOR/MOL 12983 **M–Q** Shell of BOR/MOL 245. Scale bars: **A–H** 1 mm; **I–L** 0.5 mm. All photographs by Junn Kitt Foon.

Figures 7N , 16 , 31I

###### Type locality.

PHG 74 Bukit Sagu, Pahang (3°58'54"N, 103°08'39"E).

###### Type material.

*Holotype.* PHG 74 Bukit Sagu, Pahang: BOR/MOL 12983. *Paratypes.* PHG 74 Bukit Sagu, Pahang: BOR/MOL 269/2, BOR/MOL 6358/4. PHG 75 Bukit Tenggek, Pahang: BOR/MOL 273/2.

###### Other examined materials.

PHG 72 Bukit Panching, Pahang: BOR/MOL 247/1, ZRC 1975.2.24.352-355/3, ANSP 439118/8. PHG 73 Bukit Charas, Pahang: BOR/MOL 245/1, BOR/MOL 6367/1, BOR/MOL 6890/1, BOR/MOL 8400/9, BOR/MOL 12984/1, ZRC 1975.2.21.956-1015/63, ZRC 1975.2.21.876-955/81, ZRC 1975.2.21.435-488/53, ZRC 1975.2.21.732-831/101. PHG 74 Bukit Sagu, Pahang: ZRC 1975.2.21.532-556/26. PHG 75 Bukit Tenggek, Pahang: ZRC 1975.2.24.723-725/2, ZRC 1975.2.21.489-531/44.

###### Etymology.

Latin, meaning expanded. In reference to the very expanded peristome and globose whorls.

###### Description.

**Protoconch.** Smooth.

**Shell shape.** Conical. Shell height: 4.11–5.88 mm. Shell width: 3.87–5.96 mm.

**Spire.** Spire height: 1.33–1.85 mm. Spire width: 1.88–2.57 mm. Number of whorls: up to 4 ½. Spire shape: oblong conical. Whorl periphery rounded. Umbilicus open.

**Whorl constriction.** At about 4 ¼ whorls posterior of protoconch.

**Breathing tube.** Length: 0.44–0.62 mm.

**Aperture and peristome.** Aperture very expanded, ocassionally forming wing-like extensions at columellar and upper palatal sections. Wing-like extension at columellar section of the aperture is angular. Aperture height: 1.81–3.44 mm. Aperture width: 1.78–3.13 mm. Peristome double, not thickened, notched at suture. Peristome orientation 27–32° oblique with respect to the coiling axis.

**Spiral lines.** Indistinct. Regularly spaced. Approximately 18–40 lines per 1 mm.

**Radial ribs running anterior of breathing tube.** Radial ribs indistinct, evenly spaced. Approximately 6–15 ribs per 1 mm.

**Radial ribs running perpendicular to breathing tube.** Radial ribs pronounced and thicker than those anterior of breathing tube, evenly spaced. Approximately 11–17 ribs per 1 mm.

**Radial ribs running posterior of breathing tube.** Radial ribs indistinct, unevenly spaced to absent.

**Operculum.** Concave, conical. Exterior covered with thick calcareous layer, surface finely granulated. Interior covered with proteinaceous coating, smooth, mamillated.

**Shell colour.** Whorls yellow. Peristome white.

**Living animal.** Body cream-white. Head pink. Tentacles cream-white, tips pink.

###### Habitat and ecology.

Lives on wet rock surfaces and crevices covered with mosses at higher areas and wang of limestone hills. In shaded forests.

###### Distribution range.

Restricted to the Kuantan Hills of eastern Pahang (comprises of Bukit Tenggek, Bukit Sagu, Bukit Charas and Bukit Panching).

###### Differential diagnosis.

*Alycaeus expansus* sp. n. is distinguished from all other Peninsular Malaysian *Alycaeus* by its very obtuse ultimate whorl, very expanded peristome with wing-like extensions at columellar and upper palatal sections, thick operculum and animal colouration.

###### Discussion.

Prior to this study, shells of *A. expansus* sp. n. from Bukit Charas and Bukit Panching were labelled as *A. kelantanensis* (BOR/MOL and ZRC collection lots). Shells of *A. expansus* sp. n. is consistent in its diagnostic obtuse ultimate whorl and very expanded peristome across its range but varies in shell size and spire height for each hill population. Shells from Bukit Sagu and Bukit Tenggek (the northern two of the Kuantan Hills) tend to be larger with ultimate whorl always obtused and spire low. Shells from Bukit Charas and Bukit Panching (the southern two of the Kuantan Hills) tend to be slightly smaller and more variable in ultimate whorl shape (obtuse to normal) and spire height (tall to low). It is possible that *A. expansus* sp. n. may contain two cryptic species pending availability of molecular data for investigation. Unfortunately, populations on Bukit Panching, Bukit Tenggek and Bukit Sagu are now likely lost to limestone quarrying (see Discussion in *A. carinata*).

##### Alycaeus gibbosulus

Taxon classificationAnimaliaGastropodaAlycaeidae

Stoliczka, 1872

**Figure 17.**
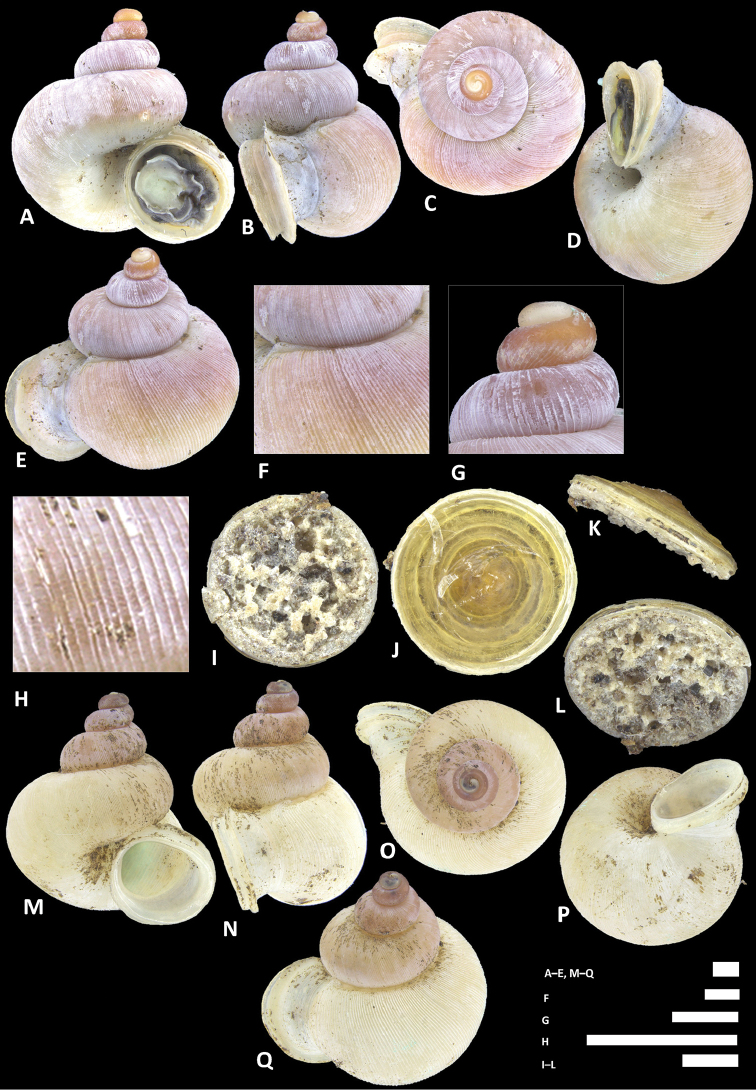
*Alycaeus gibbosulus* Stoliczka, 1872. **A–E** Shell of BOR/MOL 8525 **F–H** Close up of shell of BOR/MOL 8525 **I–L** Operculum of BOR/MOL 8525 **M–Q** Shell of BOR/MOL 12960. Scale bars: **A–Q** 1 mm. All photographs by Junn Kitt Foon.

Figures 7F–G , 17 , 31E

Alycaeus gibbosulus Stoliczka, 1872: 268–269, plate 10, figure 14; Pfeiffer 1876: 58; Nevill 1878: 295; Crosse 1879b: 339; Morgan 1885b: 402; Möllendorff 1886: 310; Tenison-Woods 1888: 1068; Kobelt 1902: 344; Sykes 1902: 62; Benthem Jutting 1960a: 13, 19; Davison 1995: 236; Maassen 2001: 21; Clements et al. 2008: 2760; Tarruella and Domènech 2011: 72, figure 1F; Foon et al. 2017: 12, figure 4C.
Alycaeus chaperi Morgan, 1885a: 70.
Alycaeus (Orthalycaeus) gibbosulus : Möllendorff 1891: 342; Kobelt and Möllendorff 1897: 150; Möllendorff 1902: 144.
Alycaeus gibbosulos [sic]: Berry 1963: plate 4.
Dioryx pyramidalis : Habe 1965: 111–112, plate 2, figures 3–4; Maassen 2001: 21. [not Alycaeus pyramidalis Benson, 1856]
Alycaeus (Alycaeus) gibbosulus : Laidlaw 1928: 34; Chan 1998b: 1.

###### Type locality.

Penang Island.

###### Type material.

*Syntypes.* Penang Island: 12 shells deposited at the Indian Museum, Kolkata (Nevill 1878) (Not seen).

###### Other examined materials.

Botanic Garden, Penang Island: BOR/MOL 8525/1, BOR/MOL 8526/12. KDH 01 Bukit Baling, Kedah: BOR/MOL 6855/1, BOR/MOL 8358/1, BOR/MOL 8359/23, BOR/MOL 12996/1, ZRC 1975.2.21.182-207/25, ZRC 1975.2.21.144-150/6, ZRC 1997.17/4, ANSP 423057/2. KDH 02 Gunung Pulai, Kedah: BOR/MOL 6857/1, BOR/MOL 8362/16. KDH 10 Bukit Keplu, Kedah: BOR/MOL 6871/1, BOR/MOL 8375/51, BOR/MOL 8376/1, BOR/MOL 6190/1. PRK 62 Gua Dayak, Perak: BOR/MOL 279/1. Mykarst-171 Tasik Temenggor Hill S2, Perak: BOR/MOL 6244/1. PRK 55 Gunung Pondok, Perak: BOR/MOL 6850/1, BOR/MOL 8351/3, BOR/MOL 8352/7, BOR/MOL 11523/1, BOR/MOL 11538/2, ZRC 1972.2.21.140-142/3. Limestone outcrop 50m from roadside, Kampung Pahit, Klian Intan, Perak: BOR/MOL 6875/1, BOR/MOL 8381/3, BOR/MOL 8382/1. Mykarst-027 Gunung Kanthan North, Perak: BOR/MOL 9106/2. Mykarst-025, Perak: BOR/MOL 9382/10, BOR/MOL 9416/2, BOR/MOL 9436/17, BOR/MOL 9500/11. PRK 23 Gunung Rapat, Perak: BOR/MOL 10286/1. PRK 59 Bukit Batu Kurau, Perak: ZRC 1975.2.21.180-181/4. Sungai Siput, Perak: ZRC 1975.2.21.143/1. Capis, upper Perak: ZRC 1975.2.21.1020-1022/3. Gua Kelam, PRS 64 Wang Ulu, Perlis: BOR/MOL 278/1, BOR/MOL 6866/1, BOR/MOL 6868/1, BOR/MOL 8372/4, ZRC 1975.2.21.152-179/27. PRS 19 Bukit Chuping, Perlis: BOR/MOL 6860/3, ZRC 1975.2.21.151/1. Bukit Ayer, PRS 64 Wang Ulu, Perlis: BOR/MOL 6862/1, BOR/MOL 8366/5, BOR/MOL 8367/3, BOR/MOL 12960/1. PRS 17 Gunung Chabang, Perlis: BOR/MOL 6870/1. PRS 40 Bukit Merbok, Perlis: BOR/MOL 8139/1. TRG 01 Bukit Bewah, Terengganu: BOR/MOL 6662/10, BOR/MOL 13002/1. Malakka (= Peninsular Malaysia): RMNH 153521/2.

###### Description.

**Protoconch.** Smooth at first whorl, becomes grooved in the second whorl. Grooves arranged parallel to each other and tilted anteriorly at 45° to the coiling axis.

**Shell shape.** Conical. Shell height: 9.20–10.15 mm. Shell width: 8.46–9.75 mm.

**Spire.** Spire height: 2.32–3.07 mm. Spire width: 3.32–3.67 mm. Number of whorls: up to 4 ⅛. Spire shape: oblong conical. Whorl periphery rounded. Umbilicus open.

**Whorl constriction.** At about 4 whorls posterior of protoconch.

**Breathing tube.** Length: 5.28–6.52 mm.

**Aperture and peristome.** Aperture circular, moderately expanded. Aperture height: 4.11–5.14 mm. Aperture width: 4.27–4.93 mm. Peristome double, thickened, slightly notched at suture. Interspace narrow to wide. Peristome orientation 18–24° oblique with respect to the coiling axis.

**Spiral lines.** Indistinct, regularly spaced. Approximately 20–44 lines per 1 mm.

**Radial ribs running anterior of breathing tube.** Radial ribs pronounced, evenly spaced. Approximately 14–21 ribs per 1 mm.

**Radial ribs running perpendicular to breathing tube.** Radial ribs more pronounced and thicker than those anterior of breathing tube, evenly spaced. Approximately 8–14 ribs per 1 mm.

**Radial ribs running posterior of breathing tube.** Ribs absent. Only radial growth lines.

**Operculum.** Concave, conical. Exterior covered by thick calcareous layer. Exterior has scaffold-like calcareous deposits overlaid on radially spiral lamellae. Interior covered by proteinaceous layer, mamillated.

**Shell colour.** First 3 whorls usually red, purple or pink. All colours fade to white towards ultimate whorl.

**Living animal.** Body brown. Head brown. Tentacles light to dark grey.

###### Habitat and ecology.

Lives under large rotten logs or on leaf litter. In wet, shady forests in both limestone and non-limestone areas.

###### Distribution.

Widespread from Perlis to Perak on the west coast and in parts of Kelantan and Terengganu on the east coast but not found in Selangor, Pahang and the southern states of Peninsular Malaysia. Elsewhere, in Trang, southern Thailand (as *Dioryx pyramidalis* (Benson, 1856), in Habe (1965)).

###### Differential diagnosis.

*Alycaeus gibbosulus* is most similar to *A. conformis* in shell shape but differs in having a more expanded penultimate whorl, coarser sculpture, more oblique peristome, longer constriction whorl, longer breathing tube (longer by 0.66 mm), ultimate whorl that is very obtused prior to constriction and scaffold-like calcareous deposits at the operculum exterior. The animal body is brown in *A. gibbosulus* compared to grey in *A. conformis*.

###### Discussion.

The syntypes of *A. gibbosulus* is housed in the Indian Museum, Kolkata, India (Nevill 1878) but was not examined in this study. Nevertheless, topotypic specimens from Penang Island (BOR/MOL 8525, BOR/MOL 8526) match the illustration of the type by Stoliczka (1872). This is considered to be sufficient for positive species identification.

This species is closely related to *A. conformis* and has historically been confused with it. Studies that reported *A. gibbosulus* in various localities are as follows: Penang (Stoliczka 1872, Crosse 1879b, Tenison-Woods 1888, Kobelt 1902, Laidlaw 1928, Möllendorff 1891), Perlis (Benthem Jutting 1960a), Perak (Crosse 1879b, Möllendorff 1886, Möllendorff 1891, Davison 1995, Chan 1998b) and Kelantan (Sykes 1902, Möllendorff 1902). Möllendorff (1886) reported that Perak shells differ from Penang shells in being paler as well as possessing a wider ultimate whorl. These shell forms are well within the morphological variation of *A. gibbosulus*. An erroneous record of *Dioryx pyramidalis* Benson, 1856 in Peninsular Malaysia has been traced back to a mistake by Habe (1965) in which specimens of *A. gibbosulus* from northern Malay Peninsula were misidentified as *Alycaeus pyramidalis* Benson, 1856. The species *Alycaeus chaperi* Morgan, 1885b, is also a synonym of *Alycaeus gibbosulus* (Möllendorff 1886, Möllendorff 1891).

*Alycaeus gibbosulus* has been reported to exist sympatrically with *A. conformis* in Gunung Rapat, Perak (Foon et al. 2017). Elsewhere range overlaps between *A. gibbosulus* and *A. conformis* remain unresolved. In Kelantan, Sykes (1902) mentioned a collection of shells with typical *A. gibbosulus* characters and some which were “much less gibbous”. These less gibbous shells are likely *A. conformis*, which is common in Kelantan. Hence, it is possible that both *A. gibbosulus* and *A. conformis* exist sympatrically in Kelantan. In Pahang where only *A. conformis* records are confirmed, an aberrant record of *A. gibbosulus* from Kuala Tahan (Benthem Jutting 1960a) warrants investigation. The shells of *A. gibbosulus* are sometimes coated in a layer of organic matter when alive (Figure 7F).

##### Alycaeus ikanensis sp. n.

Taxon classificationAnimaliaGastropodaAlycaeidae

http://zoobank.org/DBC1A3CE-F85A-4F42-8188-63607F148C5C

Figures 18 , 31L

Alycaeus perakensis var. minor nomen nudum, Clements et al. 2008: 2760.

###### Type locality.

KTN 06 Gua Ikan, Kelantan (5°21'14"N, 102°01'33"E).

###### Type material.

*Holotype.* KTN 06 Gua Ikan, Kelantan: BOR/MOL 12972. *Paratypes.* KTN 06 Gua Ikan, Kelantan: BOR/MOL 6834/23.

###### Other examined materials.

KTN 06 Gua Ikan, Kelantan: BOR/MOL 6193/2, BOR/MOL 6632/17.

###### Etymology.

Named after the type locality, Gua Ikan.

###### Description.

**Protoconch.** Smooth.

**Shell shape.** Conical. Shell height: 4.02–4.58 mm. Shell width: 3.58–4.16 mm.

**Spire.** Spire height: 1.42–1.78 mm. Spire width: 1.83–2.03 mm. Number of whorls: up to 4 ½. Spire shape: oblong conical. Whorl periphery rounded. Umbilicus open.

**Whorl constriction.** At about 4 ¼ whorls posterior of protoconch.

**Breathing tube.** Length: 0.35–0.42 mm.

**Aperture and peristome.** Aperture circular, moderately expanded. Aperture height: 1.59–1.87 mm. Aperture width: 1.60–1.96 mm. Peristome double, not thickened, notched at suture. Peristome orientation 16–27° oblique with respect to the coiling axis.

**Spiral lines.** Distinct. Regularly spaced. Approximately 22–30 lines per 1 mm.

**Radial ribs running anterior of breathing tube.** Radial ribs pronounced, evenly spaced. Approximately 9–19 ribs per 1 mm.

**Radial ribs running perpendicular to breathing tube.** Radial ribs pronounced, white and thicker than those anterior of breathing tube, evenly spaced. Approximately 13–18 ribs per 1 mm.

**Radial ribs running posterior of breathing tube.** Radial ribs absent immediately posterior of constriction, becoming pronounced and unevenly spaced towards the aperture.

**Operculum.** Concave, rounded. Exterior covered with very thin and indistinct calcareous layer, surface finely granulated, with very indistinct appressed multilamellae. Interior proteinaceous layered, smooth, very indistinct appressed multilamellae.

**Shell colour.** Whorls yellow, fading to white posterior of constriction. Peristome white.

**Living animal.** Unknown.

###### Habitat and ecology.

Lives on limestone walls and boulders covered with mosses. In shady forests on limestone hills.

###### Distribution range.

Restricted to Gua Ikan, central Kelantan.

###### Differential diagnosis.

*Alycaeus ikanensis* sp. n. is distinctive in being the smallest species among conical, yellow shelled *Alycaeus* species in Peninsular Malaysia. *Alycaeus ikanensis* sp. n. is distinguished from *A. costacrassa* sp. n. by its consistently much smaller shell (smaller by about 0.62 mm in shell height, 0.90 mm in shell width) and narrower ultimate whorl.

###### Discussion.

Limestone hills adjacent to the type locality Gua Ikan may also harbour populations of the species.

**Figure 18.**
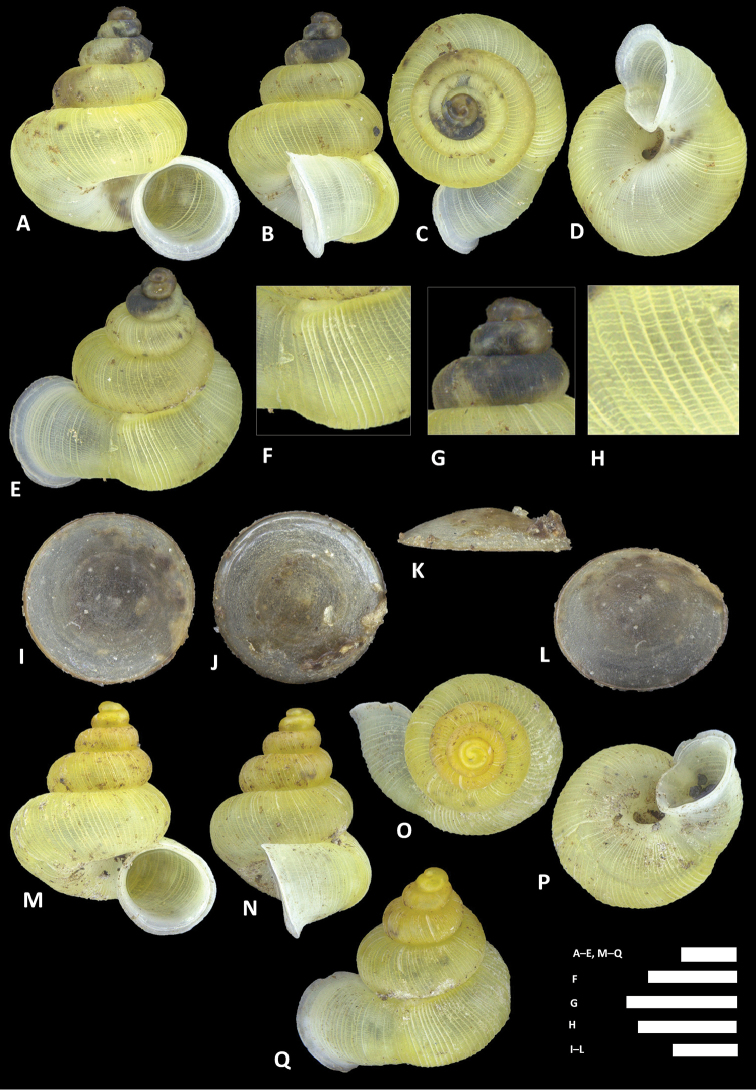
*Alycaeus ikanensis* sp. n. **A–E** Shell of holotype, BOR/MOL 12972 **F–H** Close up of shell of holotype, BOR/MOL 12972 **I–L** Operculum of holotype, BOR/MOL 12972 **M–Q** Shell of paratype, BOR/MOL 6834. Scale bars: **A–H, M–Q** 1 mm; **I–L** 0.5 mm. All photographs by Junn Kitt Foon.

##### Alycaeus jousseaumei

Taxon classificationAnimaliaGastropodaAlycaeidae

Morgan, 1885a

**Figure 19.**
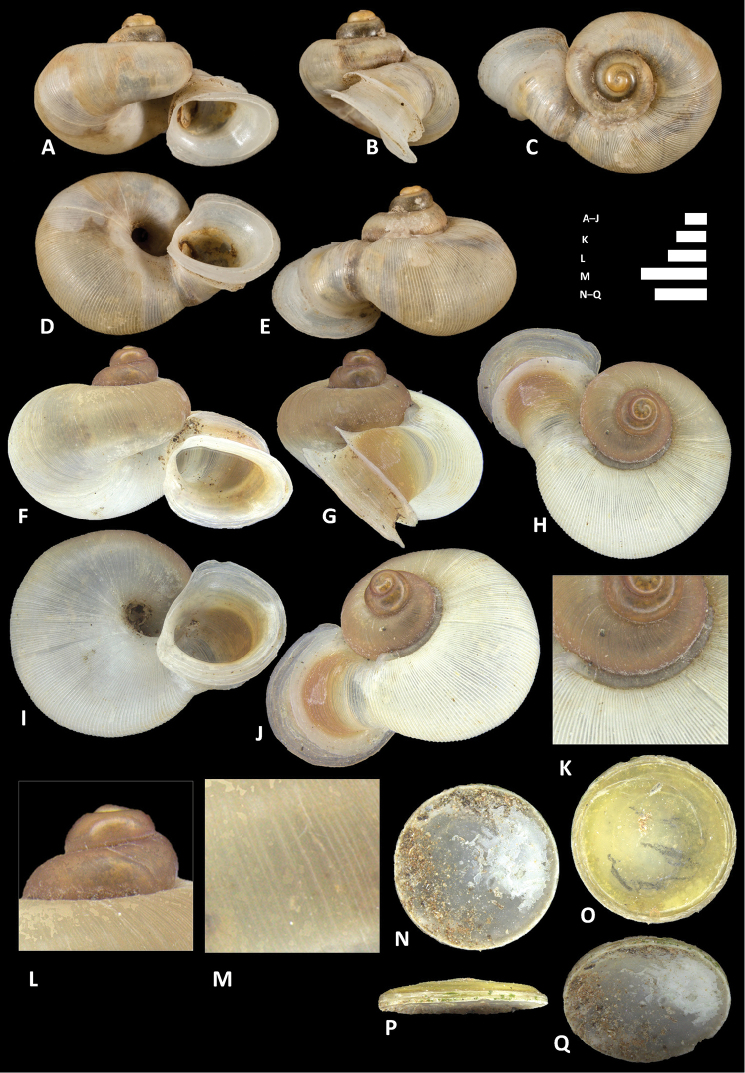
*Alycaeus jousseaumei* Morgan, 1885a. **A–E** Shell of syntype, MNHN-IM-2000-31801 **F–J** Shell of BOR/MOL 11211 **K–M** Close up of shell of BOR/MOL 11211 **N–Q** Operculum of BOR/MOL 11211. Scale bars: **A–Q** 1 mm. Photographs **A–E** by Manuel Caballer (reproduced herein under CC-BY 4.0 license, Project RECOLNAT, MNHN). Photographs **F–V** by Junn Kitt Foon.

Figures 7H–I , 19 , 31F

Alycaeus jousseaumei Morgan, 1885a: 70; Morgan 1885b: 402, plate 8, figure 4; Möllendorff 1886: 312; Möllendorff 1891: 343; Tarruella and Domènech 2011: 72, figure 2B; Foon et al. 2017: 12, figure 4D.
Alycaeus (Chamalycaeus) jousseaumi [sic]: Kobelt and Möllendorff 1897: 151.
Alycaeus (Chamalycaeus) jousseaumei : Kobelt 1902: 357; Laidlaw 1928: 36; Chan 1998a: 4; Chan 1998b: 1.
Chamalycaeus jousseaumei : Benthem Jutting 1960a: 13; Berry 1963: plate 4; Maassen 2001: 24; Clements et al. 2008: 2760.

###### Type locality.

Mont Lano (= PRK 18 Gunung Lanno), Perak (4°31'31"N, 101°08'48"E).

###### Type material.

*Syntypes*. Mont Lano (= PRK 18 Gunung Lanno), Perak: MNHN IM-2000-31800/1 (Not seen), MNHN IM-2000-31801/1 (Seen).

###### Other examined materials.

PRK 01 Gunung Tempurung, Perak: BOR/MOL 6847/1, BOR/MOL 8346/3, BOR/MOL 11136/11, BOR/MOL 11211/37, BOR/MOL 11408/10. PRK 15 Gunung Keroh, Perak: ZRC 1975.2.21.247-248/2. PRK 18 Gunung Lanno, Perak: BOR/MOL 6841/1, BOR/MOL 6916/3, BOR/MOL 8337/2, BOR/MOL 8338/2. PRK 23 Gunung Rapat, Perak: BOR/MOL 293/5, BOR/MOL 6255/1, BOR/MOL 6844/1, BOR/MOL 6846/1, BOR/MOL 6848/1, BOR/MOL 7133/6, BOR/MOL 8341/14, BOR/MOL 8342/2, BOR/MOL 8345/22, BOR/MOL 8347/25, BOR/MOL 8348/4, BOR/MOL 10046/8, BOR/MOL 10202/3, BOR/MOL 10228/9, BOR/MOL 10253/10, BOR/MOL 12963/1, BOR/MOL 12998/2, ZRC 1997.18/2. PRK 27 Gunung Datok, Perak: BOR/MOL 10412/9, BOR/MOL 10441/8, BOR/MOL 10474/30, BOR/MOL 10494/8. PRK 34 Gunung Tasek, Perak: BOR/MOL 6839/1, BOR/MOL 8336/6, BOR/MOL 10788/7, BOR/MOL 11054/1. PRK 42 Gunung Bercham, Perak: BOR/MOL 10593/4. PRK 47 Gunung Kanthan South, Perak: BOR/MOL 9101/2, ZRC 1975.2.21.2/2. PRK 53 Hill KF, Perak: BOR/MOL 10658/12, BOR/MOL 10689/13, BOR/MOL 10717/5. Mykarst-189, Perak: BOR/MOL 6842/1, BOR/MOL 8339/8. Mykarst-068, Perak: BOR/MOL 6843/1, BOR/MOL 8340/1, RMNH 153708/3. Mykarst-025, Perak: BOR/MOL 9383/1. Mykarst-027 Gunung Kanthan North, Perak: BOR/MOL 9053/3, BOR/MOL 9374/1. Mykarst-070 Kramat Pulai, Perak: ZRC 1975.2.21.238-245/8. Perak: RMNH 153477/1.

###### Description.

**Protoconch.** Smooth.

**Shell shape.** Flat. Shell height: 7.50–8.76 mm. Shell width: 12.42–15.03 mm.

**Spire.** Spire height: 1.77–2.27 mm. Spire width: 2.94–3.45 mm. Number of whorls: up to 3. Spire shape: oblong conical. Whorl periphery rounded. Umbilicus open.

**Whorl constriction.** At about 2 ¾ whorls posterior of protoconch.

**Breathing tube.** Length: 5.44–5.97 mm.

**Aperture and peristome.** Aperture circular, very expanded. Aperture height: 4.88–7.30 mm. Aperture width: 4.92–6.59 mm. Peristome triple. The first (most anterior) peristome is moderately expanded but not thickened. The second (outer) peristome is very expanded but not thickened. The second peristome is impressed on the thickened third (inner), non-expanded peristome. Interspace between first and second peristome wide. Interspace between second and third peristome narrow. Peristome orientation is 44–55° oblique with respect to the coiling axis.

**Spiral lines.** Absent.

**Radial ribs running anterior of breathing tube.** Radial ribs indistinct, evenly spaced. Approximately 10–18 ribs in 1 mm.

**Radial ribs running perpendicular to breathing tube.** Radial ribs pronounced and thick, evenly spaced. Approximately 8–15 ribs in 1 mm.

**Radial ribs running posterior of breathing tube.** Radial ribs pronounced, evenly spaced. Approximately 10–25 ribs per 1 mm.

**Operculum.** Flat, conical. Exterior calcareous, smooth but occasionally with calcareous deposits. Interior covered by proteinaceous layer, smooth.

**Shell colour.** First 3 whorls usually red, orange, purple or white. Ultimate whorl white. Whorl constriction generally white, occasionally red.

**Living animal.** Body light grey to light brown. Head pinkish-brown to dark grey. Tentacles brown.

###### Habitat and ecology.

Lives on limestone rock surfaces and crevices. In wet, shady forests on limestone hills.

###### Distribution range.

Restricted to the karsts of Kinta Valley, Perak. Its known northern limit is at Gunung Tchehel and southern limit at Gunung Tempurung.

###### Differential diagnosis.

*Alycaeus jousseaumei* is distinguished from all other *Alycaeus* species in Peninsular Malaysia by its very low spire, very expanded, obtuse ultimate whorl and very expanded, double peristome with wide interspace.

###### Discussion.

*Alycaeus jousseaumei* is morphologically unique and unmistakable among Peninsular Malaysian *Alycaeus*. *Alycaeus jousseaumei* has been subjected to attempts of supraspecific reclassification. Morgan (1885a) initially placed *A. jousseaumei* in the genus *Alycaeus* but Kobelt and Möllendorff (1897) reassigned *A. jousseaumei* in *Chamalycaeus* without any justification. The genus *Chamalycaeus* in Peninsular Malaysia is characterised by its small to minute shell (shell width <6.0 mm), discoid shell shape, strong radial ribs and a breathing tube of variable length (Kobelt 1902, Maassen 2001, Egorov 2013, Foon et al. 2017). *Alycaeus jousseaumei* is clearly not a *Chamalycaeus* species. Thus, *Alycaeus jousseaumei* is herein reassigned back to *Alycaeus*. *Alycaeus jousseaumei* is remarkably similar to *A. kapayanensis* in being range-restricted to the karsts of Kinta Valley (Morgan 1885a, 1885b, Möllendorff 1886, Möllendorff 1891, Benthem Jutting 1960a, Chan 1998a, 1998b).

##### Alycaeus kapayanensis

Taxon classificationAnimaliaGastropodaAlycaeidae

Morgan, 1885b

**Figure 20.**
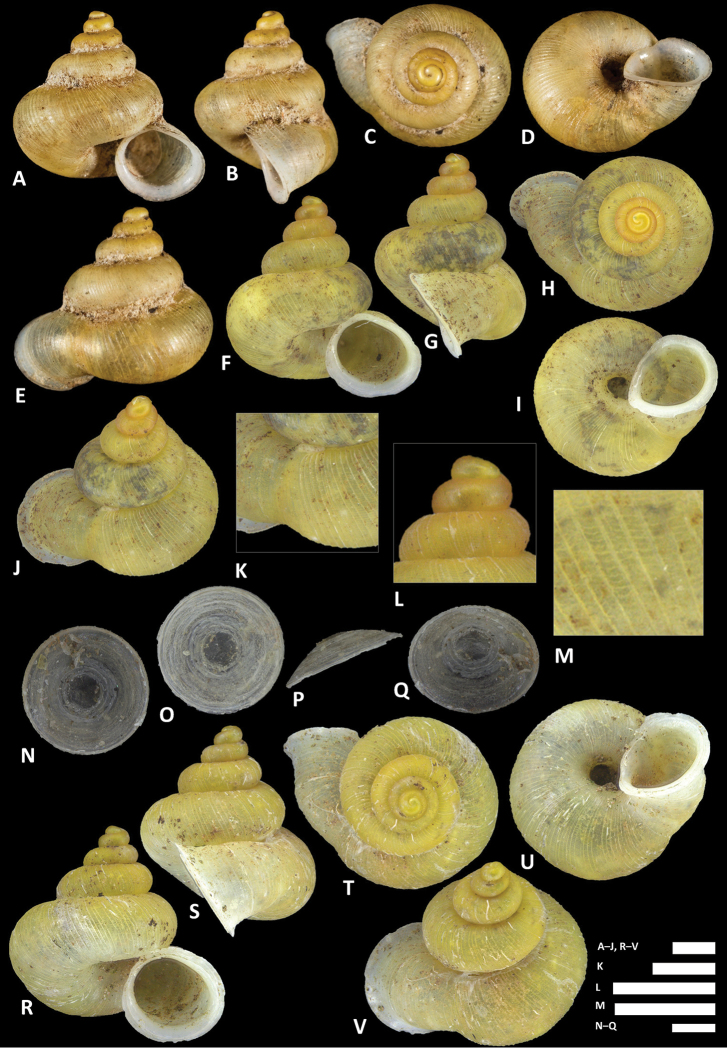
*Alycaeus kapayanensis* Morgan, 1885b. **A–E** Shell of syntype, MNHN-IM-2000-31792 **F–J** Shell of BOR/MOL 10793 **K–M** Close up of shell of BOR/MOL 10793 **N–Q** Operculum of BOR/MOL 6845 **R–V** Shell of BOR/MOL 11385. Scale bars: **A–L** 1 mm; **M–Q** 0.5 mm. Photographs **A–E** by Manuel Caballer (reproduced herein under CC-BY 4.0 license, Project RECOLNAT, MNHN). Photographs **F–V** by Junn Kitt Foon.

Figures 7O , 20 , 31M

Alycaeus kapayanensis Morgan, 1885b: 403, plate 8, figure 5; Benthem Jutting 1960a: 13; Maassen 2001: 22; Tarruella and Domènech 2011: 72.
Alycaeus (Orthalycaeus) kapayensis [sic]: Möllendorff 1891: 342.
Alycaeus (Orthalycaeus) kapayanensis : Kobelt and Möllendorff 1897: 150.
Alycaeus (Alycaeus) kapayanensis : Kobelt 1902: 346; Laidlaw 1928: 35.
Alycaeus perakensis var. minor nomen nudum, Clements et al. 2008: 2760.
Alycaeus kapayanesis [sic]: Foon et al. 2017: 12, figure 4E.

###### Type locality.

Gunong Lano (= PRK 18 Gunung Lanno), Perak (4°31'31"N, 101°08'48"E).

###### Type material.

*Syntype.* Perak, Gunong Lano (= PRK 18 Gunung Lanno), Perak: MNHN IM-2000-31792/45 (1 specimen seen).

###### Other examined materials.

PRK 01 Gunung Tempurung, Perak: BOR/MOL 11142/11, BOR/MOL 11229/1, BOR/MOL 11385/2, BOR/MOL 11414/3. PRK 23 Gunung Rapat, Perak: BOR/MOL 241/2, BOR/MOL 6845/1, BOR/MOL 8343/4, BOR/MOL 8344/2, BOR/MOL 10031/4, BOR/MOL 10230/10, BOR/MOL 10259/4, BOR/MOL 12985/1. PRK 27 Gunung Datok, Perak: BOR/MOL 10482/4, BOR/MOL 10502/3. PRK 29 Gunung Lang, Perak: BOR/MOL 244/1, BOR/MOL 13005/13. PRK 34 Gunung Tasek, Perak: BOR/MOL 6840/5, BOR/MOL 6905/1, BOR/MOL 8405/11, BOR/MOL 8406/20, BOR/MOL 10793/52, BOR/MOL 11028/29, BOR/MOL 11065/5. PRK 42 Gunung Bercham, Perak: BOR/MOL 10629/1. PRK 47 Gunung Kanthan South, Perak: BOR/MOL 9081/38, BOR/MOL 9161/12, ZRC 1975.2.22.229/1. PRK 53 Hill KF, Perak: BOR/MOL 10671/3, BOR/MOL 10696/6. Hill 10 km NE of Sungai Siput town, Perak: BOR/MOL 246/1. Mykarst-025, Perak: BOR/MOL 9425/1, RMNH 153570/1. Mykarst-027 Gunung Kanthan North, Perak: BOR/MOL 9041/2. Limestone outcrop opposite Jalan Jalong-Lasah Chinese cemetery, NE of Sungai Siput, Perak; BOR/MOL 6876/1, BOR/MOL 8383/21, BOR/MOL 8384/1. Sungai Siput North, Perak: ZRC 1975.2.22.183-189/7, ZRC 1975.2.22.383-443/61, RMNH 153569/101, ZMA 33450/1.

###### Description.

**Protoconch.** Smooth.

**Shell shape.** Conical. Shell height: 4.85–5.34 mm. Shell width: 4.44–5.09 mm.

**Spire.** Spire height: 1.66–2.03 mm. Spire width: 2.11–2.39 mm. Number of whorls: up to 5. Spire shape: oblong conical. Whorl periphery rounded. Umbilicus open.

**Whorl constriction.** At about 4 ⅝ whorls posterior of protoconch.

**Breathing tube.** Length: 0.29–0.51 mm.

**Aperture and peristome.** Aperture circular, moderately expanded. Aperture height: 2.01–2.77 mm. Aperture width: 2.13–2.36 mm. Peristome single, not thickened, notched at suture. Peristome orientation 29–32° oblique with respect to the coiling axis.

**Spiral lines.** Distinct. Regularly spaced. Approximately 27–48 lines per 1 mm.

**Radial ribs running anterior of breathing tube.** Radial ribs pronounced, evenly spaced. Approximately 11–15 ribs per 1 mm.

**Radial ribs running perpendicular to breathing tube.** Radial ribs pronounced, evenly spaced. Approximately 10–19 ribs per 1 mm.

**Radial ribs running posterior of breathing tube.** Radial ribs absent immediately posterior of constriction, becoming pronounced and evenly spaced at the middle until prior to aperture. Approximately 11–23 ribs per 1 mm.

**Operculum.** Concave, rounded. Exterior covered with very thin calcareous layer, with indistinct appressed multilamellae. Interior covered with proteinaceous coating, smooth indistinct appressed multilamellae.

**Shell colour.** Whorls yellow. Peristome white.

**Living animal.** Body yellow. Head orange. Tentacles red.

###### Habitat and ecology.

Lives on both dry, bare rock and wet limestone walls covered with mosses or tree roots. Although it inhabits forested areas, *A. kapayanensis* has also been found on revegetated limestone walls at former quarries.

###### Distribution range.

Restricted to the karsts of Kinta Valley, Perak. Its known northern limit is at the Sungai Kerdah karst region, northeast of Sungai Siput Utara town and southern limit at Gunung Tempurung.

###### Differential diagnosis.

*Alycaeus kapayanensis* is similar to *A. costacrassa* sp. n. but differs consistently in having less pronounced spiral ribs and much thinner operculum. *Alycaeus kapayanensis* also resembles *A. selangoriensis* sp. n. but is consistently smaller shelled (smaller by about 0.96 mm in shell height, 1.6 mm in shell width), has less whorls, less expanded ultimate whorl and thinner operculum.

###### Discussion.

Historically, many Peninsular Malaysian *Alycaeus* with small, yellow and conical shells were lumped under *A. kapayanensis* out of convenience (ZRC collection lots). We systematically and empirically examined these shells from across the peninsula and concluded that *A. kapayanensis* is restricted to Kinta Valley. Superficially similar species elsewhere differ consistently in shell, operculum and animal characters and are thus described as new taxa: *Alycaeus costacrassa* sp. n., *Alycaeus selangoriensis* sp. n., *Alycaeus ikanensis* sp. n., *Alycaeus alticola* sp. n. and *Alycaeus charasensis* sp. n. In Kinta Valley, many *A. kapayanensis* shells have previously been labelled as “A. perakensis var. minor”, a nomen nudum (Clements et al. 2008, ZRC collection lots). The only morphological variability in *A. kapayanensis* is the degree of expansion of the ultimate whorl.

##### Alycaeus kelantanensis

Taxon classificationAnimaliaGastropodaAlycaeidae

Sykes, 1902

**Figure 21.**
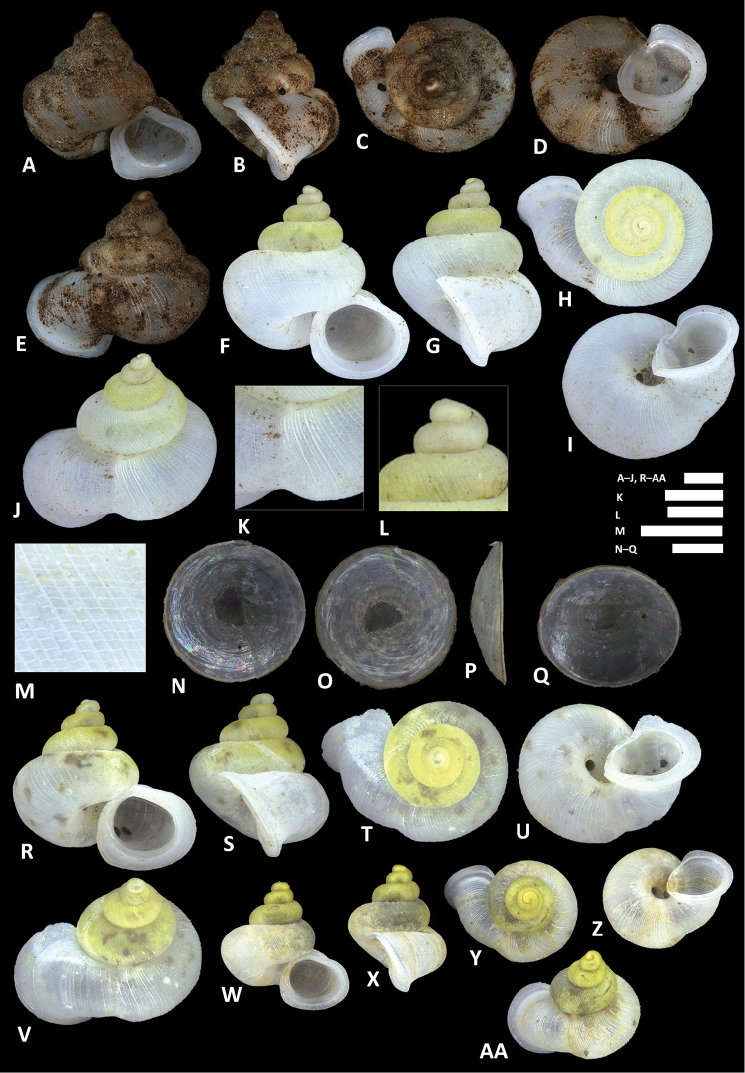
*Alycaeus kelantanensis* Sykes, 1902. **A–E** Shell of syntype, RMNH 153594 **F–J** Shell of BOR/MOL 8327 **K–M** Close up of shell of BOR/MOL 8327 **N–Q** Operculum of BOR/MOL 6825 **R–V** Shell of BOR/MOL 12973 **W–AA** Shell of BOR/MOL 12974. Scale bars: **A–J, K, R–AA** 1 mm; **L, M, N–Q** 0.5 mm. Photographs **A–E** by Thor Seng Liew. Photographs **F–AA** by Junn Kitt Foon.

Figures 7P–Q , 21 , 31K

Alycaeus kelantanense Sykes, 1902: 60–62, Figure 11–12.
Alycaeus kelantanensis : Möllendorff 1902: 135–149; Laidlaw 1928: 35; Berry 1965: 28; Schilthuizen et al. 1999: 353; Maassen 2001: 22; Clements et al. 2008: 2760; Liew 2010: 101; Tarruella and Domènech 2011: 72, figure 2C.

###### Type locality.

Kelantan, Malay Peninsula.

###### Type material.

*Syntype.* Kelantan: RMNH 153594/1 (Seen).

###### Other examined materials.

KTN 01 Gunung Reng, Kelantan: BOR/MOL 6837/1. KTN 45 unnamed hill at Kampung Bayu, Kelantan: BOR/MOL 6236/6, BOR/MOL 12974/1. KTN 66 Batu Tongkat, Kelantan: ZRC 1975.2.21.838-875/44. KTN 77 Bukit Sejuk, Kelantan: BOR/MOL 6199/3, BOR/MOL 6231/1. KTN 95 Gua Panjang, Kelantan: BOR/MOL 6200/6, BOR/MOL 6829/1, BOR/MOL 8329/13, BOR/MOL 8330/5. KTN 109 part of Gua Panjang, Kelantan: BOR/MOL 12973/1. KTN 134 Gua Batu Boh, Kelantan: BOR/MOL 6828/1, BOR/MOL 8328/3, BOR/MOL 8409/3. KTN 135 Gua Serai, Kelantan: BOR/MOL 242/1. KTN 136 Gua Musang, Kelantan: ZRC 1975.2.21.250-349/102, ZRC 1975.2.24.452-476/25, ZRC 1975.2.24.309-351/43. KTN 139 unnamed hill, Kelantan: BOR/MOL 6197/5. KTN 148 ‘Hill 001’, Kelantan: BOR/MOL 12976/4. KTN 149 unnamed hill, Kelantan: BOR/MOL 6833/1, BOR/MOL 8333/11. KTN 176 Batu Lesong, Kelantan: BOR/MOL 6830/1, BOR/MOL 8331/7, BOR/MOL 8332/8. Mykarst-168, Kelantan: BOR/MOL 6189/2, BOR/MOL 6838/2. Mykarst-186 Gua Madu, Kelantan: BOR/MOL 6234/1, BOR/MOL 6825/1, BOR/MOL 8327/7, ZRC 1975.2.21.350-430/91. Limestone outcrop 3 km north of Felda Ciku 5 settlement, Kelantan: BOR/MOL 6238/3. Bukit Gua Peraling, Kelantan: ZRC 2.24.440-451/12. Kelantan: RMNH 153593/3, ZMA 33451/2. PHG 40 Gua Layang, Pahang: BOR/MOL 6822/1, BOR/MOL 8325/2, BOR/MOL 8326/7. PHG 114 ‘Boomerang’ Hill, Pahang: BOR/MOL 6188/2. Unnamed limestone hill north of Jalan Lipis-Gua Musang Lama, Felda Chegar Perah I and II, Pahang: BOR/MOL 6233/1, BOR/MOL 6814/1, BOR/MOL 8321/5, BOR/MOL 8322/3.

###### Description.

**Protoconch.** Smooth.

**Shell shape.** Conical. Shell height: 3.33–5.04 mm. Shell width: 3.36–5.04 mm.

**Spire.** Spire height: 1.08–1.76 mm. Spire width: 1.40–2.25 mm. Number of whorls: up to 4. Spire shape: oblong conical. Whorl periphery rounded. Umbilicus open.

**Whorl constriction.** At about 3 ⅝ whorls posterior of protoconch.

**Breathing tube.** Length: 0.38–0.67 mm.

**Aperture and peristome.** Aperture circular, moderately expanded to very expanded. Aperture height: 1.82–2.73 mm. Aperture width: 1.69–2.46 mm. Peristome double, thickened, folded posteriorly into a wing-like upper palatal structure, notched at suture. Peristome orientation 26–34° oblique with respect to the coiling axis.

**Spiral lines.** Distinct. Regularly spaced. Approximately 15–28 lines per 1 mm.

**Radial ribs running anterior of breathing tube.** Radial ribs pronounced, evenly spaced. Approximately 12–18 ribs per 1 mm.

**Radial ribs running perpendicular to breathing tube.** Radial ribs pronounced, white and thicker than those anterior of breathing tube, evenly spaced. Approximately 12–29 ribs per 1 mm.

**Radial ribs running posterior of breathing tube.** Radial ribs absent immediately posterior of constriction, becoming pronounced and evenly spaced at the middle, absent again towards the aperture. Approximately 11–20 ribs per 1 mm.

**Operculum.** Concave, rounded. Exterior covered with thin calcareous layer, with very indistinct appressed multilamellae. Interior covered with proteinaceous coating, smooth, with indistinct mamilla.

**Shell colour.** Whorls white throughout. First 3 whorls occasionally yellow.

**Living animal.** Body cream-white. Head pinkish. Tentacles yellow, with red tips.

###### Habitat and ecology.

Lives in rock crevices and limestone solution holes. In moist, shady forests. Occur on both limestone and non-limestone areas.

###### Distribution range.

Widespread across southern Kelantan.

###### Differential diagnosis.

*Alycaeus kelantanensis* varies in shell size and whorl convexity but can nevertheless be distinguished from other similar sized *Alycaeus* by its rather angular and winged double peristome, pronounced and widely-spaced radial ribs and spiral lines, as well as the red-tipped yellow tentacles of its soft body. *Alycaeus kelantanensis* is similar to *A. clementsi* sp. n. in shell colour but differs in being consistently smaller shelled (smaller by about 2.13 mm in shell height, 2.32 mm in shell width), having smooth operculum exterior and a more angular double peristome.

###### Discussion.

The type specimen for *A. kelantanensis* was collected by John Waterstradt, likely from the Pulai Princess Cave hill (4°47'38"N, 101°56'31"E), south of Gua Musang, Kelantan (after Liew et al. 2014, Waterstradt 1902). *Alycaeus kelantanensis* shells from across its range are consistent in their diagnostic characters but varies in shell size within and between populations. Aside from the locations listed above, this species has also been recorded from limestone hills near Kuala Betis, 25 km west of Gua Musang, Kelantan (Berry 1965) and in the non-limestone Lojing Highlands, Kelantan (Liew 2010).

##### Alycaeus kurauensis sp. n.

Taxon classificationAnimaliaGastropodaAlycaeidae

http://zoobank.org/FFC4471A-0820-4048-9572-0C55BBF5F7F1

**Figure 22.**
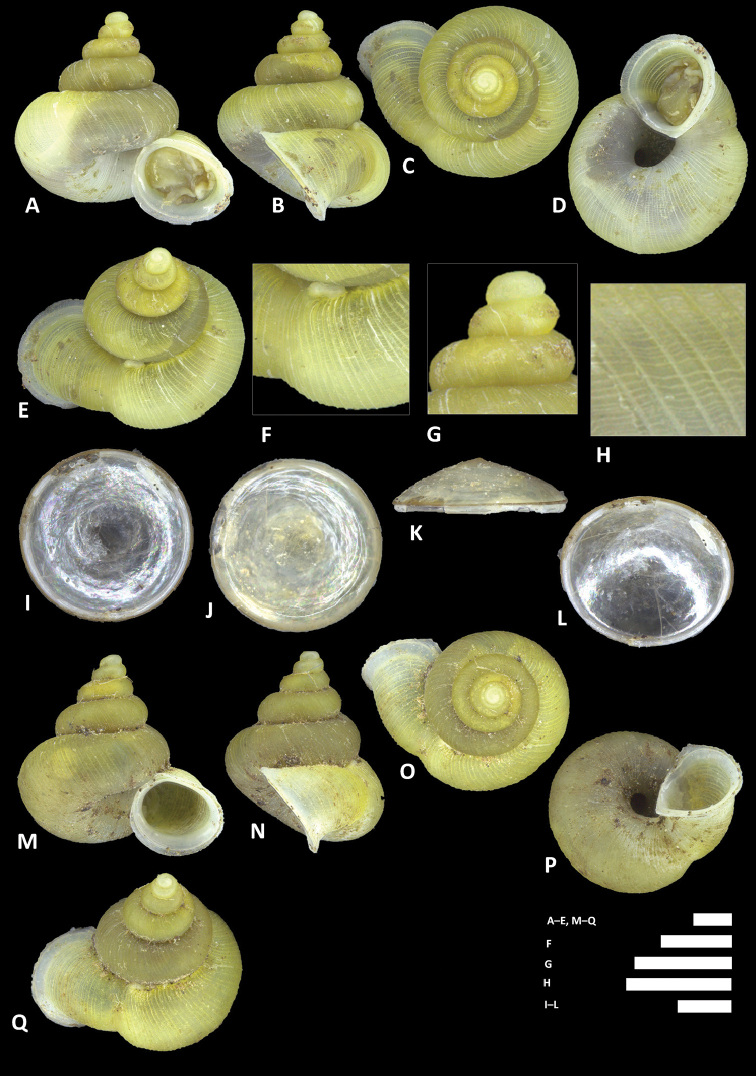
*Alycaeus kurauensis* sp. n. **A–E** Shell of holotype, BOR/MOL 12967 **F–H** Close up of shell of holotype, BOR/MOL 12967 **I–L** Operculum of paratype, BOR/MOL 6851 **M–Q** Shell of paratype, BOR/MOL 6851. Scale bars: **A–E, F, G, M–Q** 1 mm; **H, I–L** 0.5 mm. All photographs by Junn Kitt Foon.

Figures 7R , 22 , 31P

###### Type locality.

PRK 59 Bukit Batu Kurau, Perak (4°55'45"N, 100°49'02"E).

###### Type material.

*Holotype.* PRK 59 Bukit Batu Kurau, Perak: BOR/MOL 12967. *Paratypes.* PRK 59 Bukit Batu Kurau, Perak: BOR/MOL 6851/10, BOR/MOL 8408/2.

###### Other examined materials.

PRK 59 Bukit Batu Kurau, Perak: BOR/MOL 8353/22, ZRC 1975.2.22.230-284/54.

###### Etymology.

Named after the type locality, Batu Kurau.

###### Description.

**Protoconch.** Smooth.

**Shell shape.** Conical. Shell height: 5.04–5.56 mm. Shell width: 5.34–6.06 mm

**Spire.** Spire height: 1.08–2.06 mm. Spire width: 2.34–2.52 mm. Number of whorls: up to 4. Spire shape: oblong conical. Whorl periphery rounded. Umbilicus open.

**Whorl constriction.** At about 3 ⅝ whorls posterior of protoconch.

**Breathing tube.** Length: 0.45–0.62 mm.

**Aperture and peristome.** Aperture circular, moderately expanded. Aperture height: 2.51–2.84 mm. Aperture width: 2.34–2.65 mm. Peristome double, thickened, notched at suture. Peristome orientation 35–45° oblique with respect to the coiling axis.

**Spiral lines.** Distinct. Regularly spaced. Approximately 24–36 lines per 1 mm.

**Radial ribs running anterior of breathing tube.** Radial ribs pronounced, evenly spaced. Approximately 7–16 ribs per 1 mm.

**Radial ribs running perpendicular to breathing tube.** Radial ribs pronounced and thicker than those anterior of breathing tube, evenly spaced. Approximately 9–16 ribs per 1 mm.

**Radial ribs running posterior of breathing tube.** Radial ribs absent immediately posterior of constriction. At the middle section, becoming pronounced and evenly spaced from the middle section until prior to aperture. Approximately 10–15 ribs per 1 mm.

**Operculum.** Concave, rounded. Exterior covered with thick calcareous layer, smooth. Interior covered with proteinaceous coating, smooth, mamillated.

**Shell colour.** Whorls yellow. Peristome white.

**Living animal.** Body cream-yellow. Head orange. Tentacles red.

###### Habitat and ecology.

Lives on both dry and wet limestone walls. In shady forests and cliffs on limestone hills.

###### Distribution range.

Restricted to Batu Kurau, Perak.

###### Differential diagnosis.

*Alycaeus kurauensis* sp. n. is similar to *Alycaeus perakensis* in conical shell shape and radial ribs spacing but differs in being consistently smaller (by about 1.33 mm in shell height, and by about 1.85 mm in shell width), has a less expanded ultimate whorl and peristome, a thick operculum with smooth exterior as well as body and tentacle colouration. *Alycaeus kurauensis* sp. n. is also similar to *Alycaeus kapayanensis* in size and colour but differs in having a wider ultimate whorl, thicker operculum with smooth exterior and more widely spaced radial ribs.

###### Discussion.

A nomen nudum “*Alycaeus perakensis var. minor*” was used for this subspecies in the ZRC collection. *A. kurauensis* sp. n. is quite consistent in all aspects of shell shape except in the ultimate whorl, which varies in the degree of expansion but never as expanded as *A. perakensis*.

##### Alycaeus liratulus

Taxon classificationAnimaliaGastropodaAlycaeidae

Preston, 1907

**Figure 23.**
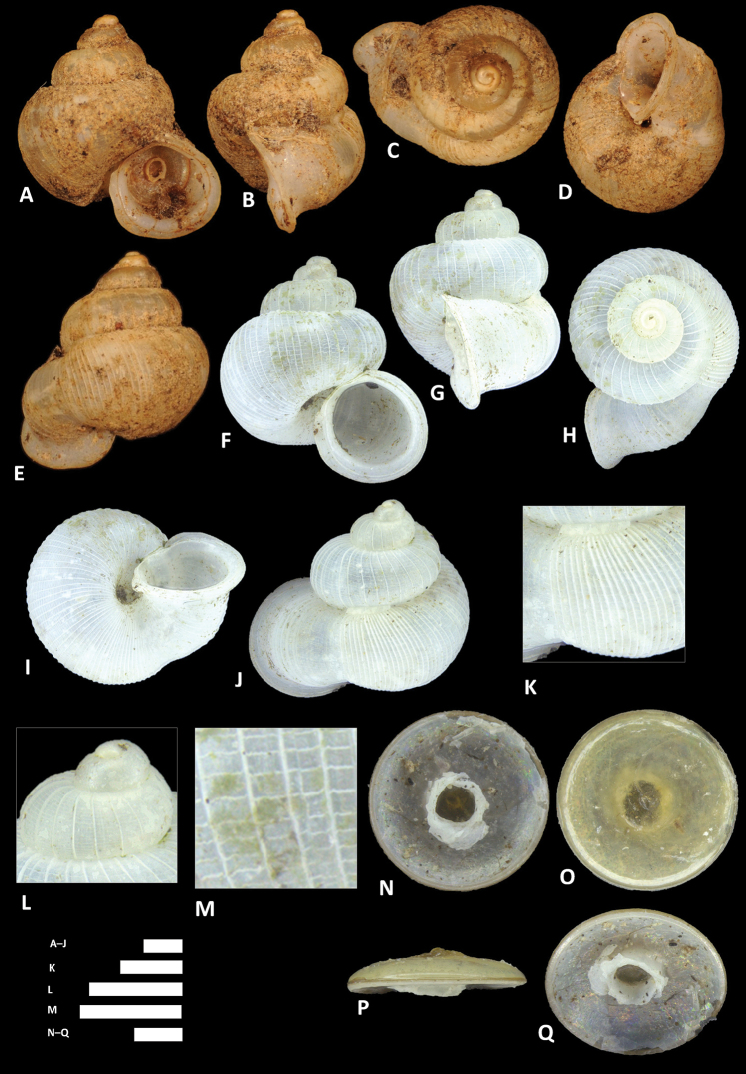
*Alycaeus liratulus* (Preston, 1907). **A–E** Shell of syntype, ANSP 99391 **F–J** Shell of BOR/MOL 6831 **K–M** Close up of shell of BOR/MOL 8357 **N–Q** Operculum of BOR/MOL 6832. Scale bars: **A–J** 1 mm; **K, L** 1 mm; **M, N–Q** 0.5 mm. Photographs **A–E** by the Malacology Department, ANSP. Photographs **F–Q** by Junn Kitt Foon.

Figures 7C , 23 , 31B

Alycaeus (Pincerna) liratula Preston, 1907: 206; Robertson et al. 1987: 4; Tarruella and Domènech 2011: 72; Mienis 2014: 37.
Alycaeus (Alycaeus) liratulus : Laidlaw 1928: 35; Benthem Jutting 1959: 77; Maassen 2001: 22; Clements et al. 2008: 2760.
Alycaeus liratulus : Marwoto 2016: 17.
Pincerna liratula : Páll-Gergely 2017: 213–219, figure 1C.

###### Type locality.

Kelantan.

###### Type material.

*Syntypes.* Ke-lan-tan (=Kelantan): ANSP 99391/1 (Seen), NHMUK 1907.5.20.191-192/2 (1 specimen seen), HUJ 22445 (Not seen).

###### Other examined materials.

Mykarst-186 Gua Madu, Kelantan: BOR/MOL 6826/2. KTN 136 Gua Musang, Kelantan: ZRC 1975.2.22.4-7/4. KTN 176 Batu Lesong, Kelantan: BOR/MOL 6831/1. KTN 149 unnamed hill, Kelantan: BOR/MOL 6832/1, BOR/MOL 8334/3. Mykarst-168, Kelantan: BOR/MOL 8411/2.

###### Description.

**Protoconch.** Smooth.

###### Shell shape and size.

Globose. Shell height: 5.62–6.70 mm. Shell width: 5.195–6.08 mm.

**Spire.** Spire height: 1.42–2.01 mm. Spire width: 2.30–2.83 mm. Number of whorls: up to 4 ⅛. Spire shape: oblong conical. Whorl periphery rounded. Umbilicus open.

**Whorl constriction.** At about 3 ¾ whorls posterior of protoconch.

**Breathing tube.** Length: 1.13–1.25 mm.

**Aperture and peristome.** Aperture circular, moderately expanded. Aperture height: 2.78–3.16 mm. Aperture width: 2.88–3.43 mm. Peristome double, thickened, slightly notched at suture. No interspace. Peristome orientation 9–20° oblique with respect to the coiling axis.

**Spiral lines.** Distinct. Regularly spaced. Approximately 11–14 lines per 1 mm.

**Radial ribs running anterior of breathing tube.** Radial ribs pronounced, evenly spaced. Approximately 6–7 ribs per 1 mm.

**Radial ribs running perpendicular to breathing tube.** Radial ribs pronounced, evenly spaced. Approximately 9–12 ribs per 1 mm.

**Radial ribs running posterior of breathing tube.** Radial ribs absent immediately posterior of constriction, becoming distinct and unevenly spaced at the middle and then absent again prior to aperture.

**Operculum.** Concave, rounded. Exterior covered by calcareous layer. Exterior nucleus with calcareous, cup-like projection. Interior covered by proteinaceous layer, smooth, mamillated.

**Shell colour.** Off white, apical whorls occasionally pinkish white.

**Living animal.** Body maroon. Head maroon. Tentacles red or brown tipped.

###### Habitat and ecology.

Lives on shrubs close to the ground. In forested areas of limestone hills.

###### Distribution.

Restricted to central and southern Kelantan. Elsewhere, isolated records in northern Sumatra, Indonesia (Benthem Jutting 1959, Marwoto 2016) need to be verified.

###### Differential diagnosis.

*Alycaeus liratulus* is most similar to *Alycaeus thieroti* in shell shape but differs in having a larger shell (larger by about 1 mm in shell height, 0.8 mm in shell width), wider-spaced radial ribs and spiral lines, longer breathing tube (longer by 0.65 mm) and flat operculum with cup-shaped calcareous extension at the nucleus exterior.

###### Discussion.

Confusion between *A. liratulus* and *A. thieroti* due to similarity in shell shape and poor original descriptions have led to many misidentifications in the past (see Discussion in *Alycaeus thieroti*). Isolated records of *A. liratulus* from northern Sumatra (Benthem Jutting 1959, Marwoto 2016) have to be re-examined. Laidlaw (1928) considers *A. liratulus* as closely related to the *A. globosus* of Borneo, an opinion we concur based on its globose shell. However, *A. globosus* differs from *A. liratulus* in the arrangement and density of radial ribs, short breathing tube and absence of a cup-shaped projection at the operculum exterior.

Supraspecific classification was attempted for *A. liratulus* by Preston (1907), who created a new subgenus Pincerna based on the sole diagnostic character of the cup-shaped protrusion on the operculum exterior of *A. liratulus*. However, the operculum structure is an unsuitable diagnostic character because analogous structures have also been found in *Alycaeus kukenthali* Sarasin & Sarasin, 1899 and *Alycaeus ochraceus* Godwin-Austen, 1893, two species unrelated in shell shape to *A. liratulus* (Sarasin and Sarasin 1899, Laidlaw 1928, Páll-Gergely 2017). Note that we maintained *Pincerna* as a subgenus of *Alycaeus* although *Pincerna* was recently elevated to genus level (Páll-Gergely 2017). See Remarks section under genus *Alycaeus* for discussion.

##### Alycaeus perakensis

Taxon classificationAnimaliaGastropodaAlycaeidae

Crosse, 1879a

**Figure 24.**
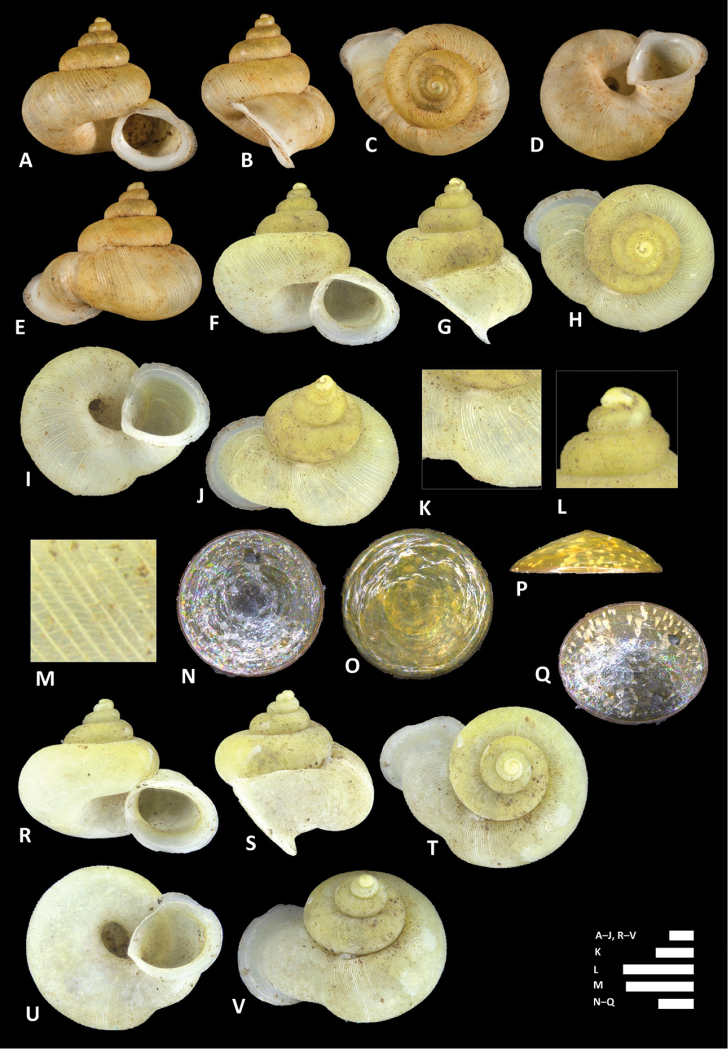
*Alycaeus perakensis* Crosse, 1879a. **A–E** Shell of syntype, MNHN-IM-2000-31793 **F–J** Shell of BOR/MOL 8350 **K–M** Close up of shell of BOR/MOL 8350 **N–Q** Operculum of BOR/MOL 8349 **R–V** Shell of BOR/MOL 8355. Scale bars: **A–J, K, L, R–V** 1 mm; **M, N–Q** 0.5 mm. Photographs **A–E** by Manuel Caballer (reproduced herein under CC-BY 4.0 license, Project RECOLNAT, MNHN). Photographs **F–V** by Junn Kitt Foon.

Figures 7S , 24 , 31V

Alycaeus perakensis Crosse, 1879a: 206–208, plate 7, figure 7; Crosse 1879b: 339–340; Möllendorff 1886: 310; Sykes 1903: 195; Tension-Woods 1888: 1068; Tielecke 1940: 345; Davison 1995: 236; Schilthuizen et al. 1999: 353; Clements et al. 2008: 2760; Foon et al. 2017: 13, figure 4F.
Alycaeus (Orthalycaeus) perakensis : Möllendorff 1891: 342; Kobelt and Möllendorff 1897: 150.
Alycaeus (Alycaeus) perakensis : Kobelt 1902: 348; Laidlaw 1928: 35; Chan 1998a: 4; Chan 1998b: 1.
Alycaeus perakensis perakensis : Maassen 2001: 23; Tarruella and Domènech 2011: 72.

###### Type locality.

Buket Pondong (= PRK 55 Gunung Pondok), Perak (4°46'57"N, 100°50'01"E).

###### Type material.

*Syntypes.* Buket Pondong (= PRK 55 Gunung Pondok), Perak: MNHN IM-2000-31793/1 (Seen), MNHN IM-2000-31794/1 (Seen), MNHN IM-2000-31795/1 (Seen).

###### Other materials.

KDH 01 Bukit Baling, Kedah: BOR/MOL 6525/1, BOR/MOL 6854/5, BOR/MOL 8527/1, BOR/MOL 8528/2. KDH 02 Gunung Pulai, Kedah: BOR/MOL 6858/2. PRK 55 Gunung Pondok, Perak: BOR/MOL 272/1, BOR/MOL 6849/1, BOR/MOL 8349/29, BOR/MOL 8350/23, BOR/MOL 11479/46, BOR/MOL 11506/31, BOR/MOL 11539/50, BOR/MOL 11563/20, ZRC 1997.25/5, ZRC 1975.2.22.175-182/12, ZRC 1975.2.22.36-66/31, ANSP 423056/2. PRK 61 Gua Badak, Perak: ZRC 1975.2.22.160-174/15. PRK 62 Gunung Dayak, Perak: BOR/MOL 277/5, BOR/MOL 6201/3, ZRC 1975.2.22.127-159/33. PRK 64 Bukit Kepala Gajah, Perak: BOR/MOL 6852/1, BOR/MOL 8354/33, BOR/MOL 8355/5, BOR/MOL 10088/31, BOR/MOL 10124/15, BOR/MOL 10136/18, BOR/MOL 10166/21, BOR/MOL 13001/2. Mykarst-171 Tasik Temenggor Hill S2, Perak: BOR/MOL 6243/1. Hill 40 m east of Sungai Siput Hospital, Perak: BOR/MOL 271/1, BOR/MOL 6877/1, BOR/MOL 8385/24, BOR/MOL 8386/1. Limestone outcrop 50m from roadside, Kampung Pahit, Klian Intan, Perak: BOR/MOL 6874/1, BOR/MOL 8379/22, BOR/MOL 8380/9. ‘Bat Cave’ Hill, roadside towards Perlop 1 and 2 oil palm plantations, Perak: BOR/MOL 6878/1, BOR/MOL 8387/17, BOR/MOL 8388/5, BOR/MOL 9770/11, BOR/MOL 9799/3, BOR/MOL 9833/1, BOR/MOL 9877/4. Batu Kebelah, Kampung Kenang Baru, Jalong Tinggi, Perak: BOR/MOL 6879/1, BOR/MOL 8389/5, BOR/MOL 8390/5, BOR/MOL 9546/23, BOR/MOL 9565/2, BOR/MOL 9573/5, BOR/MOL 9585/17. Lenggong, Perak: ZRC 1997.24/4 specimens. Perak: RMNH 153573/3, RMNH 153574/2. Banang Pupo, Yala, Thailand: ANSP 446394/3.

###### Description.

**Protoconch.** Smooth.

**Shell shape.** Conical. Shell height: 5.98–7.27 mm. Shell width: 6.48–8.62 mm.

**Spire.** Spire height: 1.83–2.64 mm. Spire width: 2.80–3.39 mm. Number of whorls: up to 4 ½. Spire shape: oblong conical. Whorl periphery rounded. Umbilicus open.

**Whorl constriction.** At about 4 ¼ whorls posterior of protoconch.

**Breathing tube.** Length: 0.61–0.88 mm.

**Aperture and peristome.** Aperture circular, expanded. Aperture height: 3.06–4.32 mm. Aperture width: 2.86–4.10 mm. Peristome double, not thickened, notched at suture. Peristome orientation 43–49° oblique with respect to the coiling axis.

**Spiral lines.** Distinct. Regularly spaced. Approximately 17–35 lines per 1 mm.

**Radial ribs running anterior of breathing tube.** Radial ribs pronounced, evenly spaced. Approximately 8–14 ribs per 1 mm.

**Radial ribs running perpendicular to breathing tube.** Radial ribs pronounced, white and thicker than those anterior of breathing tube, evenly spaced. Approximately 9–15 ribs per 1 mm.

**Radial ribs running posterior of breathing tube.** Radial ribs pronounced, widely and regularly spaced, approximately 9–16 ribs per 1 mm. Prior to aperture, radial ribs become absent.

**Operculum.** Concave, rounded. Exterior covered with thin calcareous layer, surface finely granulated with a scattered arrangement of calcareous spikes. Interior covered with proteinaceous coating, smooth.

**Shell colour.** Whorls yellow. Peristome white.

**Living animal.** Body cream-white. Head pink. Tentacles brown.

###### Habitat and ecology.

Lives on both dry and wet limestone walls. Some individuals have been spotted on tree trunks and fallen logs beside limestone cliffs. In shady forests on limestone hills.

###### Distribution range and habitat.

Restricted to central and northern Perak as well as northeastern Kedah (Bukit Baling and Gunung Pulai). Elsewhere, in Yala, Thailand (Sykes 1903, Laidlaw 1928, ANSP 446394).

###### Differential diagnosis.

*Alycaeus perakensis* is most similar to *Alycaeus roebeleni* in conical shell shape but differs in being smaller (smaller by about 0.97 mm in shell height, 1.96 mm in shell width), aperture margins more circular and has a less expanded ultimate whorl. *Alycaeus perakensis* is distinguished from *Alycaeus kapayanensis* and *Alycaeus kurauensis* sp. n. by its much larger shell (larger by about 1.43 mm in shell height, 2.32 mm in shell width), more expanded penultimate whorl, operculum exterior with spikes and more oblique peristome. Shells of *A. perakensis* vary in the degree of expansion of the ultimate whorl.

###### Discussion.

Historically, the lack of access to type specimens and poor original diagnosis have led to the misidentification of *A. selangoriensis* sp. n., *A. clementsi* sp. n. and *A. kapayanensis* as *A. perakensis* (Laidlaw 1932, van Bethem Jutting 1960a, Clements et al. 2008). *Alycaeus perakensis* shells are consistent in shell size and spire height but varies in the degree of expansion of the ultimate whorl with no particular geographic pattern. Tielecke (1940) used *A. perakensis* as a representative species for *Alycaeus* in his comparison of cyclophorid reproductive anatomy. Möllendorff (1894) considered *A. perakensis* to be closely related to *A. roebeleni*. *Alycaeus perakensis* shells were also collected from Biserat and Banang Pupo, Yala, southern Thailand (Sykes 1903, Laidlaw 1928, ANSP 446394).

##### Alycaeus regalis sp. n.

Taxon classificationAnimaliaGastropodaAlycaeidae

http://zoobank.org/B8E836AE-F3D5-47B8-BA69-B7E866DCC10B

**Figure 25.**
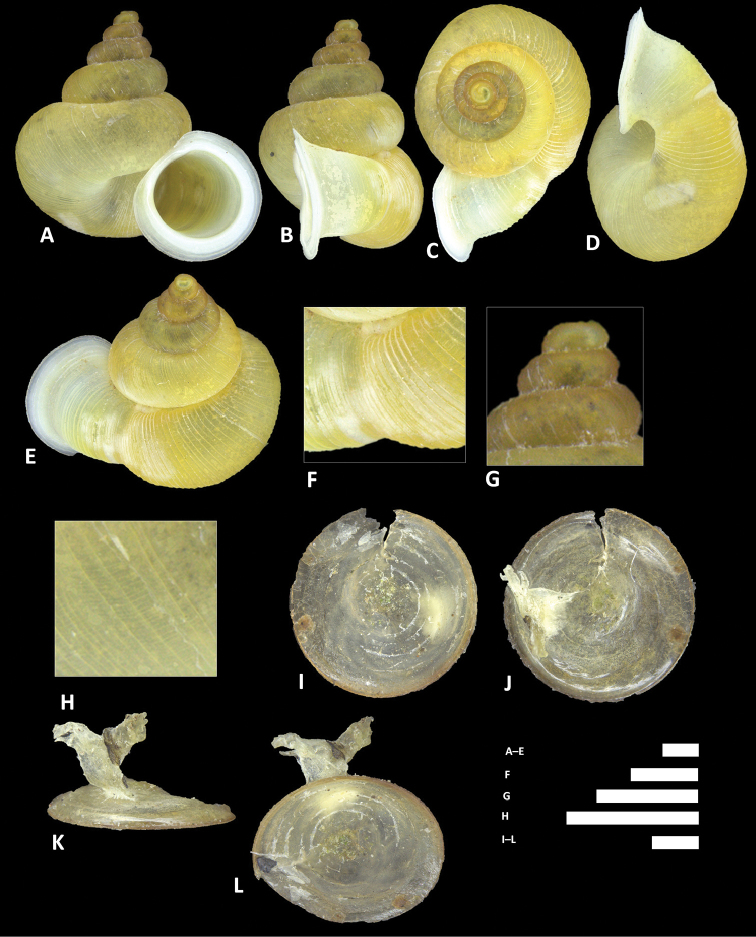
*Alycaeus regalis*, sp. n. **A–E** Shell of holotype, BOR/MOL 12990 **F–H** Close up of shell of holotype, BOR/MOL 12990 **I–L** Operculum of paratype, BOR/MOL 6881. Note operculum with proteinaceous interior extension. Scale bars: **A–E, F, G, H** 1 mm; **I–L** 0.5 mm. All photographs by Junn Kitt Foon.

Figures 7T , 25 , 31T

Alycaeus perakensis altispirus : Tarruella and Domènech 2011: figure 2E. [not Alycaeus altispirus (Möllendorff, 1902)]

###### Type locality.

PHG 02 Gunung Senyum, Pahang (3°41'50"N, 102°26'04"E).

###### Type material.

*Holotype.* PHG 02 Gunung Senyum, Pahang: BOR/MOL 12990. *Paratypes.* PHG 02 Gunung Senyum, Pahang: BOR/MOL 12991/11. PHG 03 Gunung Jebak Puyuh, Pahang: BOR/MOL 6629/14.

###### Other examined materials.

PHG 02 Gunung Senyum, Pahang: BOR/MOL 6232/3, BOR/MOL 6240/4, BOR/MOL 6881/1, BOR/MOL 8393/5, ZRC 1975.2.22.581-600/20. PHG 03 Gunung Jebak Puyuh, Pahang: BOR/MOL 6883/1.

###### Etymology.

Latin, meaning royal. In reference to the species’ tall spire and colour reminiscent of the yellow royal parasol which is part of the regalia of Southeast Asian kingdoms.

###### Description.

**Protoconch.** Smooth.

**Shell shape.** Conical. Shell height: 6.57–7.18 mm. Shell width: 5.86–6.87 mm.

**Spire.** Spire height: 2.32–2.90 mm. Spire width: 2.88–3.21 mm. Number of whorls: up to 5 ½. Spire shape: oblong conical. Whorl periphery rounded. Umbilicus open.

**Whorl constriction.** At about 5 ¼ whorls posterior of protoconch.

**Breathing tube.** Length: 0.62–0.81 mm.

**Aperture and peristome.** Aperture circular, very expanded. Aperture height: 2.91–3.66 mm. Aperture width: 2.95–3.59 mm. Peristome double, sometimes thickened, slightly notched at suture. Peristome orientation 4–17° oblique with respect to the coiling axis.

**Spiral lines.** Regularly spaced. Approximately 20–36 lines per 1 mm.

**Radial ribs running anterior of breathing tube.** Radial ribs pronounced, evenly spaced. Approximately 7–15 ribs per 1 mm.

**Radial ribs running perpendicular to breathing tube.** Radial ribs pronounced, white and thicker than those anterior of breathing tube, evenly spaced. Approximately 6–15 ribs per 1 mm.

**Radial ribs running posterior of breathing tube.** Radial ribs pronounced and uneven immediately posterior of constriction, becoming pronounced and evenly spaced from the middle section until prior to aperture. Approximately 8–16 ribs per 1 mm.

**Operculum.** Flat. Exterior with proteinaceous coating and indistinct appressed multilamellae. Interior with proteinaceous coating, smooth, mamillated. Operculum interior sometimes with a hard, ligament-like proteinaceous extension embedded within the columellar muscle of the animal when alive.

**Shell colour.** Whorls yellow. Peristome white.

**Living animal.** Body cream-white. Head pinkish-brown. Tentacle tips brown.

###### Habitat and ecology.

This species lives on dry rock surfaces covered with powdery lichens. They never appear on wet and mossy sections of limestone rocks.

###### Distribution range.

Restricted to limestone karsts at central Pahang (Gunung Senyum and Gunung Jebak Puyuh) only.

###### Differential diagnosis.

*Alycaeus regalis* sp. n. is most similar to *A. altispirus* in shell size (shell height: 6.40–7.18 mm, shell width: 5.86–6.87 mm) and tall shell spire (spire height: 2.32–2.90 mm) but differs in the ultimate whorl periphery being rounder, upper palatal section lacking the posterior folding and peristome orientated parallel to the coiling axis.

###### Discussion.

Prior to this study, specimens of *A. regalis* sp. n. from Gunung Senyum were always labelled as *Alycaeus perakensis altispirus* (Tarruella and Domènech 2011; ZRC collection lots) even though they clearly differ from each other, especially in the aperture shape. *Alycaeus regalis* sp. n. occur sympatrically with *A. costacrassa* sp. n. and *A. senyumensis* sp. n. although the two are separated by their habitat preferences. *Alycaeus regalis* sp. n. appear to prefer drier habitats whereas *A. costacrassa* sp. n. and *A. senyumensis* sp. n. are always found on wet rocks with mosses.

##### Alycaues roebeleni

Taxon classificationAnimaliaGastropodaAlycaeidae

Möllendorff, 1894

Figures 26 , 31W

Alycaeus roebeleni Möllendorff, 1894: 146–156; Tarruella and Domènech 2011: 73, figure 3A.
Alycaeus (Alycaeus) roebelini [sic]: Laidlaw 1928: 35.
Alycaeus (Alycaeus) roebeleni : Zilch 1957: 147, plate 6, figure 27.

###### Type locality.

Samui Islands (= Koh Samui archipelago), Thailand.

###### Type material.

*Lectotype*. Koh Samui, Thailand: SMF 109317 (Seen).

###### Other examined materials.

PRS 11 Bukit Tok Seri, Perlis: BOR/MOL 6863/1, BOR/MOL 8368/2. PRS 17 Gunung Chabang, Perlis: BOR/MOL 6230/1, BOR/MOL 6869/1, BOR/MOL 6873/6, BOR/MOL 8374/8. PRS 19 Bukit Chuping, Perlis: BOR/MOL 6859/1, BOR/MOL 8363/30, BOR/MOL 12980/1, ZRC 1975.2.22.99-126/29. Koh Samui, Thailand: RMNH 153575/2, ZMA 317517/1, ANSP 81380/2. Mountain north of Phatthalung, Thailand: ANSP 446393/1.

###### Description.

**Protoconch.** Smooth.

**Shell shape.** Conical. Shell height: 7.20–8.01 mm. Shell width: 8.94–10.08 mm.

**Spire.** Spire height: 2.17–2.74 mm. Spire width: 3.13–3.68 mm. Number of whorls: up to 4 ½. Spire shape: oblong conical. Whorl periphery rounded. Umbilicus open.

**Whorl constriction.** At about 4 ¼ whorls posterior of protoconch.

**Breathing tube.** Length: 0.71–0.94 mm.

**Aperture and peristome.** Aperture circular, very expanded. Aperture height: 4.39–5.21 mm. Aperture width: 4.08–5.01 mm. Peristome single to double, not thickened. Peristome orientation 44–49° oblique with respect to the coiling axis.

**Spiral lines.** Indistinct. Regularly spaced. Approximately 23–37 lines per 1 mm.

**Radial ribs running anterior of breathing tube.** Radial ribs pronounced, evenly spaced. Approximately 8–9 ribs per 1 mm.

**Radial ribs running perpendicular to breathing tube.** Radial ribs pronounced, evenly spaced. Approximately 7–10 ribs per 1 mm.

**Radial ribs running posterior of breathing tube.** Radial ribs pronounced, unevenly spaced immediately posterior of constriction.

**Operculum.** Concave, rounded. Exterior covered with thin calcareous layer, smooth. Interior covered with thick proteinaceous coating, smooth, mamillated.

**Shell colour.** Yellow at apical whorls. Either fades to white towards ultimate whorl or remain yellow throughout.

**Living animal.** Unknown.

###### Habitat and ecology.

Lives on limestone rock crevices covered by mosses. In shady forests on limestone hills.

###### Distribution range.

Restricted to limestone hills of the Chuping geological formation in central Perlis. Elsewhere, in Koh Samui and Phatthalung, southern Thailand (Möllendorff 1894, materials examined).

###### Differential diagnosis.

*Alycaeus roebeleni* is most similar to *A. perakensis* in shell shape but is larger (larger by about 0.97 mm in shell height, 1.96 mm in shell width), spire taller and wider (taller by about 0.22 mm in spire height, wider by about 0.31 mm in spire width), penultimate whorl wider as well as peristome more expanded (Möllendorff 1894). *Alycaeus roebeleni* also differs from *A. perakensis* in its operculum, which has a smooth exterior and thick, brown proteinaceous layer at the interior. The spire height of *A. roebeleni* is quite variable across its range but never as tall as *A. perakensis*.

###### Discussion.

This species was considered closely related to *A. perakensis* by Möllendorff (1894), which we concur with, based on examined specimens. However, contrary to the Möllendorff (1894) claim that the operculum is entirely proteinaceous, we show that *A. roebeleni* has proteinaceous interior and calcareous exterior layers. Despite clear distinctions made between *A. roebeleni* and *A. perakensis* in Möllendorff (1894), the two species remain confused by past workers. An *A. roebeleni* collection lot in ZRC was originally labelled as *A. perakensis* (ZRC 1975.2.22.99-126). A specimen labelled as *A. roebeleni* from Banang Pupo, Yala, Thailand is actually *A. perakensis* (ANSP 446394). *Alycaeus roebeleni minor* Möllendorff, 1894, from Biserat, Yala, Thailand is also likely *A. perakensis* (see Remarks in *A. perakensis*). The Perlis population appears to be the southern-end of the distribution range for *A. roebeleni*.

**Figure 26.**
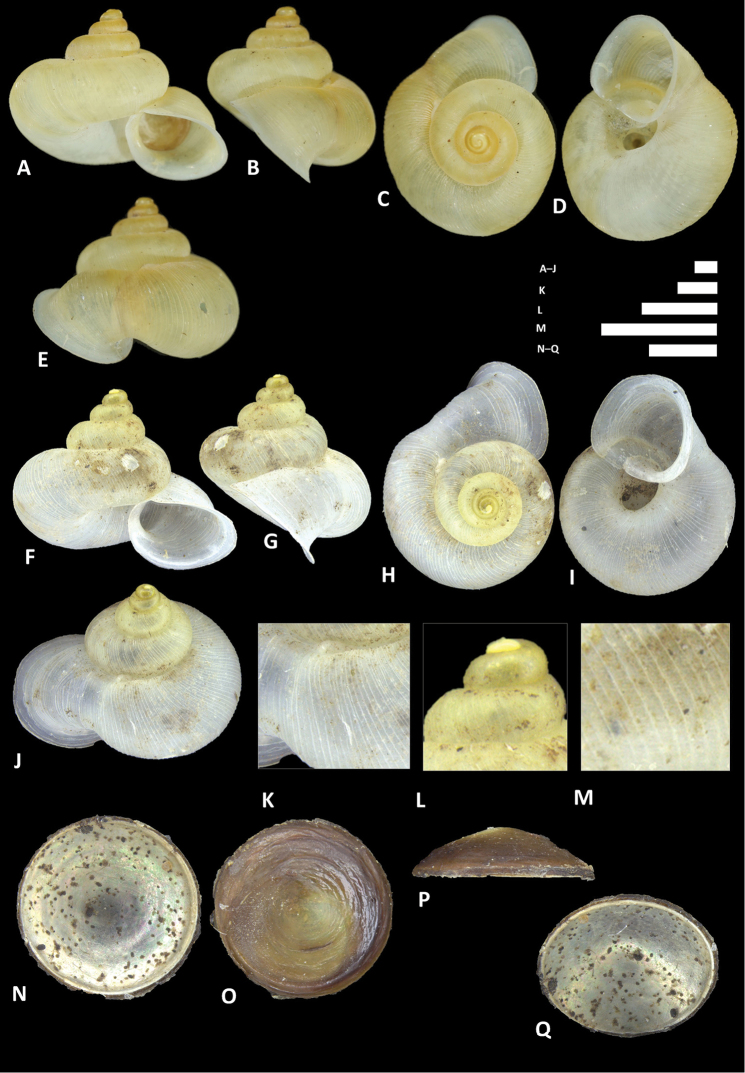
*Alycaeus roebeleni* Möllendorff, 1894. **A–E** Shell of syntype, SMF 109317 **F–J** Shell of BOR/MOL 8363 **K–M** Close up of shell of BOR/MOL 8363 **N–Q** Operculum of BOR/MOL 8363. Scale bars: **A–J, K, L, M, N–Q** 1 mm. Photographs **A–E** by Páll-Gergely Barna, courtesy of R. Janssen, SMF. Photographs **F–Q** by Junn Kitt Foon.

##### Alycaeus selangoriensis sp. n.

Taxon classificationAnimaliaGastropodaAlycaeidae

http://zoobank.org/B7390E20-CC8B-4893-BC8B-7DB15521E8CC

**Figure 27.**
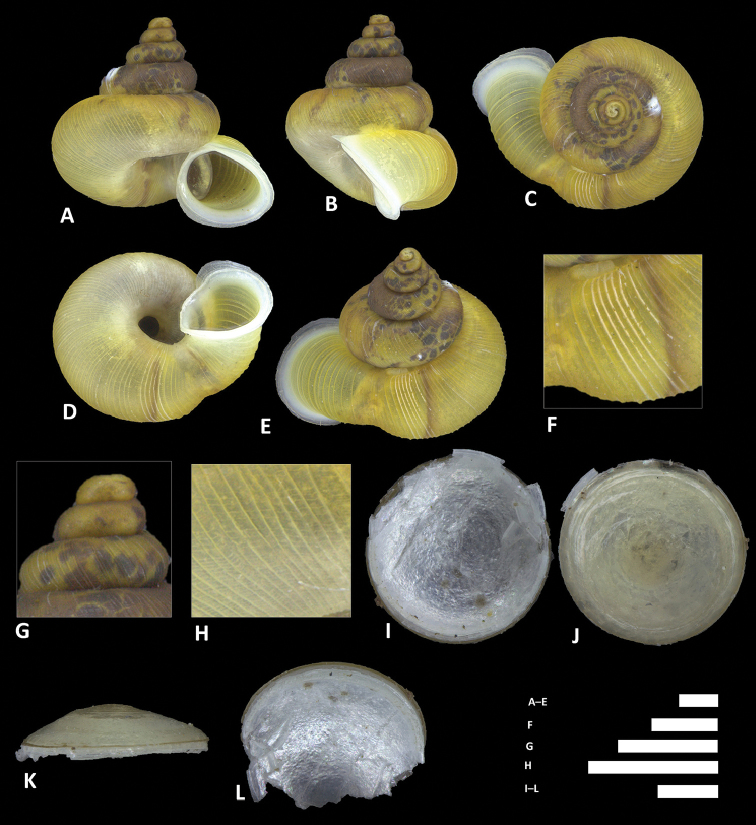
*Alycaeus selangoriensis* sp. n. **A–E** Shell of holotype, BOR/MOL 12988 **F–H** Close up of shell of holotype, BOR/MOL 12988 **I–L** Operculum of paratype, BOR/MOL 6808. Scale bars: **A–E, F, G, H** 1 mm; **I–L** 0.5 mm. All photographs by Junn Kitt Foon.

Figures 7U , 27 , 31O

Alycaeus perakensis : Laidlaw 1932: 37. [not Alycaeus perakensis Crosse, 1879a]
Alycaeus (Alycaeus) perakensis : Chan 1997: 37. [not Alycaeus perakensis Crosse, 1879a]
Alycaeus kapayanensis : Venmans 1956: 83. [not Alycaeus kapayanensis Morgan, 1885b]
Alycaeus perakensis var. minor nomen nudum, Clements et al. 2008: 2760.

###### Type locality.

SGR 01 Batu Caves, Selangor (3°14'17"N, 101°41'02"E).

###### Type material.

*Holotype.* SGR 01 Batu Caves, Selangor: BOR/MOL 12988. *Paratypes.* SGR 01 Batu Caves, Selangor: BOR/MOL 6808/1, BOR/MOL 8314/9, BOR/MOL 12989/1. SGR 02 Bukit Takun, Selangor: BOR/MOL 6810/7, BOR/MOL 8318/2.

###### Other examined materials.

SGR 01 Batu Caves, Selangor: BOR/MOL 268/2, BOR/MOL 6371/>1, BOR/MOL 8315/24, ZRC 1975.2.22.285-382/98, ZRC 1997.23/5, RMNH 153568/16, ZMA 33474/1. SGR 02 Bukit Takun, Selangor: BOR/MOL 6627/14, BOR/MOL 8317/6, ZRC 1975.2.22.190-228/39, ZRC 1975.2.24.403-439/37, ZRC 1997.22/5.

###### Etymology.

Named after Selangor, the state that the type locality of this species is part of.

###### Description.

**Protoconch.** Smooth.

**Shell shape.** Conical. Shell height: 5.07–5.44 mm. Shell width: 5.06–5.88 mm.

**Spire.** Spire height: 1.81–2.00 mm. Spire width: 2.35–2.50 mm. Number of whorls: up to 5 ¼. Spire shape: oblong conical. Whorl periphery rounded. Umbilicus open.

**Whorl constriction.** At about 5 whorls posterior of protoconch.

**Breathing tube.** Length: 0.52–0.71 mm.

**Aperture and peristome.** Aperture circular, expanded. Aperture height: 2.68–2.37 mm. Aperture width: 2.33–2.61 mm. Peristome double, not thickened, upper palatal section folded posteriorly into a wing-like structure, notched at suture. Peristome orientation 35–40° oblique with respect to the coiling axis.

**Spiral lines.** Distinct. Regularly spaced. Approximately 20–33 lines per 1 mm.

**Radial ribs running anterior of breathing tube.** Radial ribs pronounced, evenly spaced. Approximately 7–20 ribs per 1 mm.

**Radial ribs running perpendicular to breathing tube.** Radial ribs pronounced, white and thicker than those anterior of breathing tube, evenly spaced. Approximately 11–21 ribs per 1 mm.

**Radial ribs running posterior of breathing tube.** Radial ribs absent immediately posterior of constriction, becoming pronounced and evenly spaced at the middle until prior to aperture. Approximately 9–14 ribs per 1 mm.

**Operculum.** Concave, rounded. Exterior covered with thick but flaky calcareous layer, surface finely granulated. Interior proteinaceous layered, smooth, with indistinct mamilla.

**Shell colour.** Whorls yellow. Peristome white.

**Living animal.** Body cream-white. Head pinkish-brown. Tentacles reddish-brown.

###### Habitat and ecology.

Lives on wet limestone walls covered with mosses, in forested areas. It can also be found in areas with substantial human disturbance such as on the limestone walls of the temple at Batu Caves.

###### Distribution.

Restricted to Batu Caves and Bukit Takun, Selangor.

###### Differential diagnosis.

*Alycaeus selangoriensis* sp. n. is most similar to *A. kapayanensis* but differs in having a consistently larger shell (larger by about 0.96 mm in shell height, 1.6 mm in shell width), more expanded ultimate whorl and thicker operculum with calcareous exterior. The upper palatal section of the peristome in *A. selangoriensis* sp. n. is always folded posteriorly and downwards, creating a wing-like extension prior to the notch at the suture, a feature not present in the simple circular expanded peristome of *A. kapayanensis*. *Alycaeus selangoriensis* sp. n. resembles *A. perakensis* in having a moderately expanded ultimate whorl but differs in being smaller shelled (smaller by about 1.37 mm in shell height, 2.08 mm in shell width).

###### Discussion.

Historically, *Alycaeus selangoriensis* sp. n. shells have been assigned to superficially similar congeners *A. perakensis* and *A. kapayanensis* (Laidlaw 1932, Venmans 1956, Chan 1997, ZRC and RMNH collection lots). *Alycaeus selangoriensis* sp. n. has also been labelled as “*Alycaeus kapayanensis var. minor*”, a nomen nudum (Clements et al. 2008, ZRC collection lots). *Alycaeus selangoriensis* sp. n. is morphologically consistent in having a very expanded ultimate whorl but varies in spire height. Venmans (1956) described and found that the radula and jaw of *A. selangoriensis* sp. n. differ substantially from *A. thieroti* and *A. conformis*.

##### Alycaeus senyumensis sp. n.

Taxon classificationAnimaliaGastropodaAlycaeidae

http://zoobank.org/78ADBCEF-599F-41D9-8CBE-C76DA77683ED

**Figure 28.**
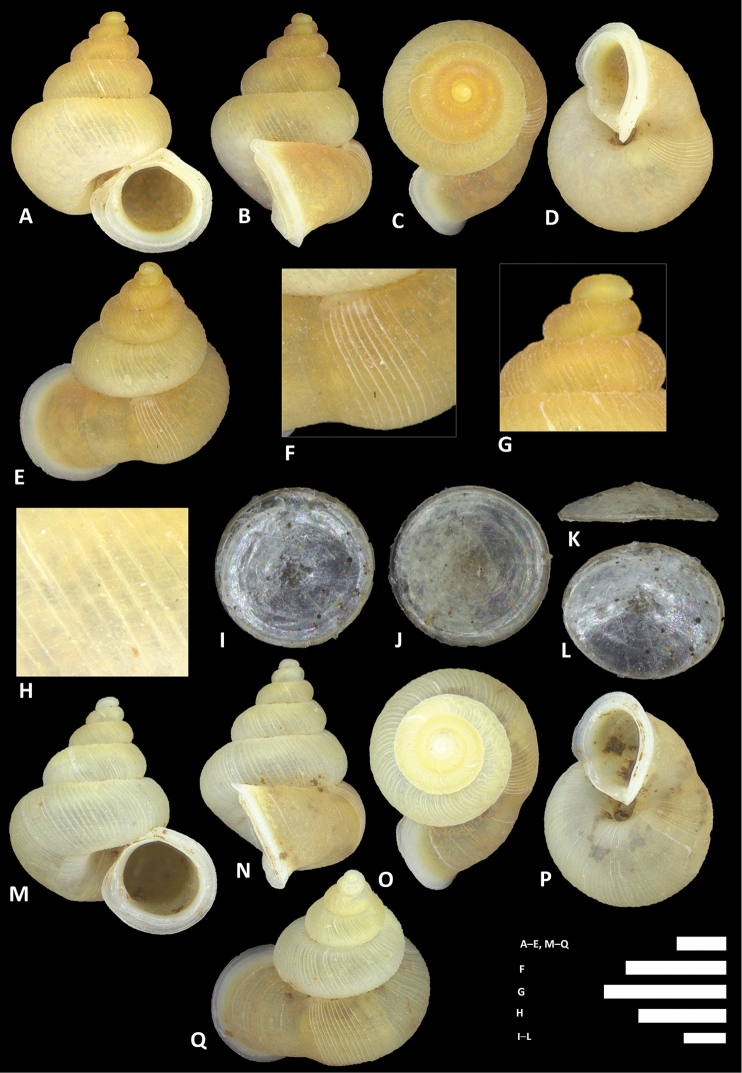
*Alycaeus senyumensis* sp. n. **A–E** Shell of holotype, BOR/MOL 12965 **F–H** Close up of shell of holotype, BOR/MOL 12965 **I–L** Operculum of paratype, BOR/MOL 6880 **M–Q** Shell of paratype, operculum of BOR/MOL 12966. Scale bars: **A–E, F, G, M–Q** 1 mm; **H, I–L** 0.5 mm. All photographs by Junn Kitt Foon.

Figures 7V–W , 28 , 31R

Alycaeus kelantanensis : Clements et al. 2008: 2760.

###### Type locality.

PHG 02 Gunung Senyum, Pahang (3°41'50"N, 102°26'04"E).

###### Type material.

*Holotype.* PHG 02 Gunung Senyum, Pahang: BOR/MOL 12965. *Paratypes.* PHG 02 Gunung Senyum, Pahang: BOR/MOL 6880/2, BOR/MOL 12966/69. PHG 03 Gunung Jebak Puyuh, Pahang: BOR/MOL 8395/6, BOR/MOL 12964/2.

###### Other examined materials.

PHG 02 Gunung Senyum, Pahang: BOR/MOL 275/1, BOR/MOL 6195/6, BOR/MOL 6239/4, BOR/MOL 6249/11, BOR/MOL 8391/41, BOR/MOL 8392/29, ZRC 1975.2.22.444-543/100, ZRC 1975.2.22.626-632/7. PHG 03 Gunung Jebak Puyuh, Pahang: BOR/MOL 6630/13, BOR/MOL 8394/27.

###### Etymology.

Named after the type locality, Gunung Senyum.

###### Description.

**Protoconch.** Smooth.

**Shell shape.** Conical. Shell height: 4.49–5.01 mm. Shell width: 3.96–4.41 mm.

**Spire.** Spire height: 1.47–1.94 mm. Spire width: 2.05–2.28 mm. Number of whorls: up to 5 ¼. Spire shape: oblong conical. Whorl periphery rounded. Umbilicus partially closed.

**Whorl constriction.** At about 5 whorls posterior of protoconch.

**Breathing tube.** Length: 0.44–0.57 mm.

**Aperture and peristome.** Aperture circular, very expanded especially at columellar section. Aperture height: 2.17–2.36 mm. Aperture width: 2.02–2.20 mm. Peristome double, thickened, upper palatal section folded posteriorly into a wing-like structure, notched at suture. Peristome orientation 21–28° oblique with respect to the coiling axis.

**Spiral lines.** Indistinct. Regularly spaced. Approximately 14–24 lines per 1 mm.

**Radial ribs running anterior of breathing tube.** Radial ribs pronounced, evenly spaced. Approximately 8–15 ribs per 1 mm.

**Radial ribs running perpendicular to breathing tube.** Radial ribs pronounced, white and thicker than those anterior of breathing tube, evenly spaced. Approximately 14–21 ribs per 1 mm.

**Radial ribs running posterior of breathing tube.** Radial ribs unevenly spaced, sometimes distinct but usually absent.

**Operculum.** Concave, conical. Exterior covered with thin calcareous layer, surface finely granulated. Interior covered with proteinaceous layer, smooth.

**Shell colour.** Whorls yellow or brownish-red. Peristome white.

**Living animal.** Body cream-white. Head pink. Tentacles yellow or red.

###### Habitat and ecology.

Lives on both wet, mossy limestone rock walls and boulders. In shady forests on limestone hills. Some living individuals were observed dangling on mucous threads under rock overhangs.

###### Distribution range.

Restricted to limestone karsts at central Pahang (Gunung Senyum and Gunung Jebak Puyuh) only.

###### Differential diagnosis.

*Alycaeus senyumensis* sp. n. is distinguished from other *Alycaeus* by its peristome that is distinctly expanded and angled at the columellar section and partially obscuring the umbilicus, narrow ultimate whorl, strong radial ribs, shallow suture and small shell. *Alycaeus senyumensis* sp. n. is differentiated from the sympatric *A. regalis* sp. n. and *A. costacrassa* sp. n. by its much smaller shell and expanded peristome angled at the columellar base.

###### Discussion.

Historically, *Alycaeus senyumensis* sp. n. specimens were labelled as *A. kelantanensis* (Clements et al. 2008, ZRC collection lots). *Alycaeus senyumensis* sp. n. occur sympatrically with *A. costacrassa* sp. n. and *A. regalis* sp. n. although syntopically only with *A. costacrassa* sp. n. This is the only *Alycaeus* species we observed dangling from mucous threads during our fieldwork. Such behaviour in *Alycaeus* has been recorded in Peninsular Malaysia and Borneo before (Schilthuizen et al. 1999, Deeleman-Reinhold 2009).

##### Alycaeus thieroti

Taxon classificationAnimaliaGastropodaAlycaeidae

Morgan, 1885b

**Figure 29.**
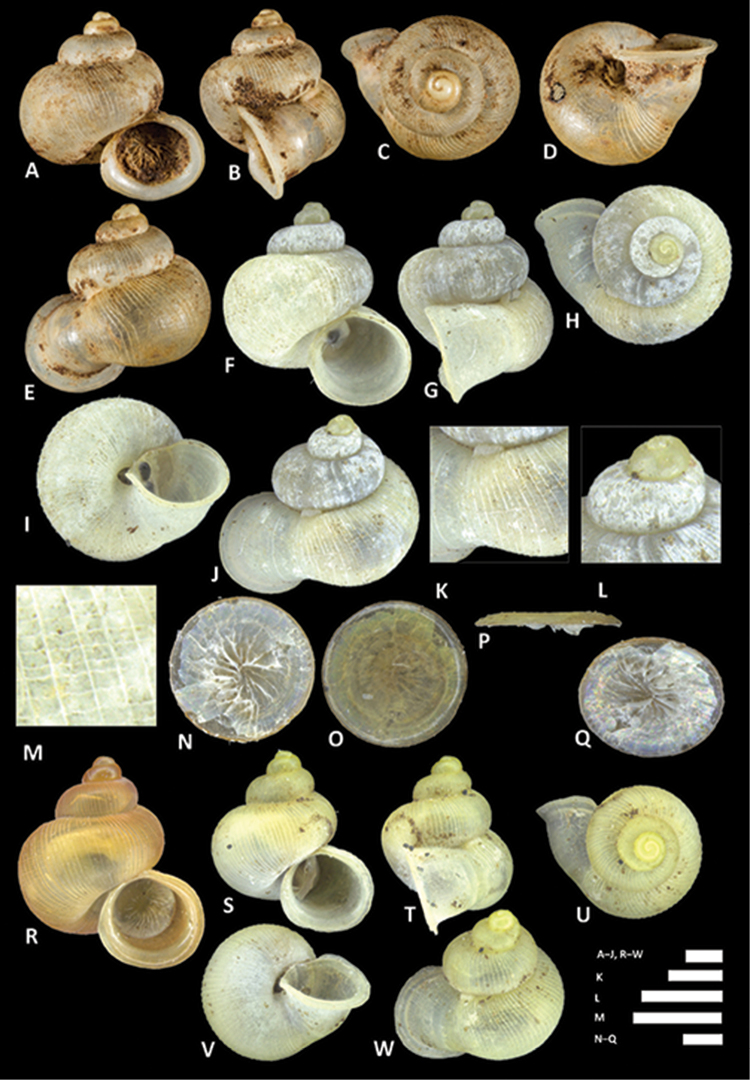
*Alycaeus thieroti* Morgan, 1885b. **A–E** Shell of syntype, MNHN-IM-2000-31799 **F–J** Shell of BOR/MOL 6835 **K–M** Close up of shell of BOR/MOL 6835 **N–Q** Operculum of BOR/MOL 6835 **R** Shell of BOR/MOL 6528a **S–W** Shell of BOR/MOL 6528b. Scale bars: **A–J, R–W** 1 mm; **K, L** 1 mm; **M, N–Q** 0.5 mm. Photographs **A–E** by Manuel Caballer (reproduced herein under CC-BY 4.0 license, Project RECOLNAT, MNHN). Photographs **F–W** by Junn Kitt Foon.

Figures 7D , 29 , 31C

Alycaeus thieroti Morgan, 1885b: 403-404, plate 10; Venmans 1956: 82, figure 3; Benthem Jutting 1960a: 13; Sykes 1903: 197; Chan 1997: 37; Maassen 2001: 23; Clements et al. 2008: 2760; Tarruella and Domènech 2011: 73; Foon et al. 2017: 13, figure 5A.
Alycaeus (Orthalycaeus) thieroti : Möllendorff 1891: 342; Kobelt and Möllendorff 1897: 151.
Alycaeus (Alycaeus) thieroti : Kobelt 1902: 352; Laidlaw 1928: 35; Laidlaw 1932: 36.
Alycaeus liratulus : Liew 2010: 101. [not Alycaeus liratulus (Preston, 1907)]
Pincerna thieroti : Páll-Gergely 2017: 213–214.

###### Type locality.

PRK 18 Gunung Lanno, Perak (4°31'31"N, 101°08'48"E).

###### Type material.

*Syntype*. Gunong Lano (= PRK 18 Gunung Lanno), Perak: MNHN IM-2000-31799/5 (1 specimen seen).

###### Other examined materials.

Endau-Kluang, Johor: BOR/MOL 6466/1, BOR/MOL 6467/1. KTN 01 Gunung Reng, Kelantan: BOR/MOL 6835/1, BOR/MOL 8335/6. KTN 95 Gua Panjang, Kelantan: BOR/MOL 12962/5. Lojing, Kelantan: BOR/MOL 6528/2. PHG 01 Kota Gelanggi, Pahang: BOR/MOL 6886/2, BOR/MOL 6636/1. PHG 02 Gunung Senyum, Pahang: BOR/MOL 12961/4. PHG 03 Gunung Jebak Puyuh, Pahang: BOR/MOL 6885/3. PHG 05 Bukit Cintamanis, Pahang: BOR/MOL 6633/1. PHG 75 Bukit Tenggek, Pahang: ZRC 1975.2.22.8-11/2. PHG 77 Bukit Mengapur, Pahang: BOR/MOL 6889/1. Unnamed limestone hill north of Jalan Lipis-Gua Musang Lama, Felda Chegar Perah I and II, Pahang: BOR/MOL 6815/1. Gunung Tangkup, Kenong Rimba, Pahang: BOR/MOL 6237/1. Gunung Kesong, Kenong Rimba, Pahang: BOR/MOL 6253/2. PRK 55 Gunung Pondok, Perak: BOR/MOL 8410/1. PRK 23 Gunung Rapat, Perak: BOR/MOL 10056/4, BOR/MOL 10258/2. PRK 62 Gunong Dayak, Perak: ZRC 1975.2.22.1/1. Mykarst-025, Perak: BOR/MOL 9415/1, BOR/MOL 12422/1. Lenggong, Perak: ZRC 1975.2.22.2-3/2. SGR 02 Bukit Takun, Selangor: ZRC 1997.27/5.

###### Description.

**Protoconch.** Smooth.

###### Shell shape and size.

Globose. Shell height: 4.55–5.68 mm. Shell width: 4.36–5.34 mm.

**Spire.** Spire height: 1.06–1.58 mm. Spire width: 1.73–2.30 mm. Number of whorls: up to 4. Spire shape: oblong conical. Whorl periphery rounded. Umbilicus open or partially closed.

**Whorl constriction.** At about 3 ⅝ whorls posterior of protoconch.

**Breathing tube.** Length: 0.40–0.53 mm.

**Aperture and peristome.** Aperture circular, moderately expanded. Aperture height: 2.28–2.76 mm. Aperture width: 2.42–2.98 mm. Peristome double, thickened, notched at suture. No interspace. Peristome orientation 15–18° oblique with respect to the coiling axis.

**Spiral lines.** Distinct. Regularly spaced. 20–37 lines per 1 mm.

**Radial ribs running anterior of breathing tube.** Radial ribs pronounced, evenly spaced. Approximately 8–15 ribs per 1 mm.

**Radial ribs running perpendicular to breathing tube.** Radial ribs pronounced, evenly spaced. Approximately 9–21 ribs per 1 mm.

**Radial ribs running posterior of breathing tube.** Ranges from absent to indistinct, unevenly spaced.

**Operculum.** Almost flat. Exterior covered by calcareous layer. Exterior has calcareous ridges radiating outwards of the nucleus. Interior covered by proteinaceous layer, smooth.

**Shell colour.** Variable – red, yellow or white.

**Living animal.** Body cream-white or maroon. Head pink. Tentacles red or brown tipped.

###### Habitat and ecology.

Lives in crevices on wet limestone boulders and shrubs close to the ground. In thickly forested areas. Lives in both limestone and non-limestone areas.

###### Distribution.

Widespread in Perak, Selangor, Kelantan and Pahang.

###### Differential diagnosis.

*Alycaeus thieroti* is most similar to *Alycaeus liratulus* in shell shape but differs in having a smaller and more elongated shell (smaller by about 1 mm in shell height, 0.8 mm in shell width), narrowly-spaced radial ribs and spiral lines, shorter breathing tube (shorter by 0.65 mm) and flat operculum with calcareous ridges radiating out from the exterior nucleus.

###### Discussion.

Morgan (1885b) first described *A. thieroti* with simple shell characters and an erroneous description of the operculum as smooth and proteinaceous. Möllendorff (1891) subsequently redescribed the species with additional shell characters but did not point out the error. The operculum is a critical diagnostic feature that clearly distinguishes *A. thieroti* from *A. liratulus*. Thus, this uncorrected error has led to the misidentification of *A. thieroti* as *A. liratulus* and vice versa (e.g. Liew 2010, ZRC collection lots). *Alycaeus thieroti* was known from Perak, Selangor and Kelantan prior to this study (Morgan 1885b, Möllendorff 1891, Sykes 1903, Laidlaw 1928, Laidlaw 1932, Benthem Jutting 1960a, Venmans 1956). *Alycaeus thieroti* in Pahang and Johor are new records. Venmans (1956) examined the radular and jaw features of *A. thieroti*, which is distinct from *A. selangoriensis* sp. n. but similar to *A. conformis* in teeth arrangement. Note that we maintained *Pincerna* as a subgenus of *Alycaeus* although *Pincerna* was recently elevated to genus level (Páll-Gergely 2017). See Remarks section under genus *Alycaeus* for discussion.

##### Alycaeus virgogravida sp. n.

Taxon classificationAnimaliaGastropodaAlycaeidae

http://zoobank.org/5A58FAD8-6020-49FE-87CB-21D245D8EEC6

Figures 30 , 31S

###### Type locality.

Limestone hill at east side of Pulau Dayang Bunting, off Langkawi Island, Kedah (6°12'26"N, 99°47'04"E).

###### Type material.

*Holotype.* Limestone hill at east side of Pulau Dayang Bunting, off Langkawi Island, Kedah: BOR/MOL 12978. *Paratypes.* Limestone hill at east side of Pulau Dayang Bunting, off Langkawi Island, Kedah: BOR/MOL 12979/1, BOR/MOL 6248/6.

###### Etymology.

Latin, meaning pregnant maiden. Named after the type locality, Pulau Dayang Bunting, meaning island of the pregnant maiden.

###### Description.

**Protoconch.** Smooth.

**Shell shape.** Conical. Shell height: 4.71–5.14 mm. Shell width: 4.44–4.97 mm.

**Spire.** Spire height: 1.68–1.77 mm. Spire width: 2.19–2.50 mm. Number of whorls: up to 4 ½. Spire shape: oblong conical. Whorl periphery rounded. Umbilicus open.

**Whorl constriction.** At about 4 ¼ whorls posterior of protoconch.

**Breathing tube.** Length: 0.49–0.51 mm.

**Aperture and peristome.** Aperture circular, expanded. Aperture height: 2.10–2.36 mm. Aperture width: 2.10–2.29 mm. Peristome double, not thickened, notched at suture. Peristome orientation 29–33° oblique with respect to the coiling axis.

**Spiral lines.** Distinct. Regularly spaced. Approximately 18–34 lines per 1 mm.

**Radial ribs running anterior of breathing tube.** Radial ribs pronounced, evenly spaced. Approximately 11–15 ribs per 1 mm.

**Radial ribs running perpendicular to breathing tube.** Radial ribs pronounced and thicker than those anterior of breathing tube, evenly spaced. Approximately 14–17 ribs per 1 mm.

**Radial ribs running posterior of breathing tube.** Radial ribs pronounced, unevenly spaced throughout.

**Operculum.** Concave, rounded. Exterior calcareous layer thick, smooth and polished. Exterior nucleus punctured through, with a cup-like projection. Four concentric ridges were distributed in the tunnel within the thick, cup-like projection. Interior calcareous, with dense concentric circles of multimellae, nucleus punctured through. Operculum edge teethed, etched with regularly spaced grooves.

**Shell colour.** Whorls yellow. Peristome white.

**Living animal.** Unknown.

###### Habitat and ecology.

Unknown.

###### Distribution range.

Known only from the type locality Pulau Dayang Bunting, Langkawi archipelago.

###### Differential diagnsosis.

*Alycaeus virgogravida* sp. n. is similar to *A. kapayanensis* in having a conical shell but is distinguished by its more expanded whorls, spacing of radial and spiral ribs, and notably, the thick, calcareous operculum with cup-shaped nucleus. *Alycaeus virgogravida* sp. n. is distinguished from the globose-shelled *Alycaeus liratulus*, the only other species in Peninsular Malaysia with cup-shaped operculum nucleus, by its conical shell.

###### Discussion.

*Alycaeus virgogravida* sp. n. is the only small, conical and yellow *Alycaeus* species in Peninsular Malaysia with cup-shaped operculum.

**Figure 30.**
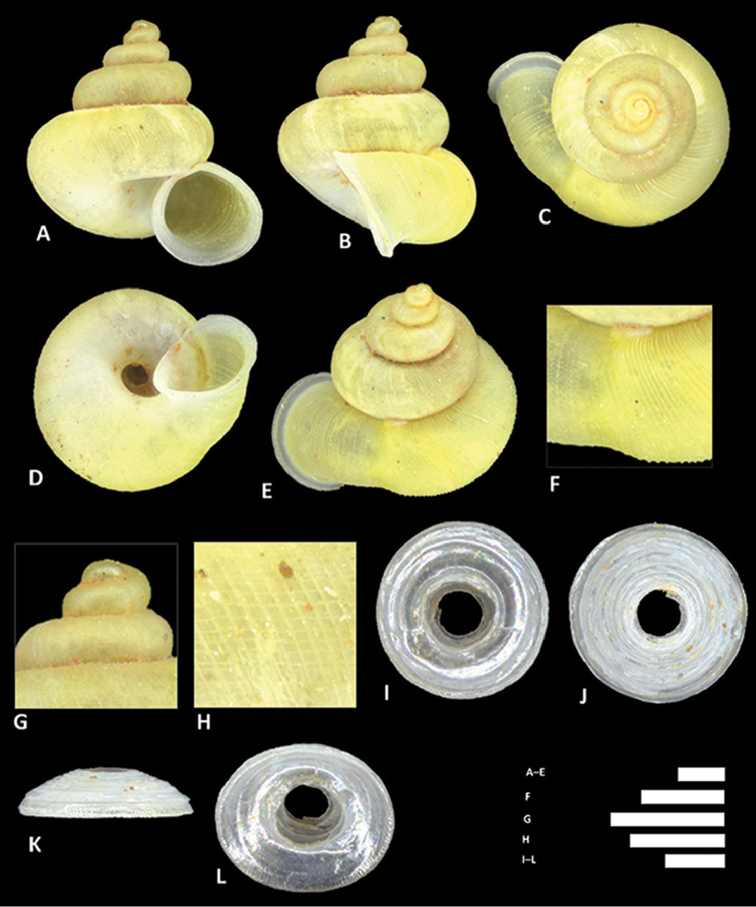
*Alycaeus virgogravida* sp. n. **A–E** Shell of holotype, BOR/MOL 12978 **F–H** Close up of shell of holotype, BOR/MOL 12978 **I–L** Operculum of holotype, BOR/MOL 12978. Scale bars: **A–E, F, G** 1 mm; **H, I–L** 0.5 mm. All photographs by Junn Kitt Foon.

**Figure 31.**
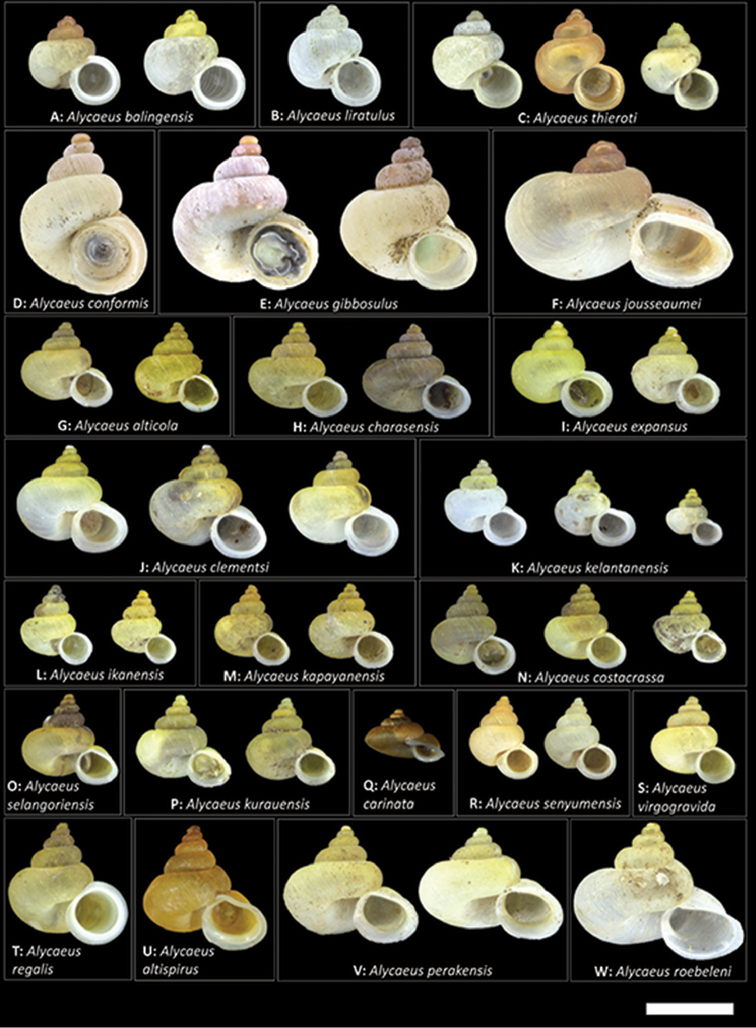
Synoptic view of the 23 *Alycaeus* taxa in Peninsular Malaysia. Scale bar: 5 mm. Photograph of *Alycaeus carinata* by Thor Seng Liew. Photograph of *Alycaeus altispirus* by Páll-Gergely Barna, courtesy of R. Janssen, SMF. All other photographs by Junn Kitt Foon.

## Supplementary Material

XML Treatment for Alycaeus

XML Treatment for Alycaeus alticola

XML Treatment for Alycaeus altis pirus

XML Treatment for Alycaeus balingensis

XML Treatment for Alycaeus carinata

XML Treatment for Alycaeus charasensis

XML Treatment for Alycaeus clementsi

XML Treatment for Alycaeus conformis

XML Treatment for Alycaeus costacrassa

XML Treatment for Alycaeus expansus

XML Treatment for Alycaeus gibbosulus

XML Treatment for Alycaeus ikanensis

XML Treatment for Alycaeus jousseaumei

XML Treatment for Alycaeus kapayanensis

XML Treatment for Alycaeus kelantanensis

XML Treatment for Alycaeus kurauensis

XML Treatment for Alycaeus liratulus

XML Treatment for Alycaeus perakensis

XML Treatment for Alycaeus regalis

XML Treatment for Alycaues roebeleni

XML Treatment for Alycaeus selangoriensis

XML Treatment for Alycaeus senyumensis

XML Treatment for Alycaeus thieroti

XML Treatment for Alycaeus virgogravida

## Supplementary materials

Supplementary material 1 List of names and coordinates of localities visited during fieldwork in 2016 Link: https://doi.org/10.3897/zookeys.692.14706.suppl1 Data type: Table in CSV file Explanation note: A CSV file containing a list of name and coordinates of localities visited during fieldwork in 2016. This dataset is made available under the Open Database License (http://opendatacommons.org/licenses/odbl/1.0/). The Open Database License (ODbL) is a license agreement intended to allow users to freely share, modify, and use this Dataset while maintaining this same freedom for others, provided that the original source and author(s) are credited.Junn Kitt Foon, Thor-Seng Liew

Supplementary material 2 List of specimen measurements used for this study Link: https://doi.org/10.3897/zookeys.692.14706.suppl2 Data type: Table in Microsoft Excel file Explanation note: A Microsoft Excel file containing a list of specimens and their measurements used in this study. This dataset is made available under the Open Database License (http://opendatacommons.org/licenses/odbl/1.0/). The Open Database License (ODbL) is a license agreement intended to allow users to freely share, modify, and use this Dataset while maintaining this same freedom for others, provided that the original source and author(s) are credited.Junn Kitt Foon, Thor-Seng Liew
